# Lactose-Functionalized Carbosilane Glycodendrimers Are Highly Potent Multivalent Ligands for Galectin-9 Binding: Increased Glycan Affinity to Galectins Correlates with Aggregation Behavior

**DOI:** 10.1021/acs.biomac.3c00426

**Published:** 2023-09-08

**Authors:** Monika Müllerová, Michaela Hovorková, Táňa Závodná, Lucie Červenková Št́astná, Alena Krupková, Vojtěch Hamala, Kateřina Nováková, Jan Topinka, Pavla Bojarová, Tomáš Strašák

**Affiliations:** † Institute of Chemical Process Fundamentals, Czech Academy of Sciences, Rozvojová 135, 165 02 Prague, Czech Republic; ‡ Institute of Microbiology, Czech Academy of Sciences, Vídeňská 1083, 142 00 Prague, Czech Republic; § Institute of Experimental Medicine, Czech Academy of Sciences, Vídeňská 1083, 142 00 Prague, Czech Republic; ∥ Department of Genetics and Microbiology, Faculty of Science, Charles University, Viničná 5, 128 43 Prague 2, Czech Republic; ⊥ Institute of Organic Chemistry and Biochemistry, Czech Academy of Sciences, Flemingovo nám. 2, 166 10 Prague, Czech Republic

## Abstract

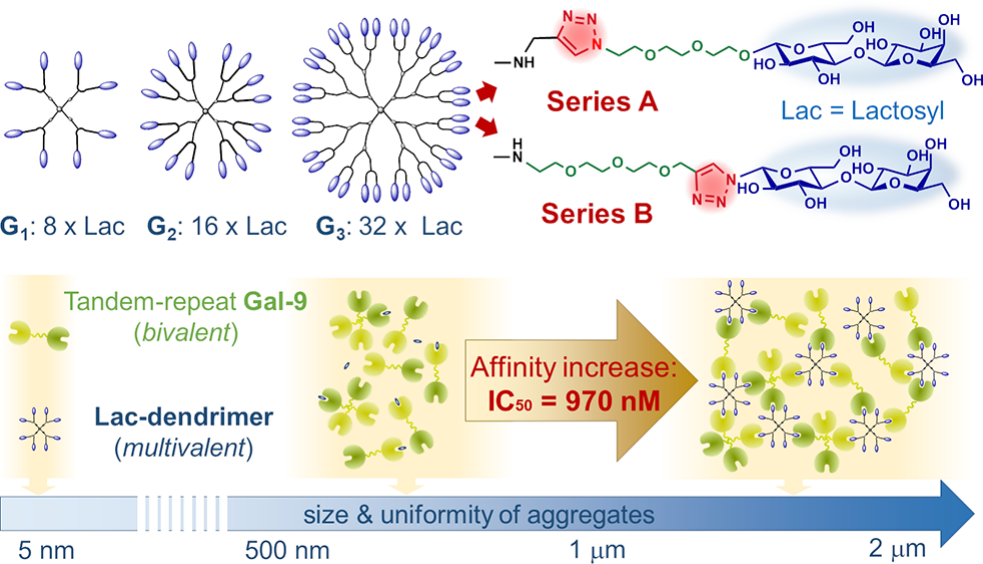

Galectins, the glycan binding proteins, and their respective carbohydrate ligands represent a unique fundamental regulatory network modulating a plethora of biological processes. The advances in galectin-targeted therapy must be based on a deep understanding of the mechanism of ligand–protein recognition. Carbosilane dendrimers, the well-defined and finely tunable nanoscaffolds with low toxicity, are promising for multivalent carbohydrate ligand presentation to target galectin receptors. The study discloses a synthetic method for two types of lactose-functionalized carbosilane glycodendrimers (Lac-CS-DDMs). Furthermore, we report their outstanding, dendritic effect-driven affinity to tandem-type galectins, especially Gal-9. In the enzyme-linked immunosorbent assay, the affinity of the third-generation multivalent dendritic ligand bearing 32 lactose units to Gal-9 reached nanomolar values (IC_50_ = 970 nM), being a 1400-fold more effective inhibitor than monovalent lactose for this protein. This demonstrates a game-changing impact of multivalent presentation on the inhibitory effect of a ligand as simple as lactose. Moreover, using DLS hydrodynamic diameter measurements, we correlated the increased affinity of the glycodendrimer ligands to Gal-3 and Gal-8 but especially to Gal-9 with the formation of relatively uniform and stable galectin/Lac-CS-DDM aggregates.

## 1 Introduction

Selective glycan-carbohydrate binding protein (lectin) recognition is a universal strategy of interaction and communication in living organisms.^1^ The β-galactoside-binding lectins (galectins; Gal-) play a key role in many crucial physiological processes,^2−4^ such as inter- and intra-cellular interaction, cell migration and adhesion,^5,6^ cellular signaling,^7^ apoptosis,^8^ or pre-mRNA splicing.^9^ Moreover, an abnormal expression of a particular galectin has been associated with various pathologies, including cancer, fibrosis, and heart diseases.^10−12^ Therefore, an in-depth understanding of the fundamental roles of galectins, particularly the sophisticated and selective galectin–ligand recognition, is vital for regulating and modulating both physiological and pathological processes.^13^ Recent advances in the field allowed for a rational design of highly selective ligands/inhibitors of particular galectins, demonstrating the high potential of galectin-targeted drug design.^4,14^

To date, 12 members of the human galectin family have been described: Gal-1, Gal-2, Gal-3, Gal-4, Gal-7, Gal-8, Gal-9, Gal-10, Gal-12, Gal-13, Gal-14, and Gal-16.^15,16^ From the structural point of view, the galectins are classified as prototype (e.g., homodimeric Gal-1), tandem repeat (e.g., bivalent Gal-8 and Gal-9), and chimeric type (Gal-3).^17^ Compared to Gal-1 and Gal-3, there has been much less investigation into the processes mediated by tandem-repeat galectins, such as Gal-8 and Gal-9. Generally, tandem-repeat galectins are challenging to study due to their complex structure encompassing two distinct carbohydrate recognition domains (CRD) interconnected with a peptide linker.^18^ Though basically highly conserved, the particular features in the structure of CRD of each galectin are responsible for the selectivity of their carbohydrate ligands. Numerous factors determine the extraordinary selectivity of the glycan–galectin recognition,^13^ even if not all of them are fully understood. A structural comparison in the binding between Gal-1, Gal-3, and Gal-9 CRDs and poly-LacNAc oligosaccharides is contained in a study by Nagae and Yamaguchi.^19^ It showed similarities between the interactions of Gal-3 and Gal-9N. In Gal-8N, a specific binding-site residue is Arg59 for recognition of sialylated and sulfated oligosaccharides, which is absent in Gal-9.^20^ Gal-1 compared to Gal-3 also contains a specific residue, His52, interacting with ligands in subsites C and D; in contrast to Gal-3, Gal-1 is well known to be acting as an exo-type lectin for longer oligosaccharide ligands,^21^ recognizing only the terminal disaccharide. The systematic development of small-molecule glycomimetic ligands revealed the influence of configuration and substitution at individual positions of the carbohydrate, e.g., an aromatic substitution at the C-3 position, on the potency and selectivity of the ligand.^22−24^ Substantial differences in the binding modes are also found in the interactions with the less defined subsite E, localized at the reducing end of carbohydrate ligands.^25,26^ The multivalent presentation of glycan ligands in macromolecules represents another natural strategy to ensure effective ligand–protein interaction. In multivalent ligand molecules, the overall binding potency can exceed the sole sum of monovalent ligand affinities by orders of magnitude.^27,28^ Moreover, unlike monovalent systems, the multivalent glycoconjugates can form unique two- and three-dimensional lattices with galectin receptors. There are clear differences between Gal-1 and Gal-3 in the recognition of multivalent ligands.^23,29,30^ For tandem-repeat galectins, the available systematic affinity data are rather limited.^31^ Therefore, despite potential therapeutical promises, the design of selective ligands/inhibitors of Gal-8 and Gal-9 remains to a large extent “*terra incognita*”. Recent studies have associated such multi-dimensional structures with significant biological activity,^32^ including induction of apoptosis in activated human T cells^33,34^ or negative regulation of neuroblastoma cell growth.^35^ Bertozzi et al. provided direct evidence of galectin ability to cross-link glycoligands on the cell surface.^36^ In the last few years, various multivalent carbohydrate-based or carbohydrate-decorated materials have been designed.^37,38^ Many types of scaffolds have been used for the multivalent presentation, such as polymers,^30,39^ calixarenes,^31,40^ serum albumins,^23,41,42^ peptides,^29,43,44^ or dendrimers.^45−47^

Dendrimers (DDMs) are regular, highly branched spherical macromolecules prepared by repetition of individual synthetic steps.^48,49^ As such, dendritic structures provide control over the physicochemical properties and valency of the system. Unlike some other synthetic platforms for multivalent presentation,^40,50^ dendritic structures allow fine-tuning of the attributes of the multivalent system by controlling the size, branching level, peripheral derivatization, spatial distribution, and density of the ligands. Thanks to their versatility, glycodendrimers (glyco-DDMs) are extensively exploited to study ligand–galectin interactions. In their pioneer work, André et al. demonstrated the different inhibitory potency of poly(amidoamine) (PAMAM) DDMs bearing aromatic *p*-isothiocyanatophenyl β-d-lactoside ligands (up to generation five) for Gal-1 and Gal-3.^51^ Cousin and Cloninger used lactose (Lac)-functionalized PAMAMs to investigate the multivalent Gal-1-mediated interactions. Remarkably homogeneous clusters were formed in the case of a significant excess of Gal-1 over glyco-DDMs. Another study reported that the glyco-DDMs inhibited Gal-1-moderated cellular aggregation of prostate carcinoma cells by providing a competitive binding site.^52^

A different density and spatial distribution of multivalently presented ligands can be responsible for the differences in the behavior of supramolecular assemblies of DDM-galectin complexes. Thus, the structural parameter variations may serve for modulating the inter- and intra-cellular processes. *N*-Acetyllactosamine (LacNAc)-functionalized PAMAMs synthesized by a chemoenzymatic method either inhibited or enhanced Gal-3-mediated cancer cell aggregation depending on the DDM structure and generation.^46^ An optimal topological distribution and density of surface glycans were studied on fully programmable supramolecular assemblies of Lac-decorated amphiphilic Janus DDMs known as glycodendrimersomes (GDSs). Agglutination assays of Gal-1^53^ and Gal-8^54^ with Lac-presenting GDSs suggested the optimal ligand topology and density, the increase in which did not lead to higher galectin reactivity.

Gal-1 and Gal-3 are the most studied representatives of the galectin family due to their biological significance and high abundance. On the other hand, only limited data are available for other galectins.^55^ Still, we expect that new findings concerning other less common galectins can contribute to a deeper understanding of the related processes in living cells. To our best knowledge, no study has been published to date on the interactions between Gal-9 and DDMs or, importantly, any other synthetic multivalent systems.

As recently demonstrated,^56^ the activity of functionalized dendrimers depends not only on peripheral derivatization but also on the structure of the dendritic scaffold. Carbosilane (CS) based CS-DDMs represent a well-established material with many benefits in the biomedical field. Yet, CS-DDMs have not been investigated as multivalent platforms to display galectin-binding ligands. Therefore, we present the very first study revealing the potential of glyco-CS-DDMs for galectin-targeted research. Two series of Lac-decorated carbosilane dendrimers (Lac-CS-DDMs) of the first to third generation have been prepared for this study. Lactose ligands were grafted to the periphery of the alkyne-terminated CS-DDMs using copper-catalyzed azide–alkyne cycloaddition (CuAAC). The two Lac-CS-DDMs series differ in the triazole ring position, which is either separated from the carbohydrate ligand by a triethylene glycol (TEG) spacer (series A) or directly linked to the C-1 position of lactose via a triazole moiety (series B). We previously found that a triazole next to the carbohydrate unit may have a positive effect on the affinity, especially to Gal-3.^29,39^ The interactions of Lac-CS-DDMs with human galectins (Gal-1, Gal-3, Gal-8, and Gal-9) were thoroughly studied using two complementary methods: (i) competitive enzyme-linked immunosorbent assay (ELISA), which assessed the ligand inhibitory potency, and (ii) dynamic light scattering (DLS), which correlated the Lac-CS-DDM-galectin aggregation behavior with an increased affinity.

## 2 Materials and Methods

### 2.1 Synthesis and Characterization of Lac-CS-DDMs

The 3-iodopropyl-terminated (G_1_I–G_3_I) and propargyl-terminated (G_1_A–G_3_A) CS-DDMs and 5-(OMOM)isophtalic acid were prepared according to our previously reported method.^57^ The acetylated lactose ligands N_3_-TEG-AcLac and N_3_-AcLac were prepared adopting the reported procedure.^58^ Unless otherwise stated, d-lactose, 2-{2-[2-(2-propynyloxy)ethoxy]ethoxy}ethanamine, hydroxybenzotriazole (HOBt), *N*,*N*′-dicyclohexylcarbodiimide (DCC), *N*-methylmorpholine (NMM), dimethylformamide (DMF), potassium carbonate, CuI, *N*,*N*-diisopropylethylamine (DIPEA), triethylamine, Dowex 50 WX8, Amberlite IRA402, and Chelex 100 (sodium form) were purchased from commercial sources and used without further purification.

NMR spectra were measured on a Bruker Avance 400 (^1^H at 400.1 MHz; ^13^C {1H} at 100.6 MHz; ^29^Si {^1^H} (INEPT technique) at 79.5 MHz) at 25 °C. ^1^H and ^13^C NMR signals of the prepared compounds were assigned to corresponding atoms utilizing gHSQC, gCOSY, gHMBC and HSQC TOCSY 2D NMR correlation spectra. ^1^H and ^13^C chemical shifts (δ/ppm) are given relative to residual solvent signals (δ_H_/δ_C_: DMSO-*d*_6_ 2.50/39.52; CDCl3 7.26/71.16); ^29^Si spectra were referenced to external standard hexamethyldisilane (−19.87 ppm). MALDI-TOF spectra were measured on an UltrafleXtreme MALDI-TOF/TOF mass spectrometer (Bruker Daltonics, Germany) with a 1 kHz smartbeam II laser. The measurements of G_1_-DDMs were done in the positive reflectron mode technique, with the mass range of 2–6 kDa. The measurements of G_2_-DDMs were done in the positive/negative linear mode technique, with the mass range of 2–20 kDa. The accelerating voltage was set at 25 kV. Typically, spectra were obtained by accumulating 3000 shots. Dihydroxybenzoic acid was used as the matrix (10 mg/mL in acetonitrile/0.1% TFA 1:1). Gel permeation chromatography (GPC) was performed on a Dionex UltiMate 3000 HPLC system equipped with a Phenogel 10E3Å column (Phenomenex), with 100% methanol as the mobile phase and diode array detector. Chromatograms were acquired at λ = 225 nm. Fourier transform infrared spectroscopy (FTIR) was carried out using a Nicolet 6700 with a mid-IR DTGS detector. The spectra were recorded in the range of 650–4000 cm^–1^ at a resolution of 4 cm^–1^ with the ATR technique (Zn/Se crystal).

Herein below, G_*n*_ refers to the DDM generation (*n* = 1, 2, and 3) and *m* is the number of isophthalic moieties in the DDM outer layer, which implies that the number of peripheral Lac units in Lac-CS-DDMs is 2*m*.

#### 2.1.1 Alkyne-Terminated Dendrimers G_*n*_-B_2*m*_ (**6**–**8**): General Synthetic Procedure

Compound **2** (1.1 equiv) and dry K_2_CO_3_ (1.3 equiv) were suspended in dry DMF. The iodopropyl-terminated DDM (G_1_I–G_3_I; 1 equiv; reactions were carried out in the 0.2–0.6 mM scale) was dissolved in petroleum ether (35–60 °C fraction) and added dropwise to the suspension. The reaction mixture was stirred overnight at 80 °C. Then, after evaporating DMF under vacuum, the crude product was dissolved in methanol and filtered through a short silica-gel column. The organic phase was evaporated (to ca. 3 mL volume) and purified using organic solvent nanofiltration (OSN). After evaporation to dryness, the products G_*n*_-B_2*m*_ (**6–8**) were obtained as brownish waxy solids (91–93% yield; analytical data of compounds are given in the Supporting Information).

#### 2.1.2 Peripheral Attachment of Lac Moieties via CuAAC Click Reaction: General Synthetic Procedure

The alkyne-terminated DDMs (G_*n*_-A_2*m*_ (**3**–**5**) or G_*n*_-B_2*m*_ (**6**–**8**); 1 equiv; reactions were carried out in a 0.1–0.3 mM scale) and azide-functionalized carbohydrates (N_3_-TEG-AcLac or N_3_-AcLac; 1.1 equiv per branch) were dissolved in dry DMF. Then, CuI (0.01 equiv per branch) and DIPEA (5 drops) were added. The suspension was transferred to a microwave reaction vial (10 mL), sealed with a septum, placed into the microwave reactor cavity, and irradiated up to 80 °C with stirring (600 rpm) for 3 h. After cooling to ambient temperature, the vial content was concentrated under reduced pressure. The solid was dissolved in methanol. Then, Chelex 100 was added to remove the Cu residues. After OSN, the products G_*n*_-A-AcLac_2*m*_ (**12a**–**14a**) and G_*n*_-B-AcLac_2*m*_ (**15a**–**17a**) were obtained as off-white powders (85–90% yield). The DDMs with peracetylated glycounits G_*n*_-A-AcLac_2*m*_ (**12a**–**14a**) or G_*n*_-B-AcLac_2*m*_ (**15a**–**17a**) (0.3–0.1 mM scale) were deacetylated under microwave irradiation (55 °C) according to the previously reported procedure.^57^ The completion of the deacetylation was checked using ^1^H NMR, and the deacetylation was repeated when necessary. The deacetylated glyco-DDMs were dissolved in distilled water and freeze-dried overnight to obtain products G_*n*_-A-Lac_2m_ (**12b**–**14b**) and G_*n*_-B-Lac_2*m*_ (**15b**–**17b**) as puffy off-white powders (97–99% yield; analytical data of compounds are given in the Supporting Information). The deacetylated glyco-DDMs were checked for the presence of Cu ions by inductively coupled plasma–optical emission spectroscopy (ICP-OES). Products of insufficient purity were subsequently dissolved in water and treated with Chelex 100 (sodium form) until the complete removal of copper was achieved according to ICP-OES (detection limit 1–5 ppm Cu).

#### 2.1.3 OSN System

Nanofiltration^59^ was carried out using solvent-resistant stirred cell Millipore (for 47 mm membranes) equipped with 1 or 3 kDa MWCO regenerated cellulose ultrafiltration discs Ultracel (Millipore) and PTFE encapsulated O-rings Teflex (Eriks, FEP/Viton), with nitrogen as a driving gas (transmembrane pressure 5 bar). Crude dendritic products were dissolved in 50 mL of an appropriate solvent (typically MeOH or MeOH/DCM mixtures up to a 1:1 ratio), and the solution was filtered through the membrane until a residual volume of 5 mL of the retentate was reached. The retentate was then diluted with 45 mL of the same solvent mixture and filtered. ^1^H NMR was used to monitor the purification progress. The procedure was repeated as necessary; three to four cycles were typically sufficient to obtain analytically pure products.^60^

### 2.2 Production of Human Galectins

Recombinant human galectins Gal-1, Gal-3, Gal-8, and Gal-9 were produced as N-terminal His-tagged constructs cloned in a pET-Duet1 vector (restriction sites *Nco*I/*Asc*I for Gal-1 and Gal-3 and *Asc*I/*Not*I for Gal-8 and Gal-9). The gene construct of Gal-1 contains a mutation C2S, which increases its stability and renders it resistant to oxidation.^61,62^ The plasmids carrying the gene constructs of Gal-1 and Gal-3 were prepared as described previously.^22^ The plasmid containing the gene construct of Gal-8 was a kind gift from Prof. L. Elling, RWTH Aachen, Germany; it contains a full-length peptide linker of 34 aa (3717 Da). The gene construct of Gal-9 was designed according to the study by Itoh et al.^63^ where they found the most stable and soluble form of Gal-9 (with a truncated mutated peptide linker of **HPPY**PMPF, 985 Da). The gene was prepared commercially (Generay Biotech. Co, Shanghai, China). Galectins were produced and purified as described previously.^22,64^ Briefly, the transformed *Escherichia coli* Rosetta 2(DE3)pLysS competent cells were inoculated into a Luria-Bertani medium (LB; 60 mL, 10 g/L tryptone, 5 g/L NaCl, and 5 g/L yeast extract) and cultivated at 37 °C and 220 rpm overnight. The precultures were inoculated into a Terrific Broth medium (TB; 600 mL; 12 g/L tryptone, 24 g/L yeast extract, 4 mL/L glycerol, 2.31 g/L KH_2_PO_4_, and 12.54 g/L K_2_HPO_4_) and cultivated at 37 °C and 150 rpm. The LB and TB media contained ampicillin (100 μg/mL) and chloramphenicol (34 μg/mL). The protein expression was induced by adding 0.5 mM isopropyl 1-thio-β-d-galactoside (IPTG) when the culture grew to an optical density (OD_600_) of 0.6–0.8. Then, the cells were cultivated at 25 °C for 24 h and harvested by centrifugation (8880*g*, 20 min, 4 °C).

For the purification of galectins, harvested cells were suspended in an equilibration buffer (20 mM phosphate/500 mM NaCl/20 mM imidazole, pH 7.4). Phenylmethylsulfonyl fluoride (PMSF, 1%) was added to prevent cleavage by proteases. The suspension was sonicated using an UltraSonic Processor UP50 H (Ultrasound Technologies, Caldicot, UK) for six cycles (1 min pulse, 2 min break on ice). After centrifugation (20,230*g*, 20 min, 4 °C), the cell-free extract was loaded on an equilibrated Ni-NTA column (GE Medical Systems, Prague, Czech Republic). First, the column was washed with equilibration buffer, then with equilibration buffer containing 0.5% Triton X-100 (10- to 20-fold column volume) to remove traces of lipopolysaccharide.^64^ Then, the column was rewashed with pure equilibration buffer. Bound galectins were eluted with an elution buffer containing 500 mM of imidazole. In the case of Gal-9, a pre-elution step of washing with 50 mL of 50 mM imidazole was included to reduce non-specific protein adsorption to the column. Then, elution with a gradient of imidazole (100–500 mM) followed. Fractions were analyzed for protein content using the Bradford assay^65^ calibrated for immunoglobulin G (IgG), pooled, and dialyzed overnight in PBS buffer pH 7.5 (7 L) containing 2 mM EDTA followed by 4 h of dialysis in PBS buffer (7 L). Gal-1, Gal-3, Gal-8, and Gal-9 proteins were stable at 4 °C for approximately 2 months. The purity of prepared galectins was confirmed on SDS-PAGE (12% gel; Supporting Information, Figure S66).

### 2.3 Competitive ELISA Assay

The inhibitory potential of prepared Lac-CS-DDMs toward Gal-1, Gal-3, Gal-8, and Gal-9 was determined using a competitive ELISA assay with immobilized asialofetuin (ASF; a glycoprotein terminated with LacNAc moieties).^41^ ASF (0.1 μM in PBS, 50 μL/well) was coated in microtiter plates (Nunc Immuno Sorb, Thermo Fisher Scientific, USA) and incubated overnight. Then, the wells were washed with 3 × 250 μL of PBS buffer containing 0.05% Tween; this washing step followed each incubation step. The wells were then blocked with 2 mg/mL BSA in PBS buffer (250 μL/well) and incubated for 1 h. After washing, serial dilutions of the ligands (Lac-CS-DDMs or lactose standard) in EPBS (2 mM EDTA in PBS) buffer with the addition of DMSO for better solubility (10% *v*/*v*) were incubated with Gal-1, Gal-3, Gal-8, or Gal-9 (2.5 μM for Gal-1 and Gal-3 and 1 μM for Gal-8 and Gal-9, final concentration, 2 h). For the direct binding ELISA assay, the incubation step comprised serial dilution of the respective galectin in EPBS (50 μL/well). After a washing step, the anti-His-antibody conjugated to horseradish peroxidase (Santa Cruz, 1:1000 dilution in PBS for Gal-1 and Gal-3 and 1:2000 dilution in PBS for Gal-8 and Gal-9) was used for labeling of residual bound galectin and subsequent colorimetric detection. After another washing step, the substrate for the peroxidase reaction TMB One (Kem-En-Tech, Denmark; 50 μL/well) was added and incubated the reactions until visible blue staining appeared (3–20 min). The reaction was stopped by adding 3 M HCl (50 μL per well), accompanied by a color shift to yellow as determined spectrophotometrically by an absorbance microplate reader (Sunrise Tecan Group Ltd., CH). The intensity of the signal at 450 nm corresponded to the amount of galectin bound to the wells. A background value, as a mean of the negative control wells containing buffer instead of galectin/ligand, with an absorbance of ca. 0.05–0.075 was subtracted from the measured absorbances. The values of half maximal inhibitory constants (IC_50_) were calculated from the non-linear regression (dose–response inhibition-variable slope) of the sigmoidal curves using GraphPad Prism 7 (GraphPad Software, USA) from at least three independent experiments with at least two different galectin batches. Using the standard inhibitor lactose, it was verified that the impact of the DMSO co-solvent concentration (0–10% *v*/*v*) on the galectin affinity was not significant.

### 2.4 DLS Measurements

The hydrodynamic diameter of the nanoparticles of galectins, Lac-CS-DDMs, and their aggregates was measured by dynamic light scattering (DLS) using a Zetasizer Nano ZS (Malvern Instruments Ltd., UK) at 25 °C in Malvern disposable plastic microcuvettes (100 μL sample volume). The light scattered at 173° from the incident light was fitted to an autocorrelation function using the method of cumulants (Malvern Instruments Ltd., UK). Commercial sterile PBS was used for sample preparation. Components were (i) dissolved in PBS to obtain a 10 μM final concentration (galectins, Lac-CS-DDMs) or (ii) mixed in 150:1, 6:1, and 2:1 ratios (galectin/G_*n*_-A-Lac_2*m*_ and galectin/lactose) to obtain a final galectin concentration of 10 μM. Mixtures of components were incubated for 90 min at room temperature. The samples were vortexed prior to measurements. The hydrodynamic diameters were determined from five independent repetitions (each 10–50 runs). Multimodal intensity-weighted particle size distribution was used for data analysis.

## 3 Results and Discussion

### 3.1 Synthesis and Characterization of Lac-CS-DDMs

**Scheme 1:**
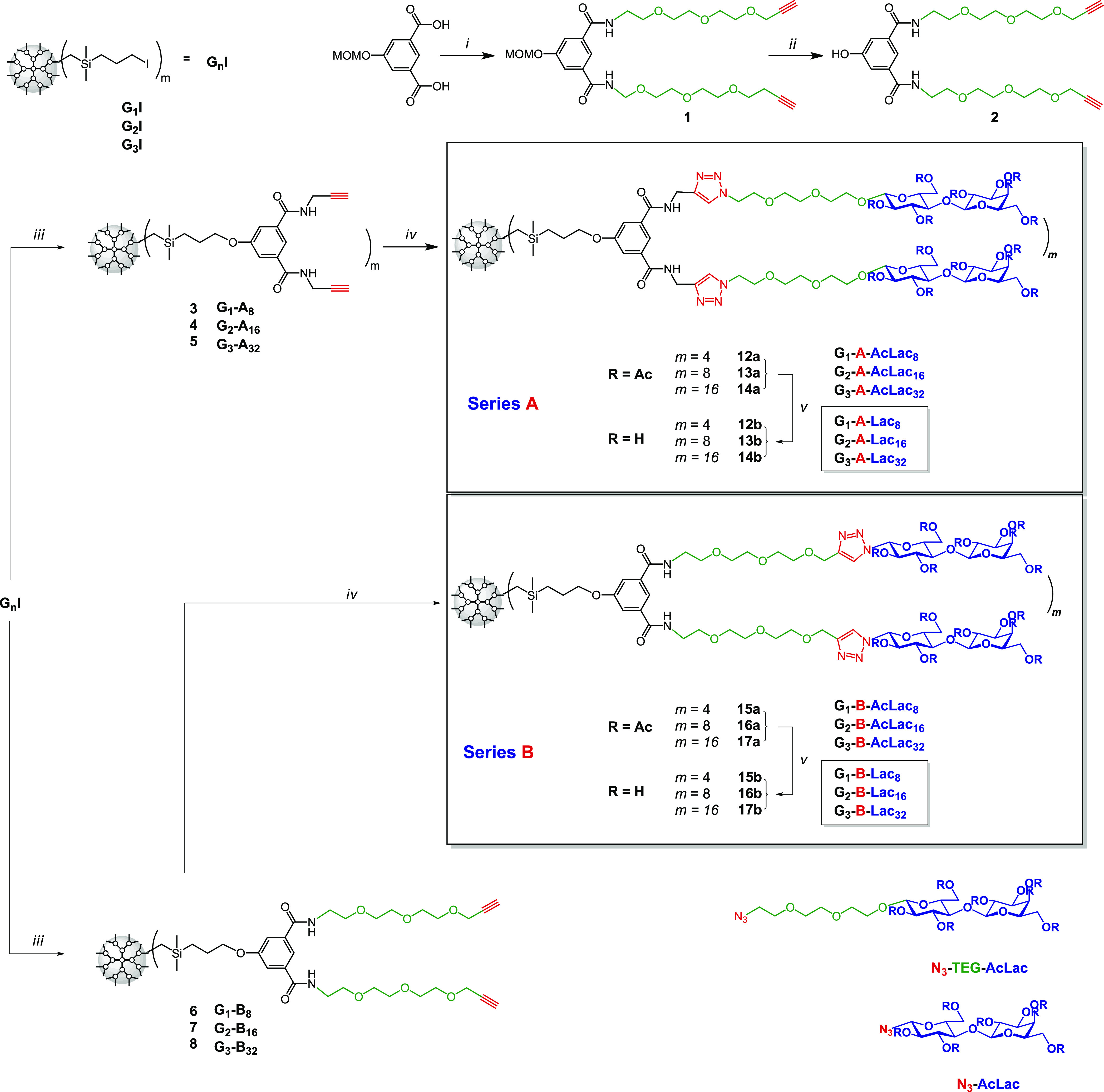
Synthetic Route toward a Series of Lac-CS-DDMs for the Multivalent Presentation of Lac (i) 2-{2-[2-(2-Propynyloxy)ethoxy]ethoxy}ethanamine, EDC·HCl, HOBt, NMM, DMF, rt, (ii) Dowex H^+^, (iii) for series G_*n*_-A_2*m*_, see ref (57); for series G_*n*_-B_2*m*_: K_2_CO_3_, **2**, DMF, 80 °C, (iv) CuI, DIPEA, DMF, MW, 80 °C, N_3_-TEG-AcLac for series A, N_3_-AcLac for series B(v) Et_3_N, MeOH, water, MW. For complete Lac-CS-DDM structures, see Figure S1.

Lactose-functionalized Lac-CS-DDMs of series A (G_*n*_-A-Lac_2*m*_) were prepared according to a slightly modified procedure from polyalkyne substrates G_*n*_-A_2*m*_, which we previously developed for the preparation of glucose- and galactose-decorated CS-DDMs.^57^ Analogical series B was synthesized to investigate the effect of the triazole ring position on the overall avidity of the multivalent dendritic system. First, the phenolic alkyne derivative with propargyl-terminated TEG chains **2** was prepared in two steps. A new type of alkyne-terminated CS-DDMs (G_*n*_-B_2*m*_) was synthesized from dendritic precursors G_1_I–G_3_I by nucleophilic substitution of iodine by a phenolic group of the substrate **2** in the presence of a mild base (Scheme 1). The shift of the triplet signal from 3.18 (−CH_2_I) to 3.85–3.98 ppm (−CH_2_O−) in ^1^H NMR spectra indicated the completion of the reaction. The two series of alkyne-terminated CS-DDMs were used for the attachment of sugar moieties, combining alkyne-terminated series (G_*n*_-A_2*m*_) with azide-TEG-functionalized lactose and TEG-alkyne-terminated series (G_*n*_-B_2*m*_) with azide-functionalized lactose.

Acetylated Lac-decorated CS-DDMs **12a**–**14a** and **15a**–**17a** were prepared following the previously reported robust synthetic protocol.^57^ This method facilitates peripheral attachment of carbohydrate moieties in a multivalent manner via copper(I)-catalyzed azide–alkyne cycloaddition (CuAAC) (Scheme 1). Nevertheless, in this synthetic step, we replaced the reaction setup based on the original Sharpless conditions (CuSO_4_ and sodium ascorbate as a reducing agent)^66^ by a procedure facilitating triazole ring formation under microwave irradiation and elevated temperature (CuI, DIPEA, MW, 80 °C).^67^ We have found that this method provides quantitative conversion in a shorter reaction time. Moreover, the yields of the acetylated Lac-CS-DDMs (G_*n*_-A-AcLac_2*m*_ and G_*n*_-B-AcLac_2*m*_) were generally higher due to the absence of aqueous work-up. The microwave-assisted reaction setup is known to accelerate the reaction progress^68,69^ and to be generally beneficial for synthesizing glyco-CS-DDMs.

Then, we took advantage of a substantial size difference between the reaction components and purified the G_*n*_-A-AcLac_2*m*_ and G_*n*_-B-AcLac_2*m*_ by OSN.^59,60^ For the final *O*-deacetylation step, we used triethylamine-catalyzed deacetylation under microwave irradiation^70^ as this method accelerates the deprotection process. The disappearance of seven acetyl group singlets around 2.0 ppm in ^1^H NMR indicated efficient deacetylation. Finally, the formation of the Lac-CS-DDMs differing in the triazole ring position G_*n*_-A-Lac_2*m*_ and G_*n*_-B-Lac_2*m*_ was confirmed by one- and two-dimensional NMR (Figures S2–S60) and for lower generations of Lac-CS-DDMs also by MALDI-TOF MS analyses (Figures S61–S63).

Even though series A and B differ only in the triazole ring position, we observed subtle differences in NMR spectra demonstrating the structural features of both series. In Lac-CS-DDM series A, the characteristic ^13^C signals of the skeletal C-1 and C-1′ carbons are typically positioned at 104–102 ppm as both C-1 and C-1′ carbons form *O*-glycosidic bonds. Accordingly, the respective signals of H-1 and H-1′ are located at 4.22–4.20 ppm. On the contrary, in series B, the *N*-glycosidic bond to the triazole ring formed as a result of the CuAAC shifts the C-1 carbon upfield (87–86 ppm) compared to the corresponding C-1′ signal (104–99 ppm). Similarly, respective H-1 signals are positioned upfield (4.8–4.3 ppm) compared to H-1′ signals (5.6–5.1 ppm).

In addition, the triazole ring position was identified by two-dimensional NMR experiments. The H-C correlated gHMBC spectra of the Lac-CS-DDMs of series A showed an interaction between (i) the end CH_2_ group of the TEG chain and the skeletal H-1 proton of Lac and (ii) CH group of the triazole ring, CH_2_ group of TEG, and amidic NH protons. On the contrary, in series B, the CH triazole signal interacts with the H-1 skeletal proton of Lac and both carbons of the triazole ring interact with CH_2_ protons of TEG (Figures S7, S14, S21, S45, S52, and S59).

### 3.2 Affinity of Glycodendrimers to Galectins (ELISA)

To determine the affinity of prepared Lac-CS-DDMs to a representative selection of galectins, we produced prototype Gal-1, chimera-type Gal-3, and tandem-repeat Gal-8 and Gal-9 as His-tagged constructs^31,64,70^ in *E. coli* Rosetta 2(DE3)pLysS. We purified them by Ni-NTA affinity chromatography with elution by imidazole. The binding affinity of prepared galectins to ASF was determined by the direct ELISA assay; respective *K*_D_ values: *K*_D_ = 4.4 μM for Gal-1, *K*_D_ = 3.2 μM for Gal-3, *K*_D_ = 0.37 μM for Gal-8, and *K*_D_ = 0.30 μM for Gal-9 (curves shown in Figure S67).

**Figure 1:**
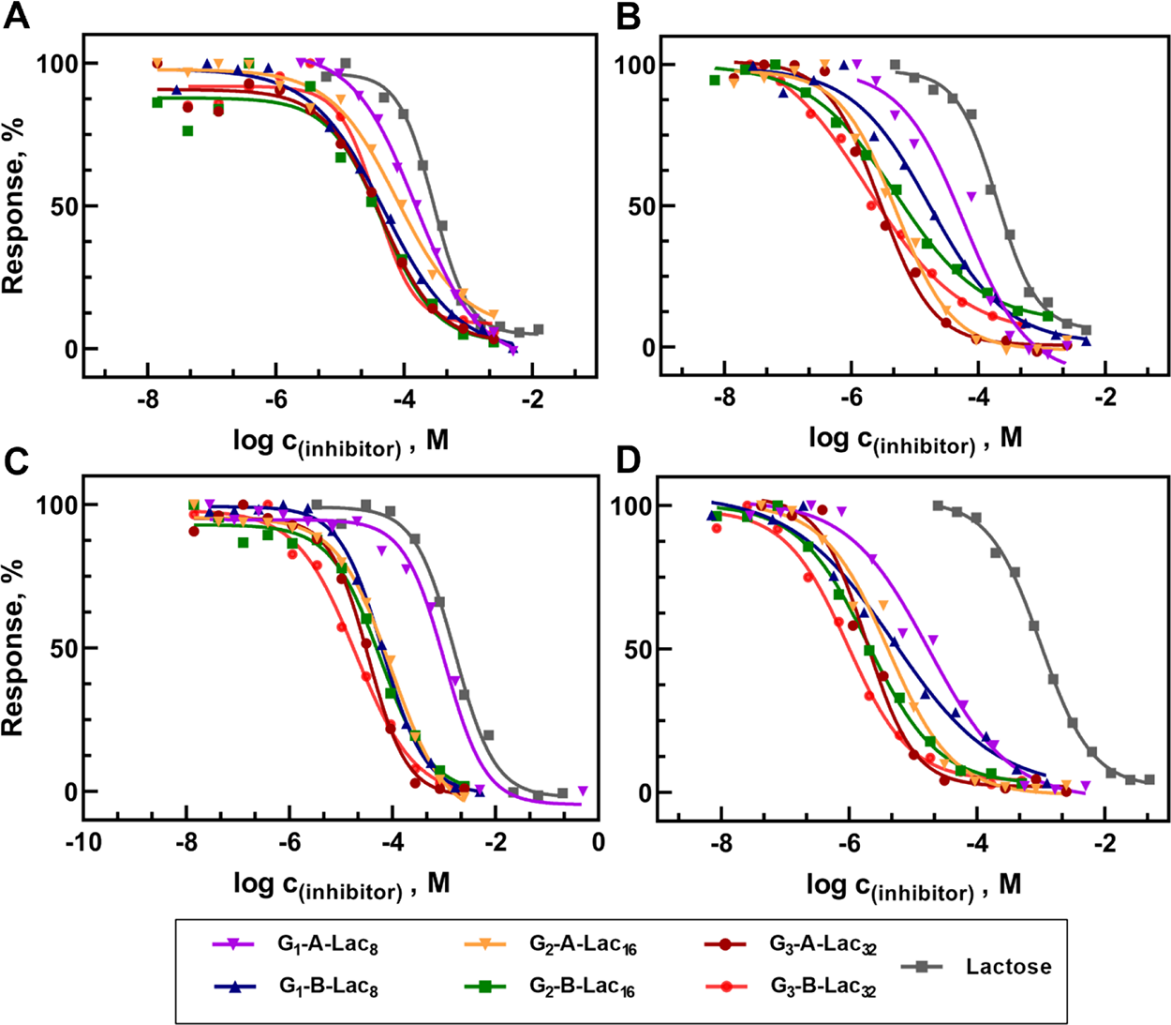
Competitive binding inhibition of galectins (A, Gal-1, B, Gal-3, C, Gal-8, and D, Gal-9) to ASF by Lac-CS-DDMs determined by ELISA. For respective IC_50_ values, see Table 1. Used concentrations of galectins: 2.5 μM for Gal-1 and Gal-3 and 1 μM for Gal-8 and Gal-9. The curve of the dose-dependent binding inhibition of G_1_-A-Lac_8_ to Gal-8 (panel C, purple) was not fully saturated due to a very low affinity of G_1_-A-Lac_8_. Therefore, the curve was extrapolated and estimation of IC_50_ was employed. The 100% response (top plateau of the sigmoidal curve) corresponded to low ligand concentrations—all galectin was bound to the wells; 0% response (bottom plateau) corresponded to high ligand concentrations—all galectin was inhibited, and none bound to the wells.

To determine the inhibitory potency of prepared Lac-CS-DDMs toward galectins, we employed competitive ELISA.^41^ Varying concentrations of glycodendrimer inhibitors G_*n*_-A-Lac_2*m*_ and G_*n*_-B-Lac_2*m*_ competed for binding galectins in solution with immobilized competitor ASF, which interacts with galectins. The amount of residual galectin bound to immobilized ASF was determined spectrophotometrically using the anti-His-tag antibody conjugated to horseradish peroxidase. The inhibitory potency of prepared Lac-CS-DDMs was compared to monovalent lactose as a standard, and a relative potency (*rp*) for each galectin was calculated (Table 1 and Figure 1). To further demonstrate the efficiency of multivalent presentation, the relative potency per lactosyl (*rp*/*lac*) was also determined (Supporting Information, Table S1). This value represents the positive cooperativity and, hence, the affinity increase for lactose bound in a multivalent system (*rp*/*lac* > 1) compared to the free lactose. Since some compounds were poorly water-soluble, the dimethyl sulfoxide (DMSO) co-solvent (2–10% *v*/*v*) was applied to reach the saturating concentration in the dose–response inhibition curves.

**Table 1:** Inhibitory Potency of Glycodendrimers toward Gal-1, Gal-3, Gal-8, and Gal-9

		IC_50_ [μM]							
sample	valency	Gal-1	*rp*^a^	Gal-3	*rp*^a^	Gal-8	*rp*^a^	Gal-9	*rp*^a^
lactose	1	310 ± 38	1.0	116 ± 26	1.0	1680 ± 420	1.0	1350 ± 440	1.0
G_1_-A-Lac_8_	8	150 ± 23	2.1	93 ± 24^ns^	1.2	ca. 920^b^	1.8	33 ± 16	41
G_2_-A-Lac_16_	16	69.4 ± 8.8	4.4	3.63 ± 0.92	37	100 ± 17	17	4.66 ± 0.52	290
G_3_-A-Lac_32_	32	44.6 ± 1.6	6.9	5.0 ± 2.2	23	69 ± 26	25	2.4 ± 1.5	560
G_1_-B-Lac_8_	8	51 ± 19	6.0	12.1 ± 7.1	9.6	50 ± 31	34	16.6 ± 5.2	80
G_2_-B-Lac_16_	16	49 ± 15	6.2	5.48 ± 0.85	25	52 ± 17	32	2.45 ± 0.62	550
G_3_-B-Lac_32_	32	20.3 ± 8.6	13	2.93 ± 0.67	47	21.7 ± 2.1	77	0.97 ± 0.57	1390

a Relative inhibitory potency (*rp*) is a ratio of the inhibitory potency of lactose and the respective glycodendrimer, i.e., *rp* = IC_50_ (lactose)/IC_50_ (glycodendrimer). All data were measured in a minimum of a triplicate. A background value (as a mean of the negative control wells containing buffer instead of galectin/ligand, with an absorbance of ca. 0.05–0.075) was subtracted from the measured absorbances. The values were rounded to two significant figures of the standard deviation.

b The value is an estimate from the extrapolation of the dose-dependent binding inhibition curve.

Overall, the more glycans on the dendrimer, the better the affinity to the galectins was obtained. The series A of Lac-CS-DDMs featuring a directly bound lactose showed a lower overall inhibitory potency than the series B with the triazole group adjacent to the glycan. This is in line with our previous studies where we found a considerable effect of the carbohydrate linker on the affinity to galectins—the direct *N*-triazole linker exhibited the best result with Gal-3 compared to the *O*-ethyltriazole and thiourea linker in a series of glycopolymers^39^ and partially also in the study with glycoclusters, where, however, this effect was not apparent with Gal-1.^29^ In our present study, the positive impact of the *N*-triazole linker adjacent to the carbohydrate moiety is evident for all galectin types, including tandem-repeat galectins. The *N*-triazole part interacts with the less conserved subsite E in the binding grove, which has so far been only marginally studied, especially in connection with glycomimetic thiodigalactoside inhibitors.^25,26^ There, arginine-π interactions have been deemed responsible for the affinity increase, particularly with Gal-3 (Arg186). Since conserved arginine residues are found in the subsite E of galectins (i.e., Arg74 for Gal-1, Arg254 for Gal-8C, Arg87 for Gal-9N, and Arg260 for Gal-9C), we hypothesize that this might be a particular conserved feature of the galectin CRD domain influencing linker binding to subsite E; however, this can only be reliably confirmed by a crystallography study. The best inhibitor of all galectins was G_3_-B-Lac_32_. Especially for Gal-9, it showed IC_50_ in nanomolar range (970 nM), 1400 times better than free lactose. Notably, only in the case of Gal-9, a strong positive cooperativity was observed between multivalent lactosyls (*rp*/*lac* was up to 44), in contrast to other tested galectins Gal-1, Gal-3, and Gal-8 (Supporting Information, Table S1). A partial explanation of the binding differences might already be found in the interaction of the binding sites of respective galectins with lactose (Supporting Information, Figure S68). However, the relative potencies presented in Table 1 and in the Supporting Information, Table S1 clearly show that the major contribution of these high avidities lies in the multivalency effect, especially for Gal-9. Tandem-repeat galectins (Gal-8 and Gal-9), due to the presence of two close covalently bound CRDs, have much higher prospects of taking advantage of, e.g., the statistical rebinding effect, than other galectin types.

### 3.3 Aggregation Behavior in Solution (DLS)

DLS experiments on the A series of Lac-CS-DDMs and G_3_-B-AcLac_32_ were performed to provide insight into the complexity of multivalent galectin/Lac-CS-DDM interactions (Table 2). Unlike ELISA assays that simulate competitive binding on the cell surface, DLS is a straightforward method to evaluate non-surface bound multivalent assemblies.^71^ Thus, it is interesting to relate the data obtained by these two methods. Since the transformation of intensity-weighted size distribution to volume-weighted size distribution requires information on the refractive index and absorption of the formed particles and assumes their homogeneity and spherical shape,^72^ all presented data come from the analysis of the multimodal intensity-weighted size distribution. As the scattering intensity correlates with the particle diameter to the sixth power, whereas the mass only to the third power, the abundance of larger particles is overestimated at the expense of the smaller ones with respect to the real sample composition, especially in the case of polydisperse samples with multiple size populations.^73^ This means that, due to the presence of large aggregates, the particles smaller by orders of magnitude present in the sample may not be detected. Despite the above bottlenecks, we believe that using the size-to-intensity plot is the most appropriate as it is highly sensitive to a starting aggregation, which may be undetectable in the size-to-volume distribution, and it is well suited to compare results between different galectins and Lac-CS-DDM generations to address general differences and trends in their aggregation behavior.

**Table 2:** Hydrodynamic Sizes (Diameters) of Particles in the Solutions of Dendrimers, Galectins, and Their Mixtures Measured by DLS^a^

			intensity-weighted size distribution^b^								
			small particles				aggregates				
			<50 nm				>0.1 μm		>0.5 μm		
entry	components	ratio	nm	%	nm	%	μm	%	μm	%	PI^c^
Galectins											
1	Gal-1		1.3 ± 0.1	47	19.0 ± 6.0	21			1.2 ± 0.5	32	0.4
2	Gal-3		7.9 ± 1.4	36			0.4 ± 0.1	40	1.1 ± 0.1	13	0.6
3	Gal-8		5.4 ± 0.5	20					1.4 ± 0.3	80	0.7
4	Gal-9		5.8 ± 0.1	26					0.5 ± 0.1	65	0.6
Lac–CS-DDMs											
5	G_1_-A-Lac_8_		12.1 ± 2.0	89							0.3
6	G_2_-A-Lac_16_		10.2 ± 0.4	75					0.6 ± 0.1	25	0.4
7	G_3_-A-Lac_32_		9.2 ± 0.7	27			0.1 ± 0.1	8	0.7 ± 0.3	66	0.8
8	G_3_-B-Lac_32_		9.2 ± 0.3	46			0.3 ± 0.1	54			0.8
Gal/Lac											
9	Gal-1/Lac	150:1	4.2 ± 2.9	7			0.4 ± 0.1	85			0.4
10		6:1							0.8 ± 0.2	98	0.7
11		2:1							0.5 ± 0.1	98	0.7
12	Gal-3/Lac	150:1	7.1 ± 0.4	21			0.2 ± 0.1	77			0.7
13		6:1	6.9 ± 0.1	23			0.2 ± 0.1	71			0.6
14		2:1					0.3 ± 0.1	92			0.5
15	Gal-8/Lac	150:1	3.5 ± 0.9	8					0.7 ± 0.1	92	0.7
16		6:1	5.5 ± 0.6	25					0.9 ± 0.2	74	0.6
17		2:1	4.1 ± 0.4	18					0.6 ± 0.1	82	0.6
18	Gal-9/Lac	150:1	5.3 ± 0.4	32					1.0 ± 0.2	68	0.8
19		6:1	3.6 ± 2.2	6					0.8 ± 0.2	94	0.6
20		2:1	2.0 ± 0.6	10					0.7 ± 0.2	90	0.7
Gal/Lac–CS-DDM											
21	Gal-1/G_1_-A-Lac_8_	150:1	1.4 ± 0.1	47	21.0 ± 2.0	11	0.2 ± 0.1	8	1.3 ± 0.2	26	0.5
22		6:1	0.9 ± 0.4	29	12.7 ± 1.4	33	0.3 ± 0.1	31			0.3
23		2:1	1.5 ± 0.2	6	11.2 ± 0.8	85					0.1
24	Gal-1/G_2_-A-Lac_16_	150:1	1.2 ± 0.2	52	21.0 ± 8.0	16			1.7 ± 0.2	32	0.4
25		6:1	0.9 ± 0.2	10	14.3 ± 1.2	74					0.4
26		2:1	9.7 ± 0.4	91							0.3
27	Gal-1/G_3_-A-Lac_32_	150:1	1.5 ± 0.7	59			0.3 ± 0.2	38			0.5
28		6:1	8.5 ± 0.3	33			0.4 ± 0.1	60			0.5
29		2:1	11.5 ± 0.7	65			0.4 ± 0.1	36			0.5
30	Gal-1/G_3_-B-Lac_32_	150:1	0.8 ± 0.1	2	7.4 ± 0.4	32	0.2 ± 0.1	60			0.2
31		6:1	7.3 ± 0.4	38			0.3 ± 0.1	62			0.3
32		2:1	9.3 ± 0.3	40			0.4 ± 0.1	60			0.8
33	Gal-3/G_1_-A-Lac_8_	150:1	6.0 ± 0.7	64			0.3 ± 0.1	28			0.4
34		6:1	6.6 ± 0.4	2					1.0 ± 0.1	97	0.5
35		2:1	7.5 ± 0.2	16			0.1 ± 0.1	18	0.5 ± 0.1	74	0.6
36	Gal-3/G_2_-A-Lac_16_	150:1	6.8 ± 0.1	19			0.4 ± 0.1	74			0.5
37		6:1							1.1 ± 0.3	100	0.2
38		2:1	5.8 ± 0.3	12					1.4 ± 0.4	88	0.5
39	Gal-3/G_3_-A-Lac_32_	150:1	7.3 ± 0.1	18					0.6 ± 0.2	77	0.5
40		6:1	8.7 ± 0.5	57			0.4 ± 0.1	36			0.4
41		2:1							1.1 ± 0.2	100	0.4
42	Gal-3/G_3_-B-Lac_32_	150:1	5.0 ± 0.6	10					0.5 ± 0.1	90	0.4
43		6:1					0.4 ± 0.1	99			0.2
44		2:1	10.6 ± 0.4	52			0.4 ± 0.1	48			0.7
45	Gal-8/G_1_-A-Lac_8_	150:1	6.6 ± 0.1	27					0.8 ± 0.2	73	0.6
46		6:1							0.7 ± 0.2	97	0.6
47		2:1							0.8 ± 0.2	97	0.5
48	Gal-8/G_2_-A-Lac_16_	150:1	4.7 ± 0.1	25			0.4 ± 0.1	75			0.5
49		6:1							0.6 ± 0.1	99	0.4
50		2:1							0.7 ± 0.3	74	0.7
51	Gal-8/G_3_-A-Lac_32_	150:1	5.5 ± 0.7	17					0.8 ± 0.1	82	0.7
52		6:1							1.0 ± 0.1	100	0.2
53		2:1	5.7 ± 0.7	10					1.2 ± 0.1	90	0.2
54	Gal-8/G_3_-B-Lac_32_	150:1							0.5 ± 0.1	100	0.5
55		6:1							0.5 ± 0.1	100	0.3
56		2:1	8.7 ± 1.3	19					0.5 ± 0.1	81	0.5
57	Gal-9/G_1_-A-Lac_8_	150:1					0.3 ± 0.1	5	1.2 ± 0.2	94	0.5
58		6:1							1.8 ± 0.2	89	0.5
59		2:1							1.6 ± 0.4	91	0.4
60	Gal-9/G_2_-A-Lac_16_	150:1							1.6 ± 0.4	100	0.5
61		6:1							1.4 ± 0.1	100	0.1
62		2:1							2.0 ± 0.2	90	0.4
63	Gal-9/G_3_-A-Lac_32_	150:1							1.9 ± 0.1	100	0.4
64		6:1							2.0 ± 0.2	88	0.4
65		2:1							1.8 ± 0.1	99	0.3
66	Gal-9/G_3_-B-Lac_32_	150:1							1.2 ± 0.1	100	0.3
67		6:1							1.0 ± 0.1	100	0.2
68		2:1							1.1 ± 0.1	100	0.5

a Galectin (10 μM) in PBS and 0.067, 1.67, or 5 μM dendrimers from the G_*n*_-A-Lac_2*m*_ series and the G_3_-B-Lac_32_ depending on the Gal/Lac-CS-DDM ratio (150:1, 6:1, and 2:1, respectively); 90 min incubation time.

b Fractions with the abundance under 2% are omitted.

c Polydispersity index.

Initial experiments at a 10 μM concentration revealed self-assembling tendencies of both Lac-CS-DDMs and galectins as individual components. The investigated galectins are known to self-associate into homodimers (prototype, Gal-1;^74^ tandem repeat, Gal-8 and Gal-9^13^) or oligomers (chimera type, Gal-3)^75^ via the non-lectin domain (hydrophobic) interactions. Other interactions may also govern the formation of more or less irregular aggregates depending on the conditions. For example, Miyanishi et al. discovered that human Gal-9 interacts with itself and other galectin family members via CRDs.^76^

Although all studied galectins showed some degree of aggregation, the population corresponding to monomers or dimers was observable in all cases. The size of pure Gal-1 (1.3 ± 0.1 nm, entry 1) is smaller than observed by Scott et al.;^77^ nevertheless, the conditions are not fully comparable. A reported radius of 1.9 nm was obtained in a much higher Gal-1 concentration, in different buffers and the presence of lactose. The size of particles we have observed in the presence of lactose (entry 9) is in good accordance with the reported value. Interestingly, most mixed Gal-1/DDM samples also display small particles around 1 nm, especially at higher Gal-1/Lac-CS-DDM ratios. Gal-3 is known to be monomeric in diluted solutions under 100 μM.^75^ The size of observed particles (7.9 ± 1.4 nm; entry 2) is in good accordance with the data reported for a full-length Gal-3 by Halimi et al.^78^ Surprisingly, size-related data for tandem galectins are lacking; however, if we consider the rather conserved CRD domain, then we can expect that the size of tandem galectins will be close to CRD domain dimers observed by Birdsall et al. (6 nm)^79^ or Gal-1 homodimers (reported radius of 2.55 nm, i.e., 5.1 nm diameter).^80^ Gal-8 and Gal-9 (ca. 36 kDa) showed higher tendency to form large aggregates during our measurements than Gal-1 or Gal-3. There, the diameter values of minor-intensity fractions in Gal-8 (5.4 ± 0.5 nm) and Gal-9 (5.8 ± 0.1 nm) samples indicate the presence of monomers and dimers. In the free Lac-CS-DDMs, the tendency to form large aggregates increased with increasing DDM generation (entries 5–8). However, we always observed particles around 10 nm, representing a dominant fraction corresponding to small clusters containing from several units to high tens of DDM molecules, depending on the generation. The formation of small clusters was also previously reported for other carbosilane glycodendrimers.^57^

**Figure 2:**
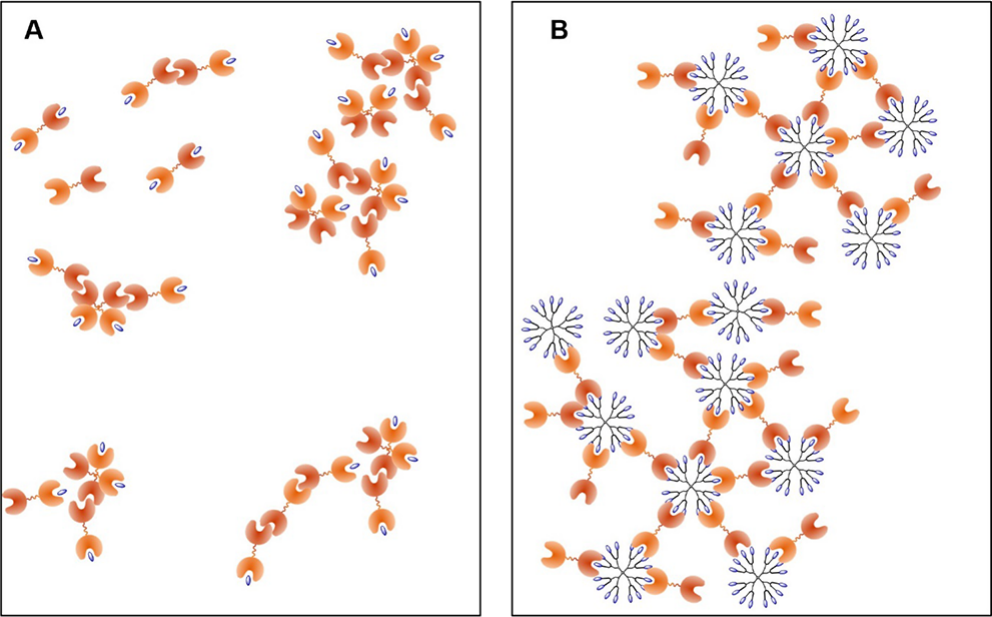
Tandem-type galectin–ligand interaction in solution: the difference between a monovalent (galectin–lactose; panel A) and multivalent system (galectin/Lac-CS-DDM; panel B).

Having an insight into the aggregation behavior of free galectins and glycodendrimers, we conducted a series of tests to evaluate the size distribution of Gal/Lac-CS-DDM assemblies following the study by Cloninger et al.^81^ Compared to single components, particles of a different size range were observed in mixed solutions (Table 2), indicating reorganization of the aggregates driven by mutual interaction and clustering. Thus, to reveal the influence of the glycodendrimer concentration on aggregation, we investigated different Gal/DDM ratios, including the environment of vast galectin excess. Control measurements of galectin/lactose mixtures were performed under the same conditions to assess the role of multivalency in the process. Although mutual aggregation driven by electrostatic and/or hydrophilic/hydrophobic interactions in galectin/lactose solutions is expected, we may not presume that the monovalent carbohydrate would directly cross-link multiple galectin molecules. On the contrary, this may occur in the multivalent Gal/Lac-CS-DDM system (Figure 2). Thus, a comparison between the Gal/free lactose and Gal/G_*n*_-A-Lac_2*m*_ solutions showed the influence of multivalency on the aggregation process. Data in entries 9–20 revealed that the aggregation tendency of all studied galectins slightly increased in the presence of monovalent lactose. Irrespective of the Gal/Lac ratio, relatively uniform particles were observed with Gal-3 (0.2–0.3 μm), Gal-8 (0.6–0.9 μm), and Gal-9 (0.7–1.0 μm); Gal-1 formed aggregates in a wider size range depending on the Lac concentration.

Next, we focused on the aggregation behavior of Gal/Lac-CS-DDM mixtures. Concerning Gal-1, the observed size diversity points to a very dynamic aggregation process. Particles in the size range of free Gal-1 together with small clusters were predominant in Gal-1/Lac-CS-DDM solutions, which indicates a poor aggregation tendency even at high DDM concentrations (entries 21–26). A certain level of aggregation was observed only with the third-generation dendrimers (ca. 0.2–0.4 μm aggregates; entries 27–32). This is in accordance with the findings by Cousin and Cloninger, who observed aggregation of Gal-1 with polyamidoamine-based glycodendrimers for the generations 3–6 having 20 or more Lac units at the periphery, but not with the second generation.^52^ On the other hand, chimera- and tandem-type galectins readily aggregated with Lac-CS-DDMs under the same experimental conditions. Gal-3 formed rather non-uniform associates with glycodendrimers (entries 33–44), and fractions of small particles corresponding either to free Gal-3 or to small Gal-3/Lac-CS-DDM clusters were apparent in most samples. On the other hand, tandem-type galectins formed rather uniform aggregates in the sub-micron size range for Gal-8 (entries 45–56) and in the range of 1–2 μm for Gal-9 (entries 57–68). In the case of Gal-8, the size of particles observed upon mixing with Lac-CS-DDMs is not fully indicative of the mutual association as also the free Gal-8 aggregates fall into similar size range (entry 3). Nevertheless, fractions of free galectin were apparent only under the conditions of a vast galectin excess.

Gal-9/Lac-CS-DDM solutions exhibited specific association features compared to other tested galectins: (i) very large aggregates of a relatively narrow size range were formed, and (ii) no fractions of small particles corresponding to either free Gal-9 or small Gal/Lac-CS-DDM clusters were detected. The presence of small particles cannot be ruled out, but compared to other studied galectin types, Gal-9 shows by far the highest degree of aggregation.

From the comparison of the results obtained from ELISA and DLS, we may relate the aggregation behavior with the Lac-CS-DDM affinity to galectins. In the case of Gal-1, the poor mutual association correlates with a low affinity increase, which was only 2–6 times higher in the multivalent system than for monovalent lactose (Table 1). In contrast, the affinity of the multivalent dendritic ligands to chimera and tandem galectins was an order of magnitude higher (Gal-3 and Gal-8) or even 2 orders of magnitude higher (Gal-9) compared to monovalent lactose. At the same time, glycodendrimers, even the first-generation ones, formed stable and distinct mutual associates with these galectins. In this case, the mutual aggregation may ease the ligand–protein interaction, leading to an increased affinity of the dendritic multivalent system. Vice versa, the dendritic effect-driven affinity enhancement may lead to mutual aggregation, further stabilizing the system. Such synergism may explain the outstanding affinity enhancement (560-fold better in Gal-9/G_3_-A-Lac_32_ than free lactose), which in the case of Gal-9/G_3_-B-Lac_32_ attacks the nanomolar affinity benchmark (1390-fold affinity enhancement over free lactose).

## 4 Conclusions

The study provides the very first multivalent galectin inhibitors based on CS scaffolds with a high inhibitory potency, especially to Gal-9. Lactose-decorated G_1_-G_3_ CS-DDMs were prepared using CuAAC click reaction from alkyne-terminated dendritic precursors. The Lac-CS-DDM series differed in the triazole ring position, being distanced from the Lac unit by a TEG linker (series A) or linked directly to the C-1 position of the carbohydrate moiety (series B). The inhibitory activity of the multivalent Lac-CS-DDM ligands was determined in the ELISA assay and compared to the free lactose. The inhibitory potency was generally higher in series B. Hence, we showed that positioning the triazole ring in the carbohydrate moiety neighborhood further enhances the affinity of the multivalent ligands. The participation of the triazole ring on the glycan-receptor binding will be further studied.

Generally, the affinity to the tested galectins increased with the generation. In G_*n*_-B-Lac_2*m*_, the affinity of G_3_ (32 peripheral Lac) was about 20 times higher than G_1_ (8 peripheral Lac). All Lac-CS-DDMs showed higher affinity to the tested galectins than free lactose. The most potent ligand was G_3_-B-Lac_32_, in which the affinity raised 13 times to Gal-1, 47 times to Gal-3, and almost 2 orders of magnitude (77 times) to Gal-8 compared to free lactose. Moreover, G_3_-B-Lac_32_ showed a solid 3 orders of magnitude (1400 times) higher affinity to Gal-9, reaching the nanomolar IC_50_ benchmark (970 nM). These findings fully support the dendritic effect principle: even simple ligands can achieve outstanding affinity to target receptors if presented in a multivalent manner.

In addition, for the first time, we demonstrated that the aggregation behavior is related to the inhibitory potency of the multivalent ligands. We studied the self- and mutual aggregation behavior of the tested galectins, glycodendrimers, and their mixtures by DLS. Both free galectins and glycodendrimers formed distinct and relatively stable self-associates. Lac-CS-DDMs formed large, stable, and uniform aggregates with Gal-3 (0.3–1.4 μm), Gal-8 (0.5–1.2 μm), and particularly with Gal-9 (1–2 μm). The mutual Gal-Lac-CS-DDM associates differed from the sole components in size, indicating that the interaction between the galectins and DDMs stabilizes the system by reassembling to mutual aggregates. This was particularly prominent in Gal-9/Lac-CS-DDM mixtures, in which the mutual aggregates (i) were formed even in the significant excess of Gal-9 (150:1) and (ii) their diameter was 2–3 times larger compared to the aggregates of the sole components. In contrast, indistinct, polydisperse, and dynamically reassembling aggregates were detected in Gal-1/Lac-CS-DDM mixtures. In conclusion, besides providing highly potent Gal-9 inhibitors, we showed that the increased affinity of the multivalent system is associated with the formation of stable, uniform aggregates. Therefore, the investigation of the aggregation behavior of multivalent ligands can serve as an indicative tool to estimate the inhibitory potency toward galectins.

## Supporting Information Available

Structures of Lac-CS-DDMs and analytical data for DDMs: series A and B; synthesis and analytical data for compounds **1** and **2**; ^1^H, ^13^C, ^29^Si, COSY, HSQC, HMBC and HSQC TOCSY NMR spectra of Lac-CS-DDMs; MALDI-TOF and IR spectra and GPC traces of selected Lac-CS-DDMs; characterization of Gal-1, Gal-3, Gal-8, and Gal-9; ELISA assay; examples of DLS (PDF)
