# Supplementary material for: Lactose-Functionalized Carbosilane Glycodendrimers Are Highly Potent Multivalent Ligands for Galectin-9 Binding: Increased Glycan Affinity to Galectins Correlates with Aggregation Behavior
Source: Biomacromolecules. 2023 Sep 8;24(11):4705–17. doi: 10.1021/acs.biomac.3c00426 (PMC10646984; doi:10.1021/acs.biomac.3c00426)
Supplement: Supplementary file 1 — bm3c00426_si_001.pdf [file bm3c00426_si_001.pdf]

SUPPORTING INFORMATION

Lactose-functionalized carbosilane glycodendrimers are highly potent multivalent ligands for galectin-9 binding: increased glycan affinity to galectins correlates with aggregation behavior

Monika Müllerová, Michaela Hovorková, Táňa Závodná, Lucie Červenková Šťastná, Alena Krupková, Vojtěch Hamala, Kateřina Nováková, Jan Topinka, Pavla Bojarová, and Tomáš Strašák

Institute of Chemical Process Fundamentals, Czech Academy of Sciences, Rozvojová 135, CZ-165 02 Prague, Czech Republic
Institute of Microbiology of the Czech Academy of Sciences, CZ-142 00 Prague, Czech Republic
Department of Genetics and Microbiology, Faculty of Science, Charles University, Viničná 5, CZ-12843, Prague 2, Czech Republic
Institute of Experimental Medicine, Czech Academy of Sciences, Vídeňská 1083, CZ-142 20 Prague, Czech Republic
Institute of Organic Chemistry and Biochemistry, Czech Academy of Sciences, Flemingovo nám. 2, CZ-166 10 Prague, Czech Republic

Table of Contents:

1. Structures of Lac-CS-DDMs. 2

2. Data for DDMs – series A. 3

3. Synthesis and data for compound 1 and 2. 6

4. Data for DDMs – series B 6

5. NMR spectra 10

6. MALDI-TOF, IR, GPC 39

7. Galectin Characterization 42

8. ELISA 44

9. Dynamic light scattering 46

References 49

1. Structures of Lac-CS-DDMs.

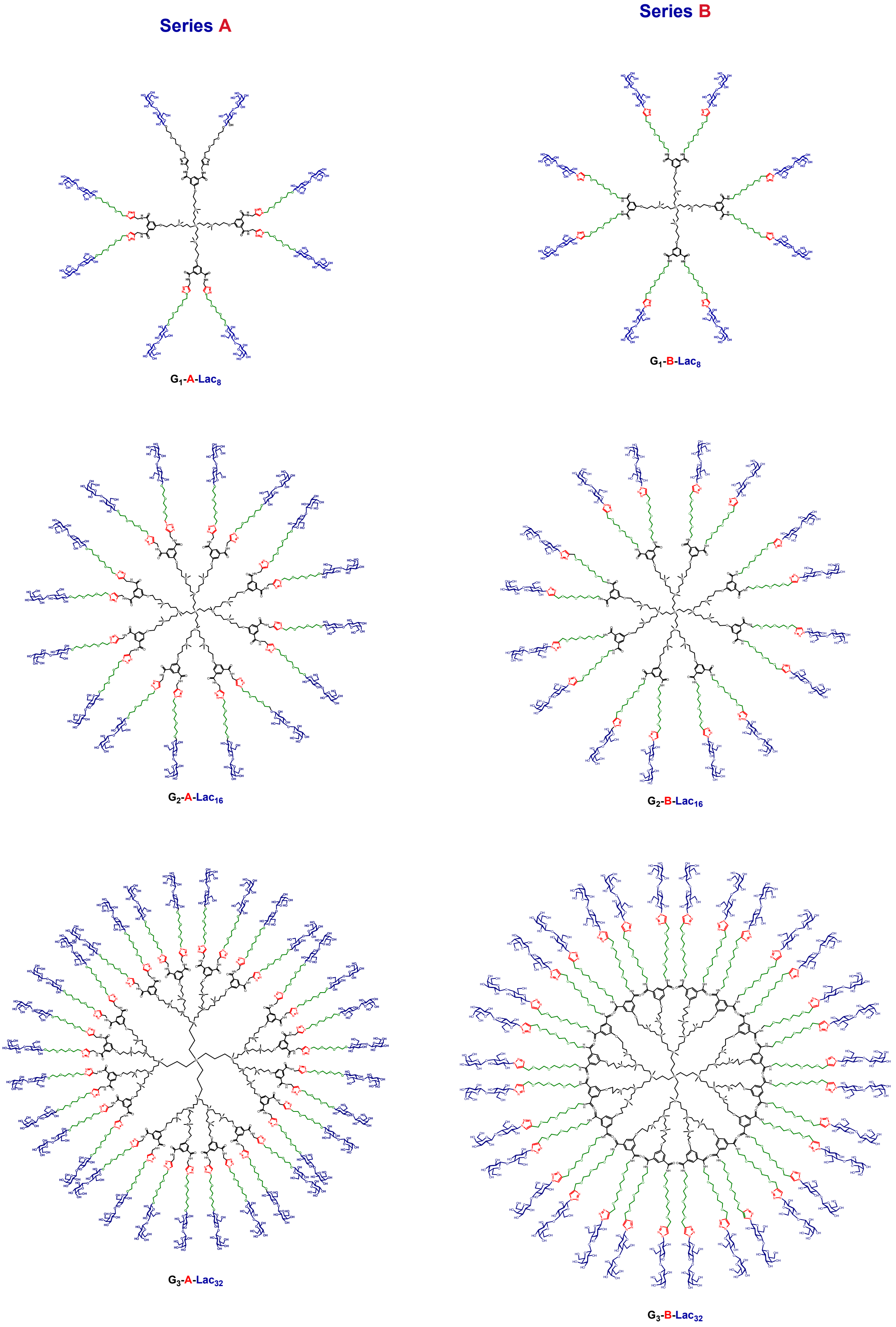

Figure S1: Structures of lactose-modified carbosilane dendrimers

2. Data for DDMs – series A.

Data for **G_1-A-AcLac$_8$ (12a):** ^{1}H NMR (400 MHz, dmso-*d_6*, H-H COSY): *δ* 9.03 (br s, 8H, N*H*), 7.94 (br s, 12H, C*H*$_{Tria}$, C*H*$_{Ph}$), 7.52 (br s, 8H, C*H*$_{Ph}$), 5.22 (d, *J* = 3.6 Hz, 8H, H-4′), 5.15 (dd, *J* = 10.4, 3.6 Hz, 8H, H-3′), 5.12 (dd, *J* = 9.9, 8.5 Hz, 8H, H-3), 4.83 (dd, *J* = 10.4, 8.0 Hz, 8H, H-2′), 4.73 (d, *J* = 8.0 Hz, 8H, H-1′), 4.72 (d, *J* = 7.9 Hz, 8H, H-1), 4.66 (dd, *J* = 9.9, 7.9 Hz, 8H, H-2), 4.49–4.46 (m, 32H, NC*H*$_2$), 4.29 (br d, *J* = 11.6 Hz, 8H, H-6$_a$), 4.21 (t, *J* = 6.8 Hz, 8H, H-5′), 4.06–3.94 (m, 32H, H-6$_b$, H-6′$_{a,b}$, C*H*$_2$OC$_q$), 3.78–3.70 (m, 40H, H-4, H-5, OCH*H*, OC*H*$_2$), 3.60–3.44 (m, 56H, OCH*H*, OC*H*$_2$), 2.09, 2.06 (2×s, 2×24H, *Me*$_{Ac}$), 1.99 (s, 48H, *Me*$_{Ac}$), 1.96, 1.94, 1.89 (3×s, 3×24H, *Me*$_{Ac}$), 1.68 (br s, 8H, OCH$_2$C*H*$_2$CH$_2$), 1.33 (br s, 8H, SiCH$_2$C*H*$_2$), 0.56 (m, 24H, SiC*H*$_2$), –0.05 (s, 24H, Si*Me*$_2$). ^{13}C {^{1}H} NMR (101 MHz, dmso-*d_6*, HSQC, HMBC, HSQCTOCSY): *δ* 170.2, 169.9, 169.8, 169.5, 169.3, 169.1, 169.0 (7×*C*O$_{Ac}$), 165.4 (*C*ONH), 158.5 (*C*$_q$O), 144.6 from HMBC (*C*$_{q(Tria)}$), 135.6 (*C*$_{q(Ph)}$), 123.5 (*C*H$_{Tria}$), 118.6 (*C*H$_{Ph}$), 115.8 (2*C*H$_{Ph}$), 99.9 (C-1′), 99.3 (C-1), 76.2 (C-4), 72.4 (C-3), 71.7 (C-5), 71.1 (C-2), 70.5 (*C*H$_2$OC$_q$), 70.2 (C-3′), 69.6 (C-5′, *C*H$_2$O), 69.4, 69.2 (2×O*C*H$_2$), 68.9 (C-2′), 68.7, 68.5 (2×O*C*H$_2$), 67.0 (C-4′), 62.2 (C-6), 60.7 (C-6′), 49.2 (CH$_2$*C*H$_2$N), 34.8 (*C*H$_2$NH), 23.3 (CH$_2$*C*H$_2$CH$_2$O), 20.5, 20.38, 20.36, 20.31, 20.30, 20.24, 20.23 (7×*Me*$_{Ac}$), 19.4 (Si^{0}CH$_2$CH$_2$*C*H$_2$), 18.2 (Si^{0}CH$_2$*C*H$_2$), 16.9 (Si^0*C*H$_2$), 10.9 (*C*H$_2$CH$_2$CH$_2$O), –3.5 (Si*Me*$_2$). ^{29}Si INEPT NMR (79 MHz, dmso-*d_6*): *δ* 2.18 (Si^1), 0.84 (Si^0).

Data for **G_1-A-Lac$_8$ (12b)**: ^{1}H NMR (400 MHz, dmso-*d_6*, H-H COSY): *δ* 9.03 (br s, 8H, N*H*), 7.94 (br s, 12H, C*H*$_{Tria}$, C*H*$_{Ph}$), 7.52 (br s, 8H, C*H*$_{Ph}$), 5.12 (d, *J* = 5.1 Hz, 8H, O*H*-2), 5.08 (d, *J* = 4.0 Hz, 8H, O*H*-2′), 4.78 (d, *J* = 4.9 Hz, 8H, O*H*-3′), 4.67 (s, 8H, O*H*-3), 4.64 (t, *J* = 5.0 Hz, 8H, O*H*-6′), 4.57 (t, *J* = 6.1 Hz, 8H, O*H*-6), 4.51–4.48 (m, 40H, NC*H*$_2$, O*H*-4′), 4.20 (d, *J* = 7.9 Hz, 8H, H-1), 4.19 (d, *J* = 8.0 Hz, 8H, H-1′), 3.96 (br s, 8H, C*H*$_2$OC$_q$), 3.85–3.76 (m, 24H, OCH*H*, OC*H*$_2$), 3.74 (dd, *J* = 12.7, 6.4 Hz, 8H, H-6$_a$), 3.61–3.45 (m, 96H, H-6$_b$, H-6′$_{a,b}$, H-4′, H-5′, 3C*H*$_2$O, OCH*H*), 3.33–3.28 (m, 40H, H-3, H-4, H-5, H-2′, H-3′), 3.01 (ddd, *J* = 8.1, 7.9, 5.1 Hz, 8H, H-2), 1.69 (br s, 8H, OCH$_2$C*H*$_2$CH$_2$), 1.34 (br s, 8H, Si^{0}CH$_2$C*H*$_2$), 0.56 (br s, 24H, SiC*H*$_2$), –0.05 (s, 24H, Si*Me*$_2$). ^{13}C {^{1}H} NMR (101 MHz, dmso-*d_6*, HSQC, HMBC, HSQCTOCSY): *δ* 165.4 (*C*O), 158.6 (*C*$_q$O), 144.8 (*C*$_{q(Tria)}$), 135.6 (*C*$_{q(Ph)}$), 123.4 (*C*H$_{Ph}$), 118.7 (*C*H$_{Tria}$), 115.9 (2*C*H$_{Ph}$), 103.9 (C-1′), 102.7 (C-1), 80.7 (C-4), 75.5 (C-5′), 75.0 (C-3), 74.8 (C-5), 73.2 (C-2′), 73.1 (C-2), 70.6 (*C*H$_2$OC$_q$, C-3′), 69.7, 69.6, 69.5, 68.7 (4×O*C*H$_2$), 68.2 (C-4′), 68.0 (O*C*H$_2$), 60.5 (C-6), 60.4 (C-6′), 49.3 (CH$_2$*C*H$_2$N), 34.9 (*C*H$_2$NH), 23.4 (CH$_2$*C*H$_2$CH$_2$O), 19.5 (Si^{0}CH$_2$CH$_2$*C*H$_2$), 18.2 (Si^{0}CH$_2$*C*H$_2$), 17.0 (Si^0*C*H$_2$), 11.0 (*C*H$_2$CH$_2$CH$_2$O), –3.4 (Si*Me*$_2$). ^{29}Si INEPT NMR (79 MHz, dmso-*d_6*): *δ* 2.20 (Si^1), 0.86 (Si^0).

MALDI-TOF MS: $[C_{232}H_{380}N_{32}NaO_{116}Si_5]^+$ *m/z* calc. 5633.356 $[M+Na]^+$, *m/z* found 5633.363 (monoiz.).

Data for **G2-A-AcLac16 (13a):** ^{1}H NMR (400 MHz, dmso-d_6, H-H COSY): δ 9.01 (br s, 16H, N*H*), 7.94 (br s, 24H, CH_{Tria}, CH_{Ph}), 7.51 (br s, 16H, CH_{Ph}), 5.21 (d, *J* = 3.5 Hz, 16H, H-4′), 5.14 (dd, *J* = 10.4, 3.5 Hz, 16H, H-3′), 5.12 (dd, *J* = 9.9, 8.5 Hz, 16H, H-3), 4.83 (dd, *J* = 10.2, 8.0 Hz, 16H, H-2′), 4.73 (d, *J* = 8.0 Hz, 16H, H-1′), 4.72 (d, *J* = 7.9 Hz, 16H, H-1), 4.65 (dd, *J* = 9.9, 7.9 Hz, 16H, H-2), 4.46 (br s, 64H, NCH_2), 4.29 (br d, *J* = 11.9 Hz, 16H, H-6_a), 4.21 (t, *J* = 6.8 Hz, 16H, H-5′), 4.06–3.94 (m, 64H, H-6_b, H-$6'_{a,b}$, CH_2OC_q), 3.78–3.70 (m, 80H, H-4, H-5, OCH*H*, OCH_2), 3.60–3.43 (m, 112H, OCH*H*, OCH_2), 2.08, 2.05 (2×s, 2×48H, Me_{Ac}), 1.98 (s, 96H, 2×Me_{Ac}), 1.95, 1.93, 1.88 (3×s, 3×48H, Me_{Ac}), 1.68 (br s, 16H, OCH_2CH_2CH_2), 1.33 (br s, 24H, $Si^{0,1}$CH_2CH_2), 0.54 (br s, 64H, SiCH_2), –0.06 (s, 48H, Si^2Me_2), –0.11 (s, 12H, Si^1Me). ^{13}C {^{1}H} NMR (101 MHz, dmso-d_6, HSQC, HMBC, HSQCTOCSY): δ 170.2, 169.81, 169.77, 169.4, 169.2, 169.1, 169.0 (7×CO_{Ac}), 165.3 (*C*ONH), 158.5 (C_qO), not detected ($C_{q(Tria)}$), 135.5 ($C_{q(Ph)}$), 123.5 (CH_{Tria}), 118.6 (CH_{Ph}), 115.8 (2CH_{Ph}), 99.9 (C-1′), 99.2 (C-1), 76.2 (C-4), 72.3 (C-3), 71.6 (C-5), 71.1 (C-2), 70.5 (CH_2OC_q), 70.2 (C-3′), 69.6 (C-5′, CH_2O), 69.4, 69.2 (2×OCH_2), 68.8 (C-2′), 68.7, 68.5 (2×OCH_2), 67.0 (C-4′), 62.1 (C-6), 60.7 (C-6′), 49.2 (CH_2CH_2N), 34.8 (CH_2NH), 23.3 ($CH_2CH_2CH_2$O), 20.5 (Me_{Ac}), 20.34 (2×Me_{Ac}), 20.33, 20.26, 20.21, 20.19 (4×Me_{Ac}), 19.3 ($Si^1CH_2CH_2CH_2Si^2$), not detected or overlapped ($Si^0CH_2CH_2$), 18.2 ($Si^1CH_2CH_2CH_2Si^2$), 18.0 ($Si^1CH_2CH_2CH_2Si^2$), 17.1 (Si^0CH_2), 10.8 ($CH_2CH_2CH_2$O), -3.5 (Si^2Me_2), -4.9 (Si^1Me). ^{29}Si INEPT NMR (79 MHz, dmso-d_6): δ 2.16 (Si^2), 1.08 (Si^1), not detected (Si^0).

Data for **G2-A-Lac16 (13b)**: ^{1}H NMR (400 MHz, dmso-d_6, H-H COSY): δ 9.02 (br s, 16H, N*H*), 7.94 (br s, 24H, CH_{Tria}, CH_{Ph}), 7.52 (br s, 16H, CH_{Ph}), 5.12 (d, *J* = 5.1 Hz, 16H, O*H*-2), 5.08 (d, *J* = 4.0 Hz, 16H, O*H*-2′), 4.77 (d, *J* = 5.0 Hz, 16H, O*H*-3′), 4.67 (s, 16H, O*H*-3), 4.64 (t, *J* = 5.0 Hz, 16H, O*H*-6′), 4.56 (t, *J* = 6.1 Hz, 16H, O*H*-6), 4.51–4.47 (m, 80H, NCH_2, O*H*-4′), 4.21 (d, *J* = 7.9 Hz, 16H, H-1), 4.20 (d, *J* = 8.0 Hz, 16H, H-1′), 3.95 (br s, 16H, CH_2OC_q), 3.84–3.72 (m, 64H, H-6_a, OCH*H*, OCH_2), 3.62–3.45 (m, 192H, H-6_b, H-$6'_{a,b}$, H-4′, H-5′, 3CH_2O, OCH*H*), 3.32–3.28 (m, 80H, H-3, H-4, H-5, H-2′, H-3′), 3.01 (ddd, *J* = 8.4, 7.9, 5.0 Hz, 16H, H-2), 1.68 (br s, 16H, OCH_2CH_2CH_2), 1.32 (br s, 24H, $Si^{0,1}$CH_2CH_2), 0.56 (br s, 64H, SiCH_2), –0.05 (s, 48H, Si^2Me_2), –0.10 (s, 12H, Si^1Me). ^{13}C {^{1}H} NMR (101 MHz, dmso-d_6, HSQC, HMBC, HSQCTOCSY): δ 165.4 (*C*O), 158.6 (C_qO), 144.7 ($C_{q(Tria)}$), 135.6 ($C_{q(Ph)}$), 123.4 (CH_{Ph}), 118.6 (CH_{Tria}), 115.8 (2CH_{Ph}), 103.9 (C-1′), 102.6 (C-1), 80.7 (C-4), 75.5 (C-5′), 75.0 (C-3), 74.8 (C-5), 73.2 (C-2′), 73.1 (C-2), 70.5 (CH_2OC_q, C-3′), 69.63, 69.59, 69.5, 68.7

(4×O*C*H$_2$), 68.1 (C-4′), 68.0 (O*C*H$_2$), 60.5 (C-6), 60.4 (C-6′), 49.3 (CH$_2$*C*H$_2$N), 34.9 (*C*H$_2$NH), 23.3 (CH$_2$*C*H$_2$CH$_2$O), 19.4 (Si^{1}CH$_2$CH$_2$*C*H$_2$Si^2), 18.5 (Si^{0}CH$_2$CH$_2$*C*H$_2$), overlapped (Si^{0}CH$_2$*C*H$_2$), 18.3 (Si^{1}CH$_2$*C*H$_2$CH$_2$Si^2), 18.1 (Si^1*C*H$_2$CH$_2$CH$_2$Si^2), 17.1 (Si^0*C*H$_2$), 10.9 (*C*H$_2$CH$_2$CH$_2$O), -3.4 (Si^2*Me*$_2$), -4.8 (Si^1*Me*). ^{29}Si INEPT NMR (79 MHz, dmso-*d*$_6$): δ 2.18 (Si^2), 1.10 (Si^1), 0.86 (Si^0).

MALDI-TOF MS: [$C_{480}H_{796}N_{64}NaO_{232}Si_{13}$]$^+$ *m/z* calc. 11563.9 [M+Na]$^+$, *m/z* found 11564.4 (average mass).

Data for **G$_3$-A-AcLac$_{32}$ (14a)**: ^{1}H NMR (400 MHz, dmso-*d*$_6$, H-H COSY): δ 8.99 (br s, 32H, N*H*), 7.94 (br s, 48H, C*H*$_{\mathrm{Tria}}$, C*H*$_{\mathrm{Ph}}$), 7.50 (br s, 32H, C*H*$_{\mathrm{Ph}}$), 5.21 (d, *J* = 3.5 Hz, 32H, H-4′), 5.14 (dd, *J* = 10.4, 3.5 Hz, 32H, H-3′), 5.12 (dd, *J* = 9.9, 8.5 Hz, 32H, H-3), 4.82 (dd, *J* = 10.1, 7.9 Hz, 32H, H-2′), 4.73 (d, *J* = 7.9 Hz, 32H, H-1′), 4.72 (d, *J* = 7.9 Hz, 32H, H-1), 4.65 (dd, *J* = 9.9, 7.9 Hz, 32H, H-2), 4.45 (br s, 128H, NC*H*$_2$), 4.28 (br d, *J* = 11.6 Hz, 32H, H-6$_a$), 4.20 (t, *J* = 6.7 Hz, 32H, H-5′), 4.06–3.92 (m, 128H, H-6$_b$, H-6′$_{a,b}$, C*H*$_2$OC$_q$), 3.77–3.70 (m, 160H, H-4, H-5, OCH*H*, OC*H*$_2$), 3.56–3.42 (m, 224H, OCH*H*, OC*H*$_2$), 2.08, 2.04 (2×s, 2×96H, *Me*$_{\mathrm{Ac}}$), 1.98 (s, 192H, 2×*Me*$_{\mathrm{Ac}}$), 1.95, 1.93, 1.88 (3×s, 3×96H, *Me*$_{\mathrm{Ac}}$), 1.69 (br s, 32H, OCH$_2$C*H*$_2$CH$_2$), 1.31 (br s, 56H, Si^{0,1,2}CH$_2$C*H*$_2$), 0.53 (br s, 144H, SiC*H*$_2$), –0.07 (s, 96H, Si^3*Me*$_2$), –0.11 (s, 36H, Si^{1,2}*Me*). ^{13}C {^{1}H} NMR (101 MHz, dmso-*d*$_6$, HSQC, HMBC, HSQCTOCSY): δ 170.1, 169.8, 169.7, 169.4, 169.2, 169.1, 169.0 (7×*C*O$_{\mathrm{Ac}}$), 165.3 (*C*ONH), 158.5 (*C*$_q$O), not detected (*C*$_{\mathrm{q(Tria)}}$), 135.5 (*C*$_{\mathrm{q(Ph)}}$), 123.6 (*C*H$_{\mathrm{Tria}}$), 118.4 (*C*H$_{\mathrm{Ph}}$), 115.7 (2*C*H$_{\mathrm{Ph}}$), 99.8 (C-1′), 99.2 (C-1), 76.2 (C-4), 72.3 (C-3), 71.6 (C-5), 71.1 (C-2), 70.4 (*C*H$_2$OC$_q$), 70.2 (C-3′), 69.6 (C-5′, *C*H$_2$O), 69.4, 69.2 (2×O*C*H$_2$), 68.8 (C-2′), 68.6, 68.4 (2×O*C*H$_2$), 66.9 (C-4′), 62.1 (C-6), 60.7 (C-6′), 49.2 (CH$_2$*C*H$_2$N), 34.9 (*C*H$_2$NH), 23.2 (CH$_2$*C*H$_2$CH$_2$O), 20.4 (*Me*$_{\mathrm{Ac}}$), 20.3, 20.22, 20.16 (3×2*Me*$_{\mathrm{Ac}}$), 19.3 (Si^{2}CH$_2$CH$_2$*C*H$_2$Si^3), not detected or overlapped (Si$^{0/1}$*C*H$_2$*C*H$_2$*C*H$_2$Si$^{1/2}$), 18.2 (Si^{2}CH$_2$*C*H$_2$CH$_2$Si^3), 18.0 (Si^2*C*H$_2$CH$_2$CH$_2$Si^3), 10.8 (*C*H$_2$CH$_2$CH$_2$O), –3.6 (Si^3*Me*$_2$), –5.0 (Si^{1,2}*Me*). ^{29}Si INEPT NMR (79 MHz, dmso-*d*$_6$): δ 2.16 (Si^2), 1.08 (Si^1), not detected (Si^0).

Data for **G$_3$-A-Lac$_{32}$ (14b):** ^{1}H NMR (400 MHz, dmso-*d*$_6$, H-H COSY): δ 8.99 (br s, 32H, N*H*), 7.94 (br s, 48H, C*H*$_{\mathrm{Tria}}$, C*H*$_{\mathrm{Ph}}$), 7.50 (br s, 32H, C*H*$_{\mathrm{Ph}}$), 5.13 (d, *J* = 5.1 Hz, 32H, O*H*-2), 5.08 (d, *J* = 3.8 Hz, 32H, O*H*-2′), 4.77 (d, *J* = 4.5 Hz, 32H, O*H*-3′), 4.68 (s, 32H, O*H*-3), 4.64 (t, *J* = 5.0 Hz, 32H, O*H*-6′), 4.57 (t, *J* = 6.1 Hz, 32H, O*H*-6), 4.51–4.47 (m, 160H, NC*H*$_2$, O*H*-4′), 4.21 (d, *J* = 7.9 Hz, 32H, H-1), 4.20 (d, *J* = 8.0 Hz, 32H, H-1′), 3.93 (br s, 32H, C*H*$_2$OC$_q$), 3.83–3.72 (m, 128H, H-6$_a$, OCH*H*, OC*H*$_2$), 3.62–3.45 (m, 192H, H-6$_b$, H-6′$_{a,b}$, H-4′, H-5′, 3×C*H*$_2$O, OCH*H*), 3.31–3.28 (m, 160H, H-3, H-4, H-5, H-2′, H-3′), 3.01 (ddd, *J* = 9.9, 7.9, 5.1 Hz, 32H, H-2), 1.67 (br s, 32H, OCH$_2$C*H*$_2$CH$_2$), 1.32 (br s, 56H, Si^{0,1,2}CH$_2$C*H*$_2$), 0.54 (br s, 144H,

SiCH_2), –0.06 (s, 96H, Si^3Me_2), –0.10 (s, 36H, Si^{1,2}Me). ^{13}C {^{1}H} NMR (101 MHz, dmso-d_6, HSQC, HMBC, HSQCTOCSY): δ 165.4 (CO), 158.6 (C_qO), not detected ($C_{q(Tria)}$), 135.6 ($C_{q(Ph)}$), 123.6 (CH$_{Tria}$), 118.5 (CH$_{Ph}$), 115.9 (2CH$_{Ph}$), 103.8 (C-1′), 102.6 (C-1), 80.7 (C-4), 75.5 (C-5′), 75.0 (C-3), 74.8 (C-5), 73.2 (C-2′), 73.1 (C-2), 70.5 (CH$_2$OC$_q$, C-3′), 69.6 (2×OCH$_2$), 69.5, 68.7 (2×OCH$_2$), 68.1 (C-4′), 68.0 (OCH$_2$), 60.5 (C-6), 60.4 (C-6′), 49.3 (CH$_2$$CH_2$N), 34.9 ($CH_2$NH), 23.3 (CH$_2$$CH_2CH_2$O), 19.4 (Si^{2}CH$_2CH_2$$CH_2$Si^3), not detected or overlapped (Si$^{0/1}$$CH_2$$CH_2$$CH_2Si^{1/2}$), 18.2 (Si^{2}CH$_2$$CH_2CH_2$Si^3), 18.1 (Si^2$CH_2CH_2CH_2$Si^3), 10.9 ($CH_2CH_2CH_2$O), –3.5 (Si^3$Me_2$), –4.9 (Si^{1,2}$Me$). ^{29}Si INEPT NMR (79 MHz, dmso-d_6): δ 2.15 (Si^3), 1.08 (Si^{1,2}), not detected (Si^0).

3. Synthesis and data for compound 1 and 2.

The suspension of 5-(OMOM)isophtalic acid was dissolved in dry DMF and cooled in an ice bath. Then, EDC·HCl (15.8 g, 82.3 mmol), HOBt (11.1 g, 82.3 mmol) and NMM (25.3 mL; 230.5 mmol;) were added portionwise. After 45 min of vigorous stirring, the 2-(2-(2-(prop-2-yn-1-yloxy)ethoxy)ethoxy)ethan-1-amine (5.3 mL, 82.3 mmol) was added dropwise. Then, the reaction mixture was stirred overnight. The TLC analysis (chloroform/methanol 10:1) indicated the completion of the reaction. The reaction mixture was evaporated, the solid was dissolved in ethyl acetate and extracted with water (2 x) and brine (1x). Collected organic phases were dried over MgSO4, filtered and evaporated to dryness to obtain compound **1** as off-white viscous liquid (84 % yield). Then, the liquid was dissolved in MeOH and ion exchange resin (Dowex® 50W X8) in hydrogen form was added. The reaction mixture was heated at 45 °C overnight. The TLC analysis (chloroform/methanol 95:5; Rf = 0.05) indicated completion of the reaction. The resin was filtered off and washed with methanol. The collected organic phases were evaporated under vacuum to obtain an off-white crystal solid of compound **2**.

Data for **2:** ^{1}H NMR (CDCl$_3$, 400 MHz, H−H COSY): δ 8.47 (t, J = 5.6 Hz, 2H, NH), 7.72 (t, J = 1.5 Hz, 1H, CH_{Ph}), 7.34 (d, J = 1.5 Hz, 2H, CH_{Ph}), 4.11 (d, J = 2.4 Hz, 4H, OCH_2C≡), 3.53–3.51 (m, 22H, OCH_2), 3.45–3.41 (m, 6H, HNCH_2, OCH_2), 3.38 (t, J = 2.4 Hz, 2H, C≡CH). ^{13}C{^{1}H} NMR (CDCl$_3$, 101 MHz, HSQC, HMBC): δ 166.1 (CO), 157.4 (C_qOH), 136.1 ($C_{q(Ph)}$), 116.83 (2CH$_{Ph}$), 116.79 (CH$_{Ph}$), 80.4 (C≡CH), 77.2 (C≡CH), 69.8, 69.7, 69.6, 68.9, 68.6 (5×OCH$_2$), 57.6 (OCH$_2$C≡), overlapped with DMSO from HSQC 39.6 (HNCH$_2$).

4. Data for DDMs – series B.

Data for **G$_1$-B (9):** ^{1}H NMR (CDCl$_3$, 400 MHz, H−H COSY): δ 7.74 (t, J = 1.4 Hz, 4H, CH_{Ph}), 7.46 (d, J = 1.4 Hz, 8H, CH_{Ph}), 7.27 (t, J = 5.5 Hz, 8H, NH), 4.11 (d, J = 2.4 Hz, 16H, OCH_2C≡), 3.92 (t, J = 6.8 Hz, 8H, OCH_2CH$_2$CH$_2$), 3.65–3.64 (m, 96H, HNCH_2, OCH_2), 2.41 (t, J = 2.4 Hz,

8H, C≡C*H*), 1.77–1.71 (m, 8H, OCH$_2$C*H*$_2$CH$_2$), 1.35–1.29 (m, 8H, Si^{0}CH$_2$C*H*$_2$), 0.61–0.53 (m, 24H, SiC*H*$_2$), –0.02 (s, 24H, Si*Me*$_2$). ^{13}C{^{1}H} NMR (CDCl$_3$, 101 MHz, HSQC): δ 166.9 (*C*O), 159.6 (*C*$_q$O), 136.0 (*C*$_{q(Ph)}$), 117.1 (*C*H$_{Ph}$), 116.6 (2*C*H$_{Ph}$), 79.6 (*C*≡CH), 74.9 (C≡*C*H), 71.3 (O*C*H$_2$CH$_2$CH$_2$), 70.7, 70.4, 70.3, 69.9, 69.1 (5×O*C*H$_2$), 58.4 (O*C*H$_2$C≡), 40.1 (HN*C*H$_2$), 23.9 (OCH$_2$*C*H$_2$CH$_2$), 20.2 (Si^{0}CH$_2$CH$_2$*C*H$_2$), 18.7 (Si^{0}CH$_2$*C*H$_2$), 17.6 (Si^0*C*H$_2$), 11.4 (*C*H$_2$CH$_2$CH$_2$O), –3.3 (Si*Me*$_2$). ^{29}Si INEPT NMR (CDCl$_3$, 80 MHz): δ 2.18 (4Si), 0.63 (1Si).

Data for **G$_2$-B (10):** ^{1}H NMR (CDCl$_3$, 400 MHz, H−H COSY): δ 7.72 (br s, 8H, C*H*$_{Ph}$), 7.45 (br s, 16H, C*H*$_{Ph}$), 7.23 (br s, 16H, N*H*), 4.10 (d, *J* = 2.4 Hz, 32H, OC*H*$_2$C≡), 3.91 (br s, 16H, OC*H*$_2$CH$_2$CH$_2$), 3.64 (br s, 192H, OC*H*$_2$, HNC*H*$_2$), 2.41 (t, *J* = 2.4 Hz, 16H, C≡C*H*), 1.74 (br s, 16H, OCH$_2$C*H*$_2$CH$_2$), 1.31 (m, 24H, SiCH$_2$C*H*$_2$), 0.56 (m, 64H, SiC*H*$_2$), –0.02 (s, 48H, Si*Me*$_2$), –0.07 (s, 12H, Si*Me*). ^{13}C{^{1}H} NMR (CDCl$_3$, 101 MHz, HSQC, HMBC): δ 166.9 (*C*O), 159.6 (*C*$_q$O), 136.1 (*C*$_{q(Ph)}$), 117.1 (*C*H$_{Ph}$), 116.6 (2*C*H$_{Ph}$), 79.6 (*C*≡CH), 74.9 (C≡*C*H), 71.3 (O*C*H$_2$CH$_2$CH$_2$), 70.7, 70.44, 70.38, 69.9, 69.2 (5×O*C*H$_2$), 58.4 (O*C*H$_2$C≡), 40.1 (HN*C*H$_2$), 23.9 (OCH$_2$*C*H$_2$CH$_2$), 20.1 (Si^{1}CH$_2$CH$_2$*C*H$_2$Si^2), 19.3 (Si^{0}CH$_2$CH$_2$*C*H$_2$), 18.9 (Si^1*C*H$_2$CH$_2$CH$_2$Si^2), 18.7 (Si^{0}CH$_2$*C*H$_2$), 18.6 (Si^{1}CH$_2$*C*H$_2$CH$_2$Si^2), 17.8 (Si^0*C*H$_2$), 11.4 (*C*H$_2$CH$_2$CH$_2$O), –3.2 (Si*Me*$_2$), –4.8 (Si*Me*). ^{29}Si INEPT NMR (CDCl$_3$, 80 MHz): δ 2.21 (8Si), 0.96 (4Si), 0.53 (1Si).

Data for **G$_3$-B (11):** ^{1}H NMR (dmso-*d$_6$*, 400 MHz, H−H COSY): δ 8.52 (br s, 32H, N*H*), 7.89 (br s, 16H, C*H*$_{Ph}$), 7.47 (br s, 32H, C*H*$_{Ph}$), 4.09 (d, *J* = 2.3 Hz, 64H, OC*H*$_2$C≡), 3.93 (t, *J* = 6.8 Hz, 32H, OC*H*$_2$CH$_2$CH$_2$), 3.52–3.50 (m, 320H, OC*H*$_2$), 3.38 (m, 64H, HNC*H*$_2$), 3.33 (s, 32H, C≡C*H*), 1.67 (m, 32H, OCH$_2$C*H*$_2$CH$_2$), 1.30 (m, 56H, Si$^{0/1/2}$CH$_2$C*H*$_2$), 0.53 (m, 144H, SiC*H*$_2$), –0.06 (s, 96H, Si*Me*$_2$), –0.11 (s, 36H, Si$^{1/2}$*Me*). ^{13}C{^{1}H} NMR (dmso-*d$_6$*, 101 MHz, HSQC, HMBC): δ 165.7 (*C*O), 158.6 (*C*$_q$O), 135.8 (*C*$_{q(Ph)}$), 118.3 (*C*H$_{Ph}$), 115.7 (2*C*H$_{Ph}$), 80.2 (*C*≡CH), 76.9 (C≡*C*H), 70.5 (O*C*H$_2$CH$_2$CH$_2$), 69.7, 69.6, 69.5, 68.9, 68.5 (5×O*C*H$_2$), 57.5 (O*C*H$_2$C≡), from HSQC 39.3 (HN*C*H$_2$), 23.4 (OCH$_2$*C*H$_2$CH$_2$), 19.4 (Si^{2}CH$_2$CH$_2$*C*H$_2$Si^3), not detected or overlapped (Si^0*C*H$_2$*C*H$_2$*C*H$_2$Si^1), 18.4, 18.2 (Si^1*C*H$_2$*C*H$_2$*C*H$_2$Si^2), 18.3 (Si^{2}CH$_2$*C*H$_2$CH$_2$Si^3), 18.1 (Si^2*C*H$_2$CH$_2$CH$_2$Si^3), 10.9 (*C*H$_2$CH$_2$CH$_2$O), –3.4 (Si^3*Me*$_2$), –4.9 (Si^{1,2}*Me*). ^{29}Si INEPT NMR (dmso-*d$_6$*, 80 MHz): δ 2.11 (16Si), 1.06 (12Si). 0.76 (1Si).

Data for **G$_1$-B-Lac$_8$ (15b):** ^{1}H NMR (400 MHz, dmso-*d$_6$*, H-H COSY): δ 8.58 (t, *J* = 5.6 Hz, 8H, N*H*), 8.28 (s, 8H, C*H*$_{Tria}$), 7.90 (s, 4H, C*H*$_{Ph}$), 7.49 (s, 8H, C*H*$_{Ph}$), 5.62 (d, *J* = 9.3 Hz, 8H, H-1), 5.55 (d, *J* = 6.0 Hz, 8H, O*H*-2), 5.12 (d, *J* = 4.3 Hz, 8H, O*H*-2′), 4.89 (d, *J* = 1.8 Hz, 8H, O*H*-3), 4.81 (d, *J* = 4.9 Hz, 8H, O*H*-3′), 4.68–4.65 (m, 16H, O*H*-6/6′), 4.54 (d, *J* = 4.7 Hz, 8H, O*H*-4′), 4.52 (s, 16H, OCH$_2$C*H*$_2$), 4.25 (d, *J* = 6.9 Hz, 8H, H-1′), 3.97 (t, *J* = 6.1 Hz, 8H,

CH_2OC_q), 3.85 (ddd, *J* = 9.3, 8.7, 6.0 Hz, 8H, H-2), 3.77 (dd, *J* = 10.7, 5.8 Hz, 8H, H-6$_a$), 3.67–3.45 (m, 160H, H-3, H-4, H-5, H-6b, H-4′, H-5′, H-6′a,b, OCH_2), 3.41–3.39 (m, 16H, CH_2NH), overlapped with water from HSQC 3.34 (8H, H-2′), overlapped with water from HSQC 3.33 (8H, H-3′), 1.69 (br s, 8H, $OCH_2CH_2CH_2$), 1.34 (br s, 8H, $SiCH_2CH_2$), 0.57 (br s, 24H, SiCH_2), -0.05 (s, 24H, Si*Me*$_2$). ^{13}C {^{1}H} NMR (101 MHz, dmso-*d*$_6$, HSQC, HMBC, HSQCTOCSY): *δ* 165.6 (*C*O), 158.5 (*C*$_q$O), 143.9 (*C*$_{q(Tria)}$), 135.9 (*C*$_{q(Ph)}$), 123.2 (*C*H$_{Tria}$), 118.4 (*C*H$_{Ph}$), 115.7 (2*C*H$_{Ph}$), 103.8 (C-1′), 87.0 (C-1), 79.8 (C-4), 77.8 (C-5), 75.6 (C-5′), 75.2 (C-3), 73.3 (C-3′), 71.8 (C-2), 70.6 (C-2′), 70.5 (*C*H$_2$OC$_q$), 69.72, 69.69, 69.66, 69.6, 69.1, 68.9 (6×O*C*H$_2$), 68.2 (C-4′), 63.4 (OCH$_2$*C*H$_2$), 60.5 (C-6′), 60.1 (C-6), overlapped with dmso from HSQC 39.2 (*C*H$_2$NH), 23.4 (CH$_2$*C*H$_2$CH$_2$O), 19.5 (SiCH$_2$CH$_2$*C*H$_2$), 18.2 (SiCH$_2$*C*H$_2$), 17.0 (Si*C*H$_2$), 10.9 (*C*H$_2$CH$_2$CH$_2$O), –3.3 (Si*Me*$_2$). ^{29}Si INEPT NMR (79 MHz, dmso-*d*$_6$): *δ* 2.19 (Si^1), 0.87 (Si^0). MALDI-TOF MS: [$C_{232}H_{380}N_{32}NaO_{116}Si_5$]$^+$ *m/z* calc. 5633.356 [M+Na]$^+$, *m/*z found 5633.342 (monoiz.).

Data for **G$_2$-B-Lac$_{16}$ (16b):** ^{1}H NMR (400 MHz, dmso-*d*$_6$, H-H COSY): *δ* 8.56 (br s, 16H, N*H*), 8.28 (s, 16H, C*H*$_{Tria}$), 7.90 (s, 8H, C*H*$_{Ph}$), 7.48 (br s, 16H, C*H*$_{Ph}$), 5.62 (d, *J* = 9.3 Hz, 16H, H-1), 5.56 (d, *J* = 5.8 Hz, 16H, O*H*-2), 5.13 (br s, 16H, O*H*-2′), 4.89 (br s, 16H, O*H*-3), 4.80 (br s, 16H, O*H*-3′), 4.67 (br s, 32H, O*H*-6/6′), 4.55–4.51 (m, 48H, OCH$_2$C*H*$_2$, O*H*-4′), 4.26 (d, *J* = 6.9 Hz, 16H, H-1′), 3.95 (br s, 16H, C*H*$_2$OC$_q$), 3.88–3.82 (m, 16H, H-2), 3.77 (dd, *J* = 10.9, 4.6 Hz, 16H, H-6$_a$), 3.63–3.45 (m, 320H, H-3, H-4, H-5, H-6$_b$, H-4′, H-5′, H-6′$_{a,b}$, OC*H*$_2$), 3.40 (br s, 32H, C*H*$_2$NH), 3.34 (br s, 32H, H-2′, H-3′), 1.68 (br s, 16H, OCH$_2$C*H*$_2$CH$_2$), 1.31 (br s, 24H, Si^{0,1}CH$_2$C*H*$_2$), 0.54 (br s, 64H, SiC*H*$_2$), –0.05 (s, 48H, Si^2*Me*$_2$), –0.10 (s, 12H, Si^1*Me*). ^{13}C {^{1}H} NMR (101 MHz, dmso-*d*$_6$, HSQC, HMBC, HSQCTOCSY): *δ* 165.6 (*C*O), 158.5 (*C*$_q$O), 143.9 (*C*$_{q(Tria)}$), 135.8 (*C*$_{q(Ph)}$), 123.2 (*C*H$_{Tria}$), 118.2 (*C*H$_{Ph}$), 115.7 (2*C*H$_{Ph}$), 103.8 (C-1′), 87.0 (C-1), 79.8 (C-4), 77.7 (C-5), 75.6 (C-5′), 75.2 (C-3), 73.3 (C-3′), 71.8 (C-2), 70.6 (C-2′), 70.5 (*C*H$_2$OC$_q$), 69.70, 69.67, 69.6, 69.1, 68.9 (6×O*C*H$_2$), 68.2 (C-4′), 63.4 (OCH$_2$*C*H$_2$), 60.4 (C-6′), 60.1 (C-6), 39.3 (*C*H$_2$NH), 23.3 (CH$_2$*C*H$_2$CH$_2$O), 19.4 (Si^{1}CH$_2$CH$_2$*C*H$_2$Si^2), 18.5 (Si^{0}CH$_2$CH$_2$*C*H$_2$), 18.3 (*C*H$_2$CH$_2$Si^{1}CH$_2$*C*H$_2$), 18.1 (Si^1*C*H$_2$CH$_2$CH$_2$Si^2), 17.1 (Si^0*C*H$_2$), 10.9 (*C*H$_2$CH$_2$CH$_2$O), –3.4 (Si^2*Me*$_2$), –4.8 (Si^1*Me*). ^{29}Si INEPT NMR (79 MHz, dmso-*d*$_6$): *δ* 2.16 (Si^2), 1.08 (Si^1), 0.81 (Si^0).

Data for **G$_3$-B-Lac$_{32}$ (17b):** ^{1}H NMR (400 MHz, dmso-*d*$_6$, H-H COSY): *δ* 8.55 (br s, 32H, N*H*), 8.28 (s, 32H, C*H*$_{Tria}$), 7.89 (s, 16H, C*H*$_{Ph}$), 7.48 (br s, 32H, C*H*$_{Ph}$), 5.62 (d, *J* = 9.3 Hz, 32H, H-1), 5.55 (d, *J* = 5.8 Hz, 32H, O*H*-2), 5.12 (d, *J* = 4.2 Hz, 32H, O*H*-2′), 4.89 (br s, 32H, O*H*-3), 4.79 (br s, 32H, O*H*-3′), 4.67 (t, *J* = 5.1 Hz, 64H, O*H*-6/6′), 4.54–4.51 (m, 96H, OCH$_2$C*H*$_2$,

O*H*-4′), 4.25 (d, *J* = 6.8 Hz, 32H, H-1′), 3.93 (br s, 32H, CH_2OC_q), 3.85 (ddd, *J* = 9.3, 8.5, 5.8 Hz, 32H, H-2), 3.77 (dd, *J* = 10.9, 4.6 Hz, 32H, H-6_a), 3.66–3.31 (m, 768H, H-3, H-4, H-5, H-6_b, H-2′, H-3′, H-4′, H-5′, H-$6'_{a,b}$, CH_2NH, OCH_2), 1.67 (br s, 32H, OCH_2CH_2CH_2), 1.31 (br s, 56H, $Si^{0,1,2}$CH_2CH_2), 0.54 (br s, 144H, SiCH_2), –0.06 (s, 96H, Si^3Me_2), –0.11 (s, 36H, $Si^{1,2}Me$). ^{13}C {^{1}H} NMR (101 MHz, dmso-d_6, HSQC, HMBC, HSQCTOCSY): δ 165.6 (*C*O), 158.5 (C_qO), 144.0 ($C_{q(Tria)}$), 135.8 ($C_{q(Ph)}$), 123.2 (CH_{Tria}), 118.4 (CH_{Ph}), 115.6 (2CH_{Ph}), 103.8 (C-1′), 86.9 (C-1), 79.8 (C-4), 77.7 (C-5), 75.6 (C-5′), 75.2 (C-3), 73.2 (C-3′), 71.8 (C-2), 70.53 (C-2′), 70.45 (*C*H_2OC_q), 69.67 (O*C*H_2), 69.65 (2×O*C*H_2), 69.6, 69.1, 68.8 (3×O*C*H_2), 68.2 (C-4′), 63.4 (OCH_2*C*H_2), 60.4 (C-6′), 60.0 (C-6), overlapped with dmso from HSQC 39.2 (*C*H_2NH), 23.3 (CH_2*C*H_2CH_2O), 19.4 (Si^2CH_2CH_2*C*$H_2$$Si^3$), not detected or overlapped ($Si^{0/1}$*C*H_2*C*H_2*C*$H_2$$Si^{1/2}$), 18.3 ($Si^2CH_2$*C*$H_2CH_2$$Si^3$), 18.1 ($Si^2$*C*$H_2CH_2CH_2$$Si^3$), 10.9 (*C*$H_2CH_2CH_2$O), –3.5 ($Si^3Me_2$), –4.9 ($Si^{1,2}Me$). ^{29}Si INEPT NMR (79 MHz, dmso-d_6): δ 2.14 (Si^3), 1.08 ($Si^{1,2}$), not detected (Si^0).

5. NMR spectra

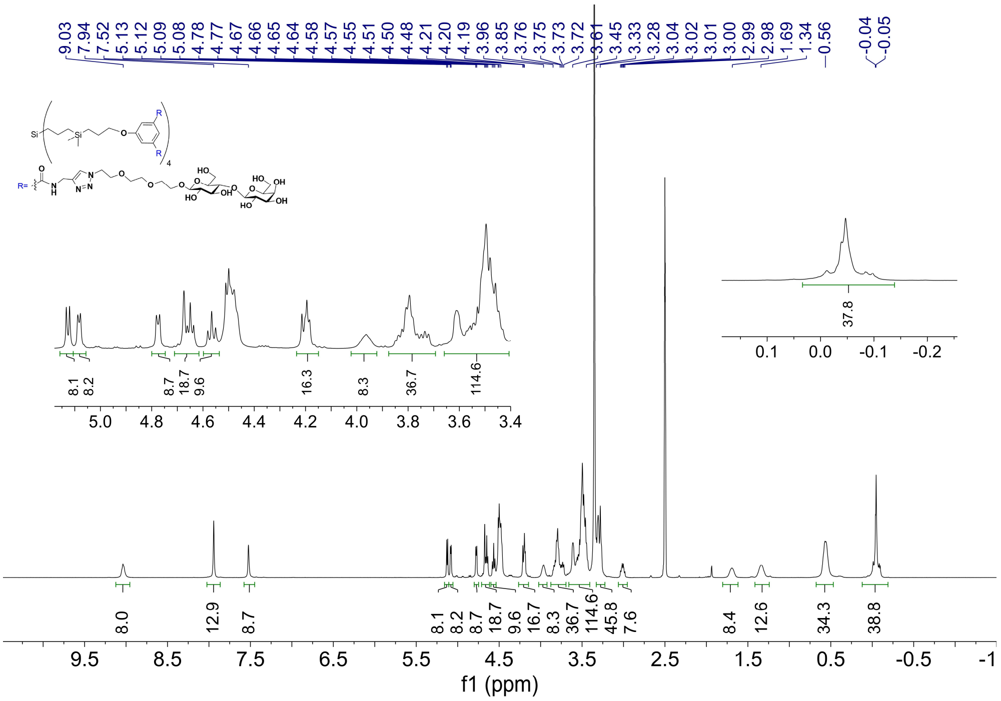

Figure S2: ^{1}H NMR (400 MHz, dmso-d_6) of **G$_1$-A-Lac$_8$ (12b)**.

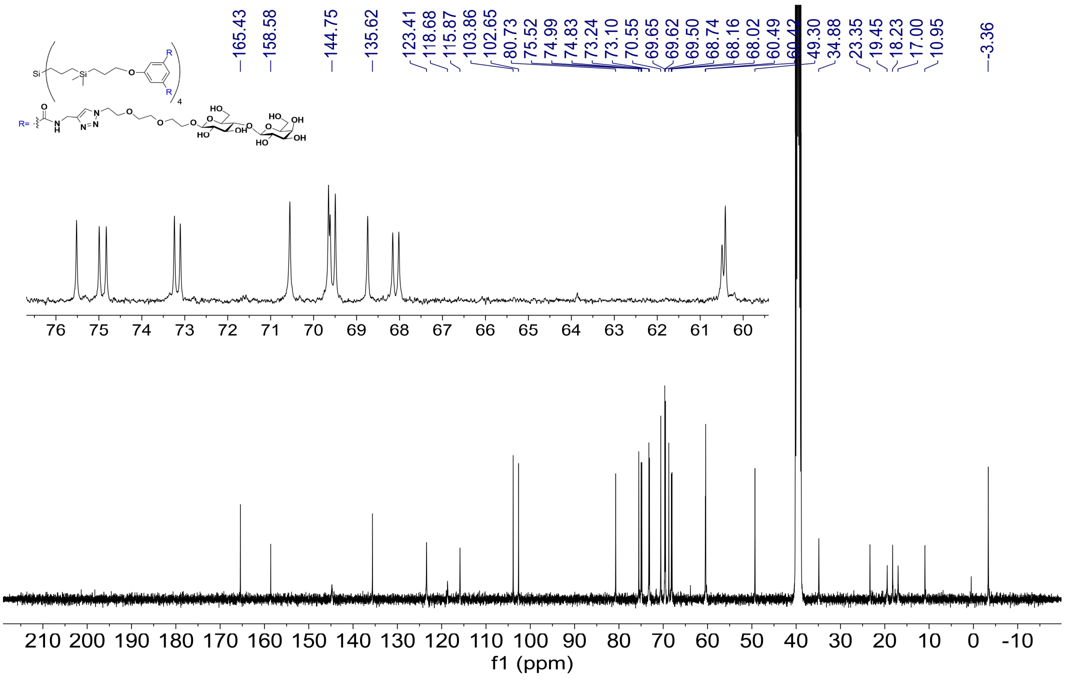

Figure S3: ^{13}C{^{1}H} NMR (400 MHz, dmso-d_6) of **G$_1$-A-Lac$_8$ (12b)**.

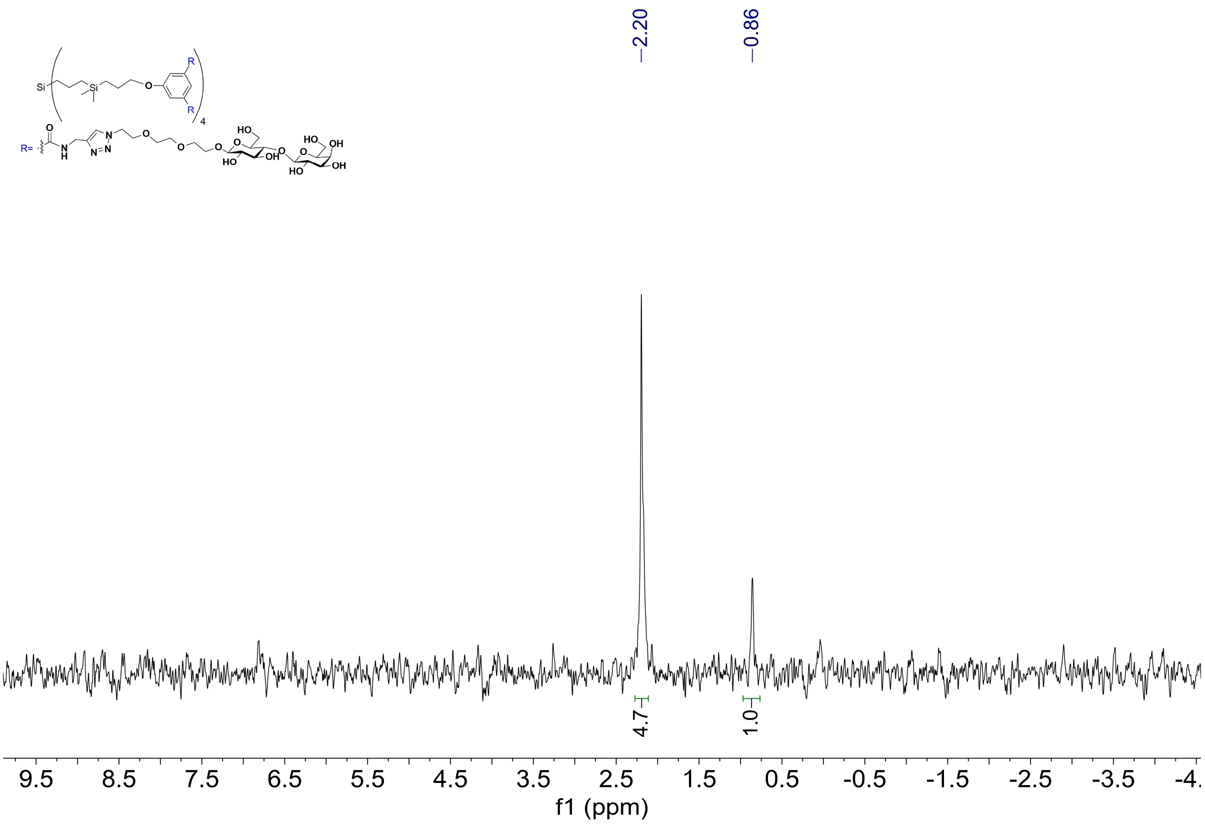

Figure S4: ^{29}Si INEPT NMR (400 MHz, dmso-d_6) of **G$_1$-A-Lac$_8$ (12b)**.

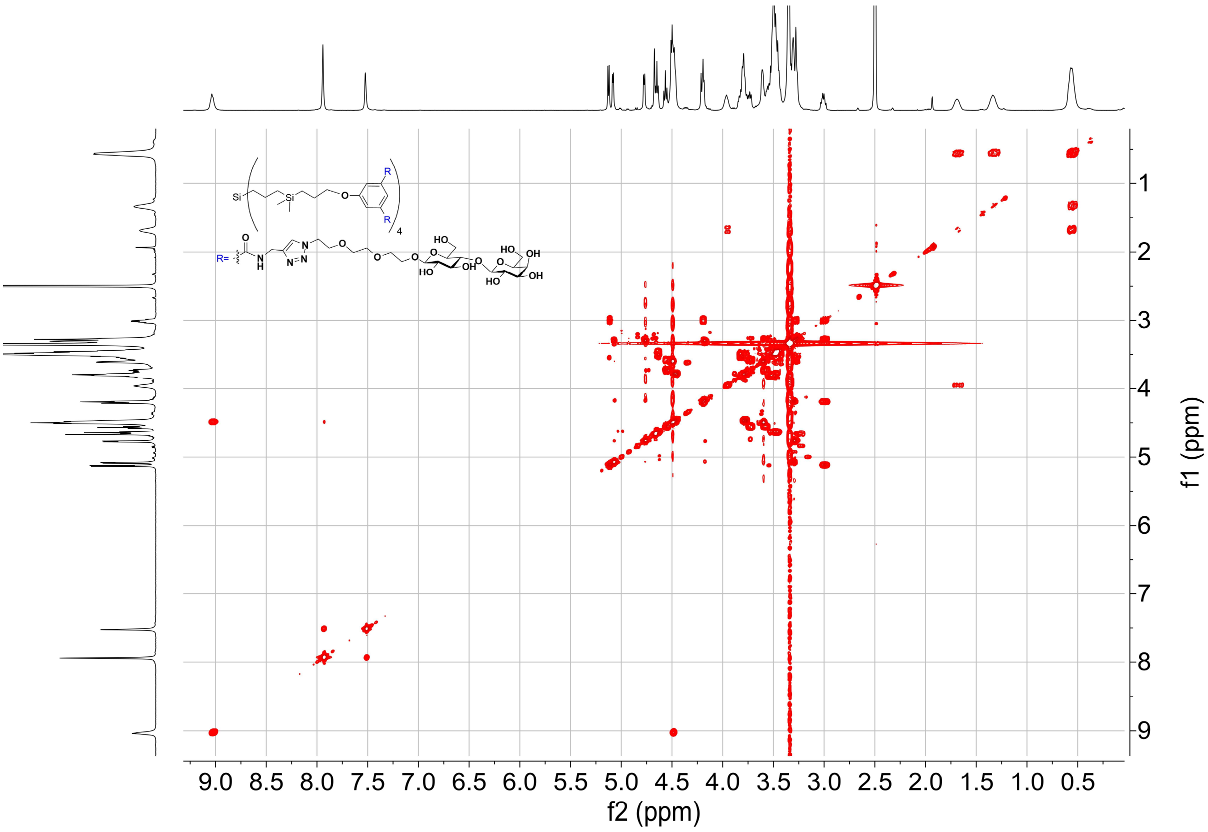

Figure S5: ^{1}H-^{1}H COSY NMR (400 MHz, dmso-d_6) of **G$_1$-A-Lac$_8$ (12b)**.

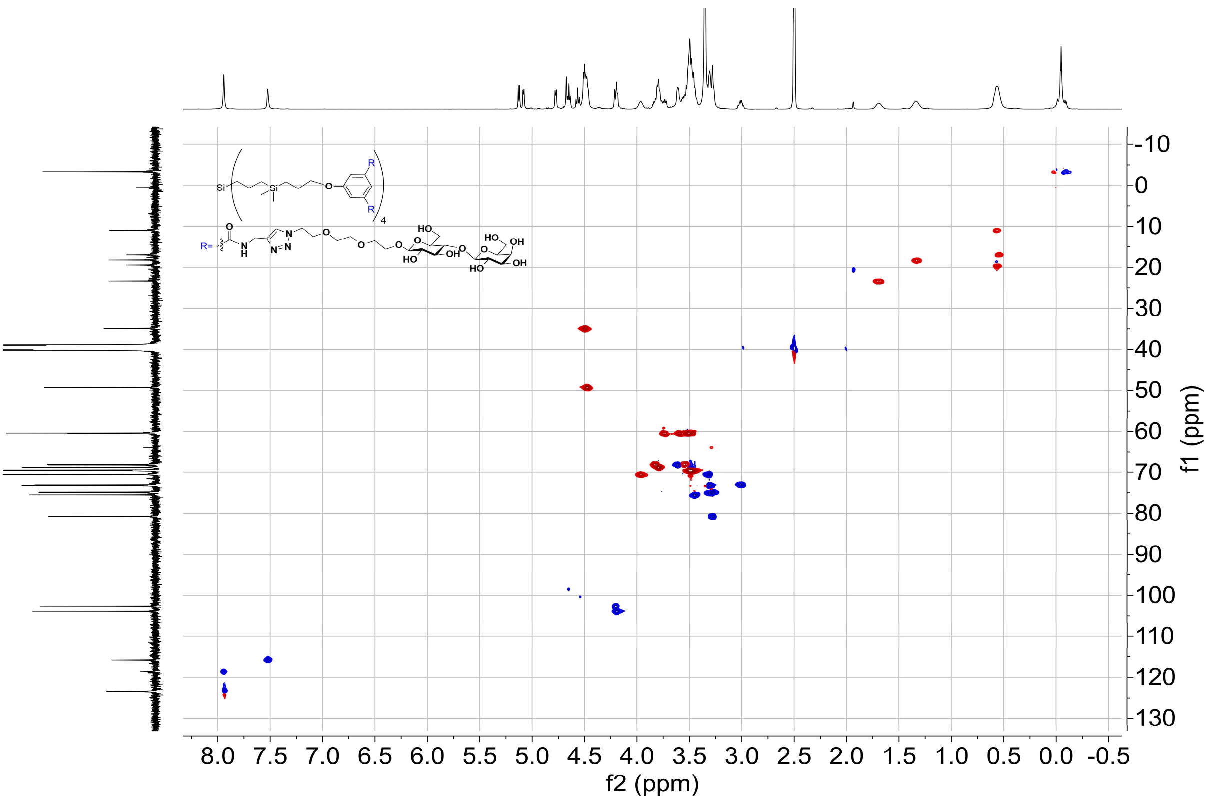

Figure S6: ^{1}H-^{13}C HSQC NMR (400 MHz, dmso-d_6) of **G$_1$-A-Lac$_8$ (12b)**.

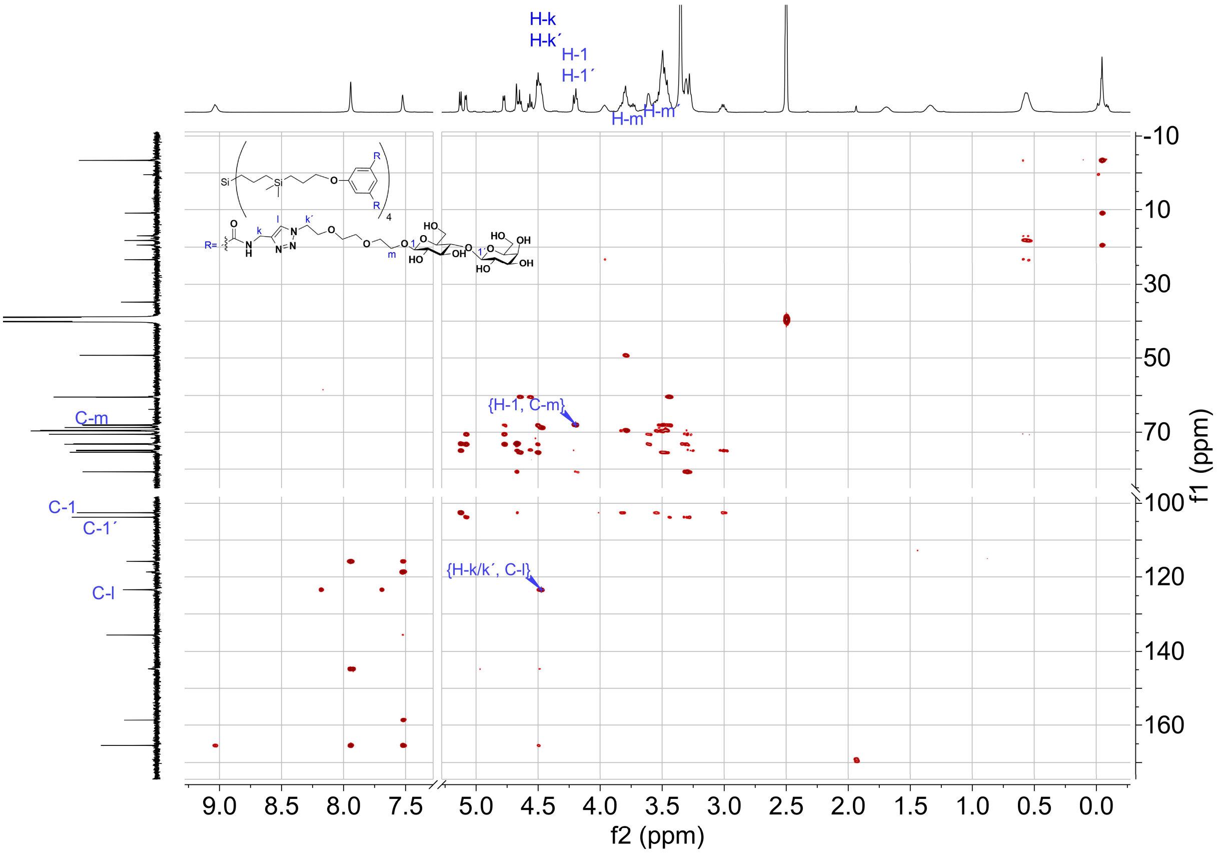

Figure S7: ^{1}H-^{13}C HMBC NMR (400 MHz, dmso-d_6) of **G$_1$-A-Lac$_8$ (12b)**.

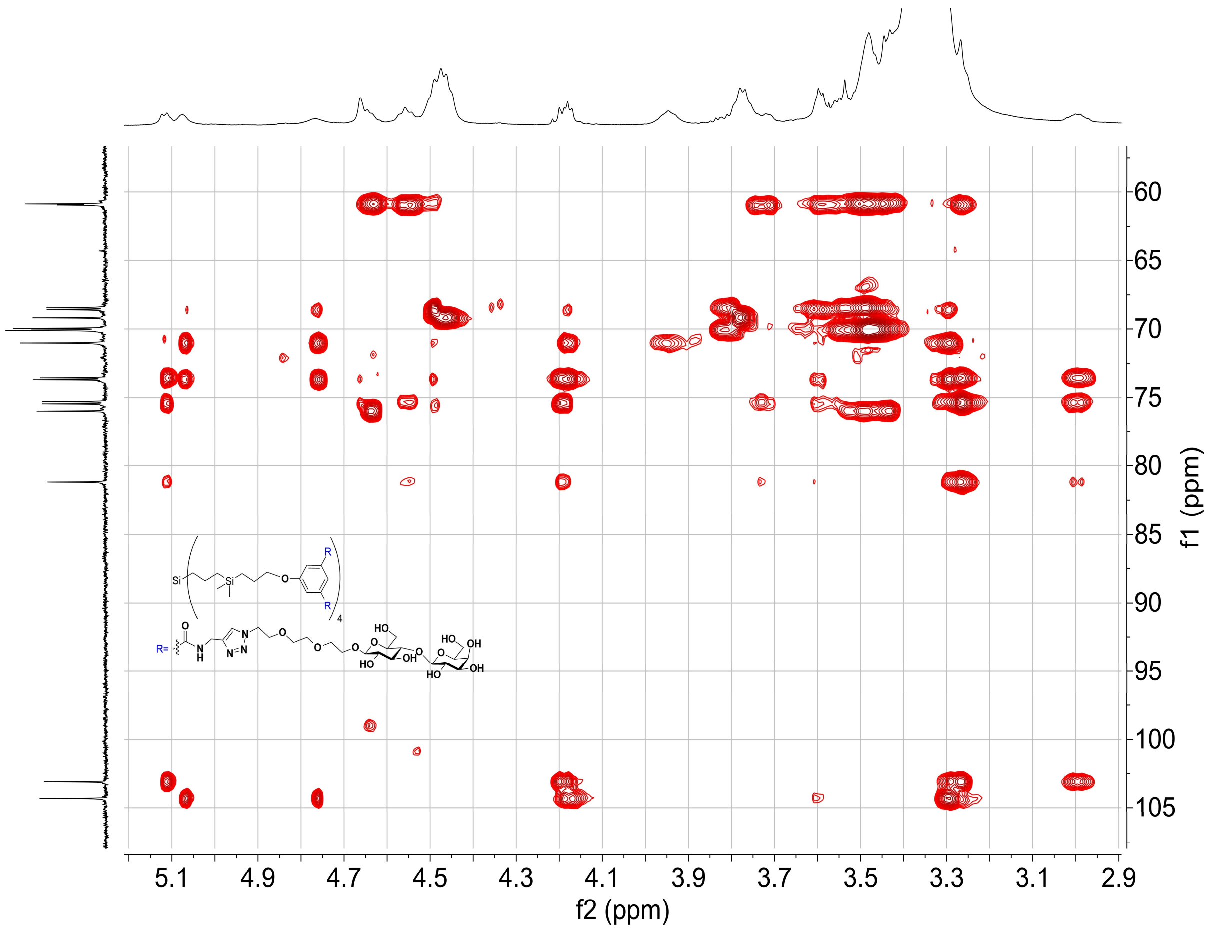

Figure S8: ^{1}H-^{13}C HSQC TOCSY NMR (400 MHz, dmso-*d*$_6$) of **G$_1$-A-Lac$_8$ (12b)**.

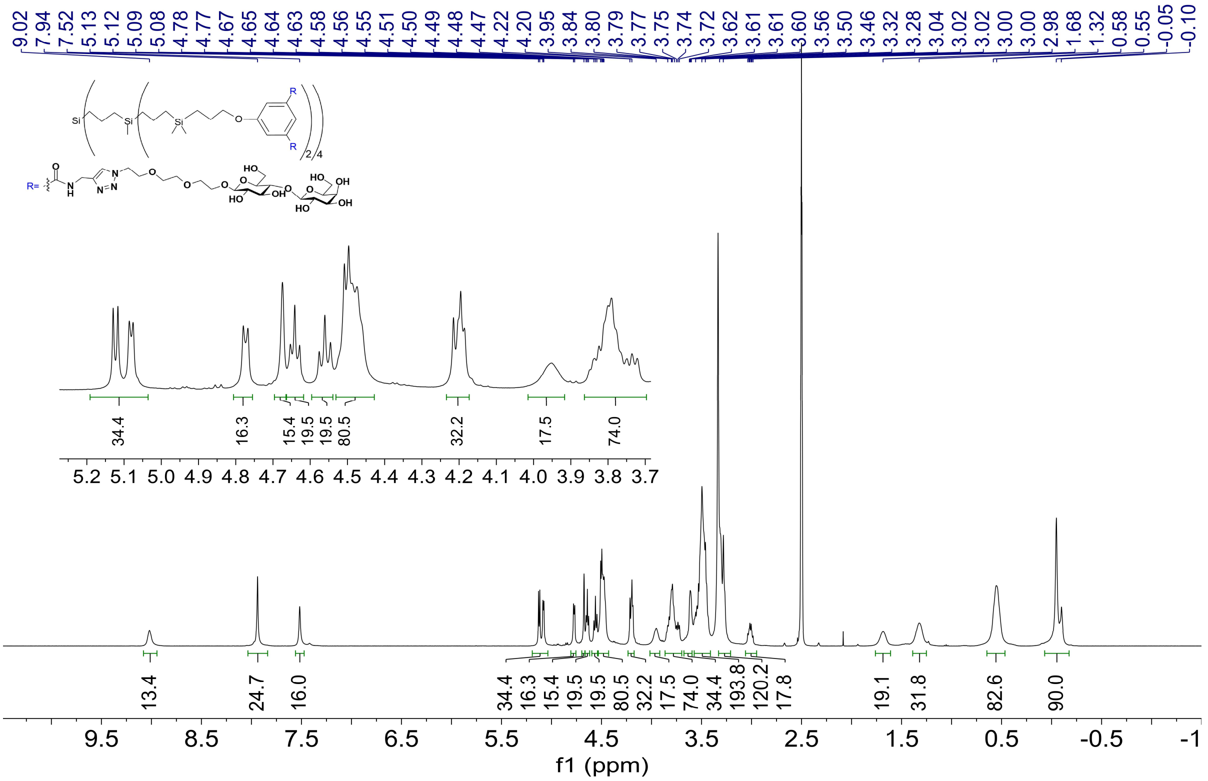

Figure S9: ^{1}H NMR (400 MHz, dmso-*d*$_6$) of **G$_2$-A-Lac$_{16}$ (13b)**.

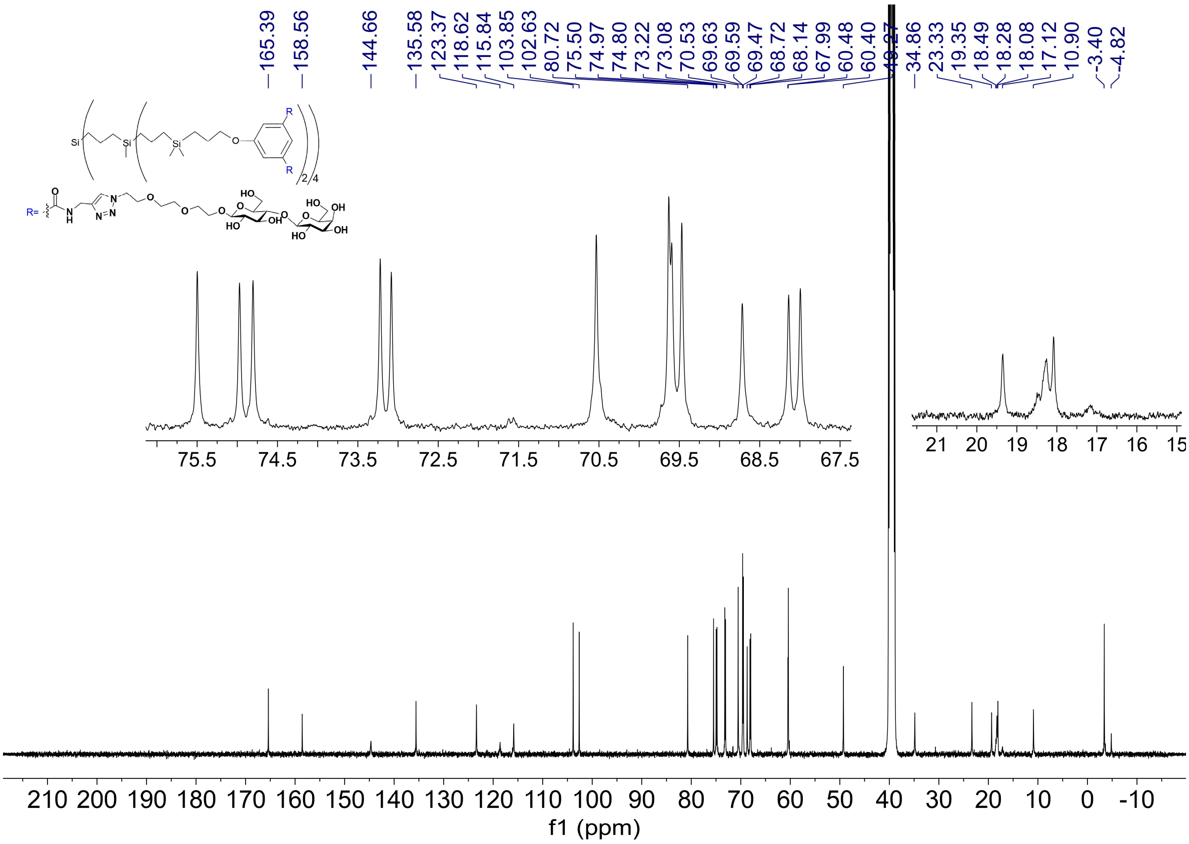

Figure S10: $^{13}C\{^{1}H\}$ NMR (400 MHz, dmso-d_6) of **G2-A-Lac16 (13b)**.

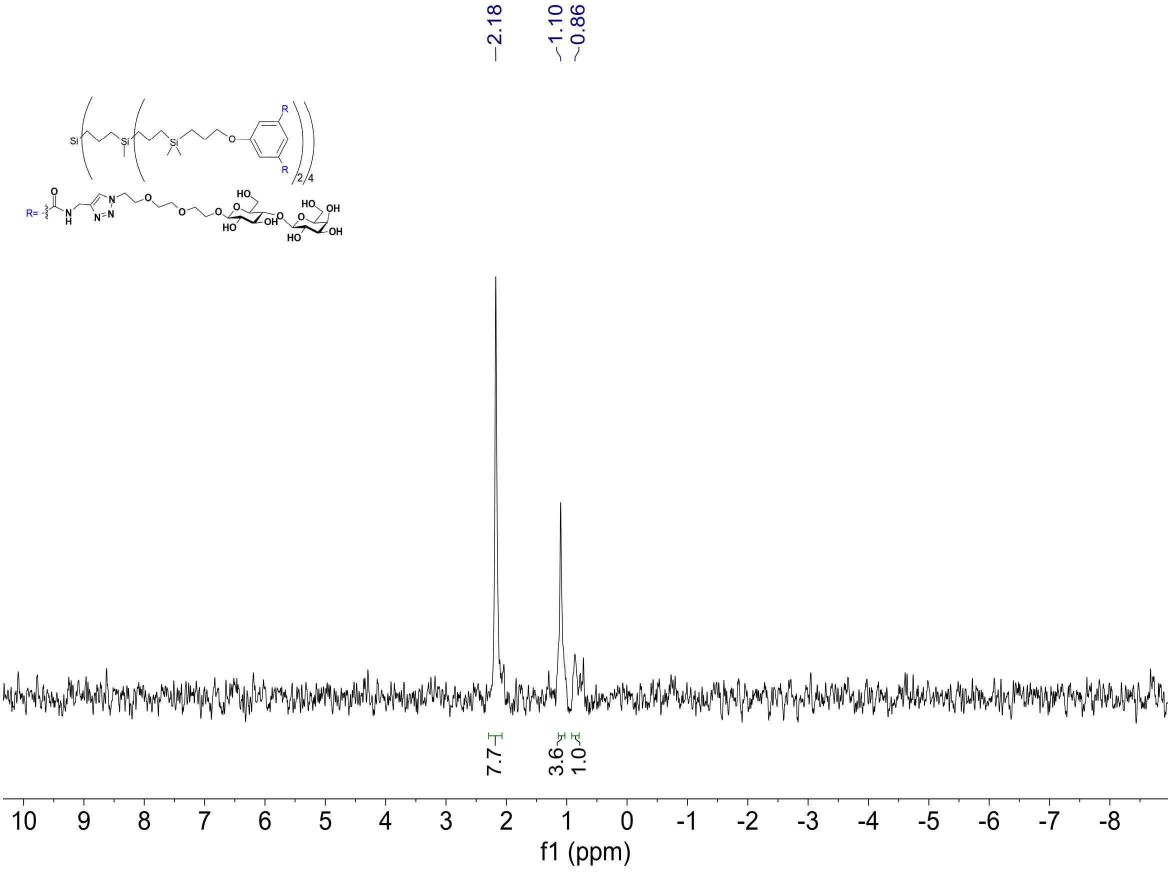

Figure S11: ^{29}Si INEPT NMR (400 MHz, dmso-d_6) of **G2-A-Lac16 (13b)**.

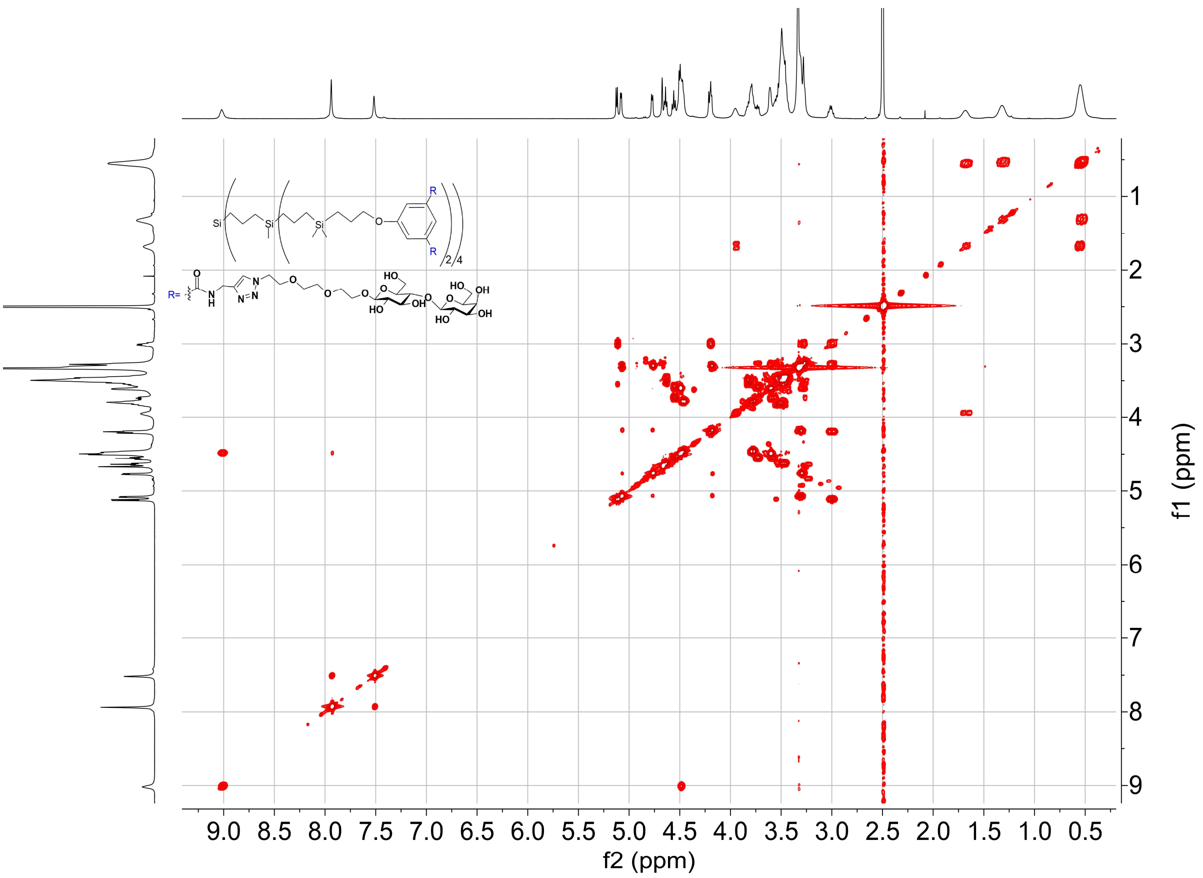

Figure S12: ^{1}H-^{1}H COSY NMR (400 MHz, dmso-d_6) of **G$_2$-A-Lac$_{16}$ (13b)**.

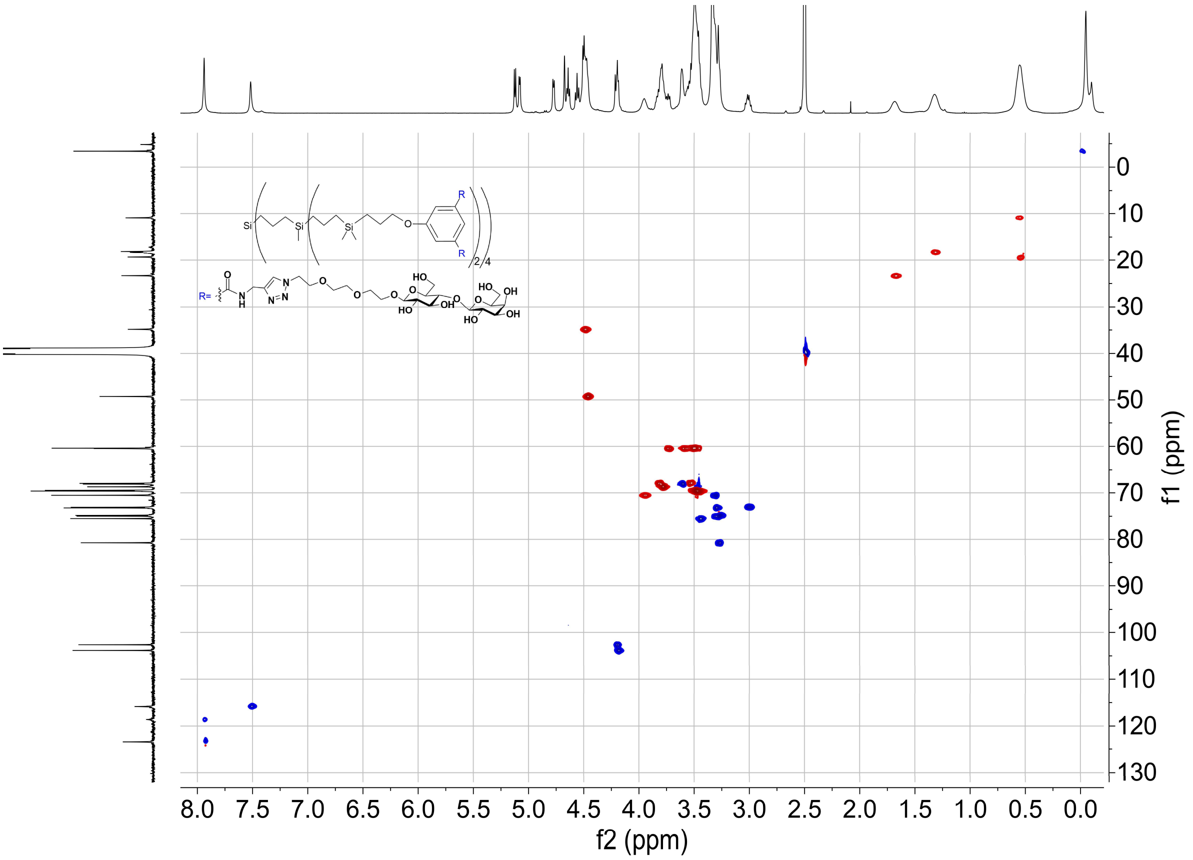

Figure S13: ^{1}H-^{13}C HSQC NMR (400 MHz, dmso-d_6) of **G$_2$-A-Lac$_{16}$ (13b)**.

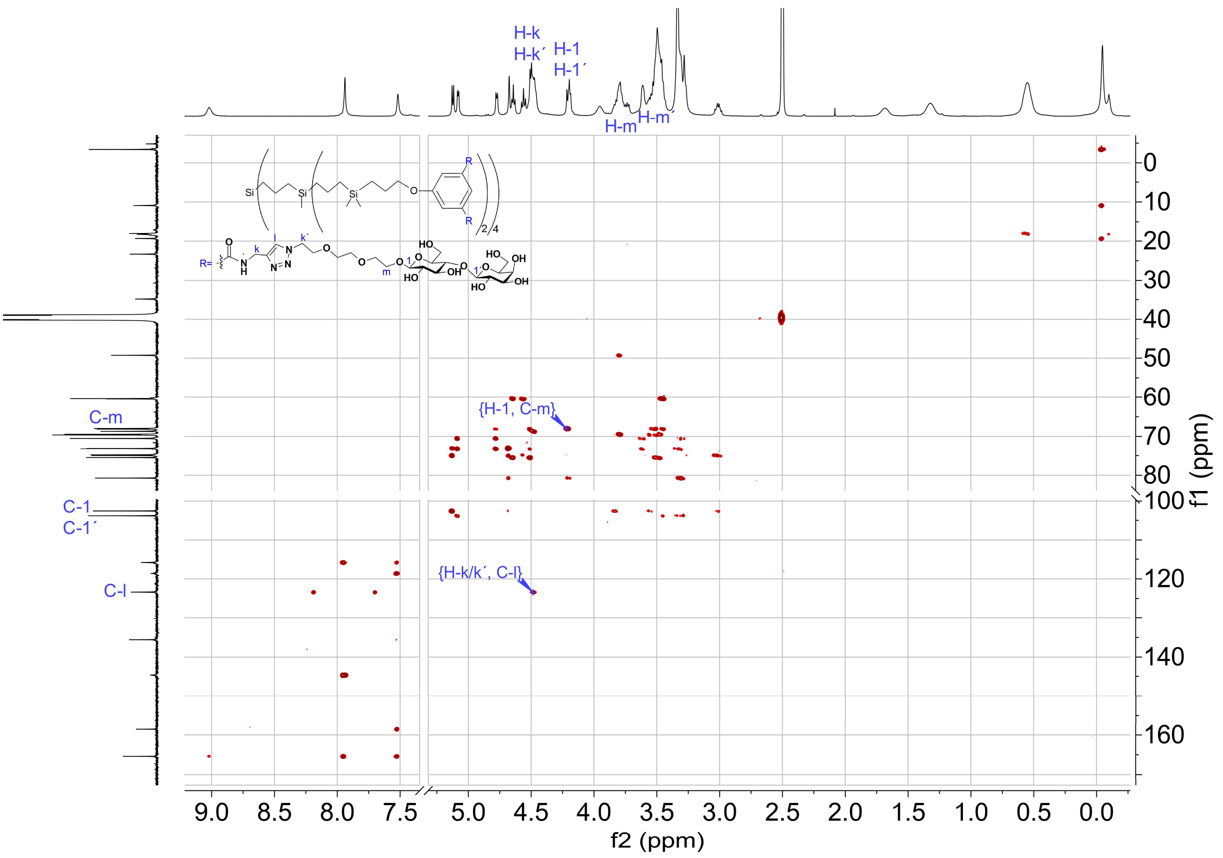

Figure S14: ^{1}H-^{13}C HMBC NMR (400 MHz, dmso-d_6) of **G$_2$-A-Lac$_{16}$ (13b)**.

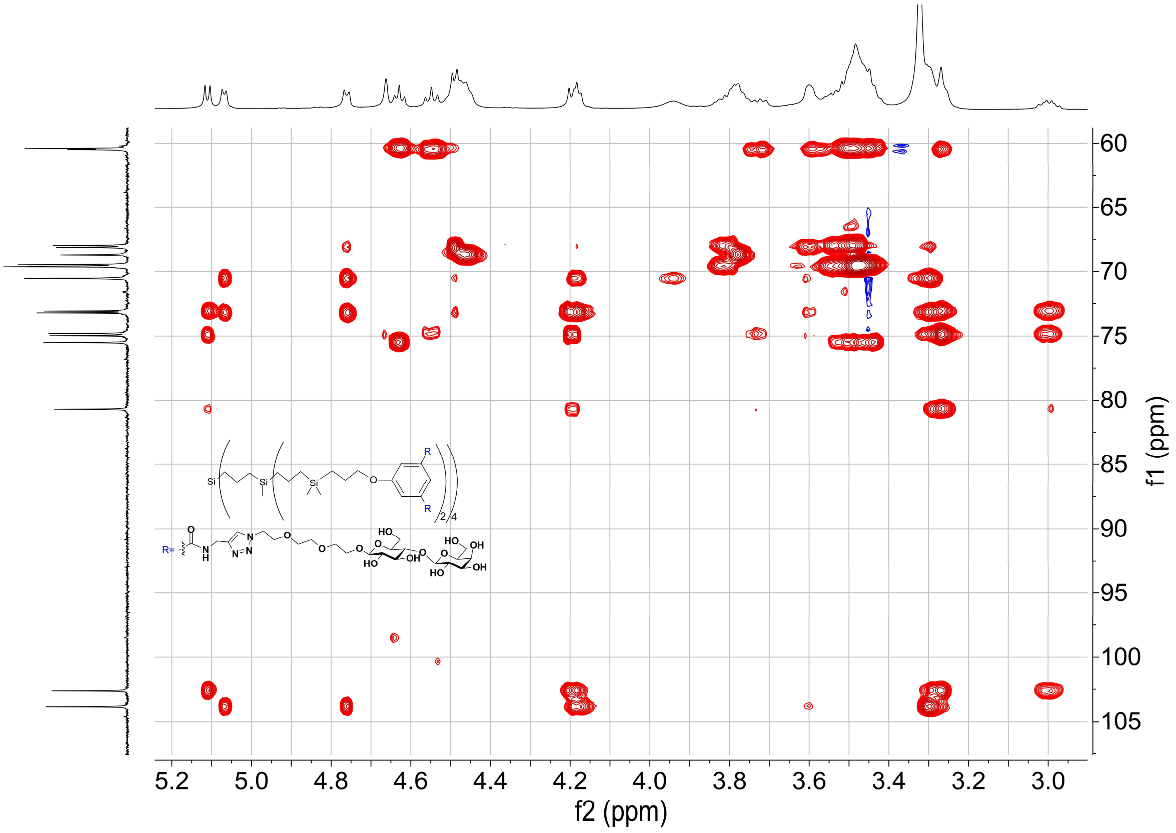

Figure S15: ^{1}H-^{13}C HSQC TOCSY NMR (400 MHz, dmso-d_6) of **G$_2$-A-Lac$_{16}$ (13b)**.

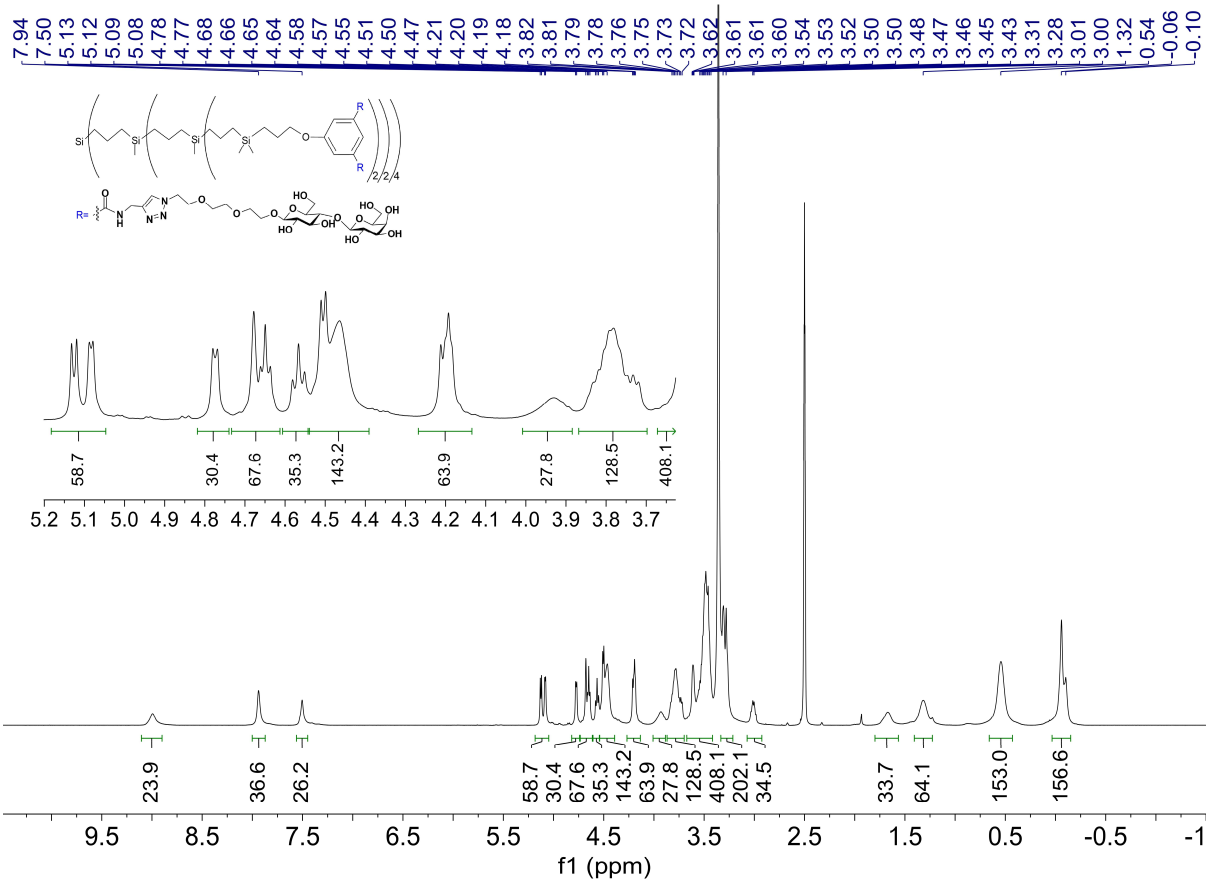

Figure S16: ^{1}H NMR (400 MHz, dmso-d_6) of **G$_3$-A-Lac$_{32}$ (14b)**.

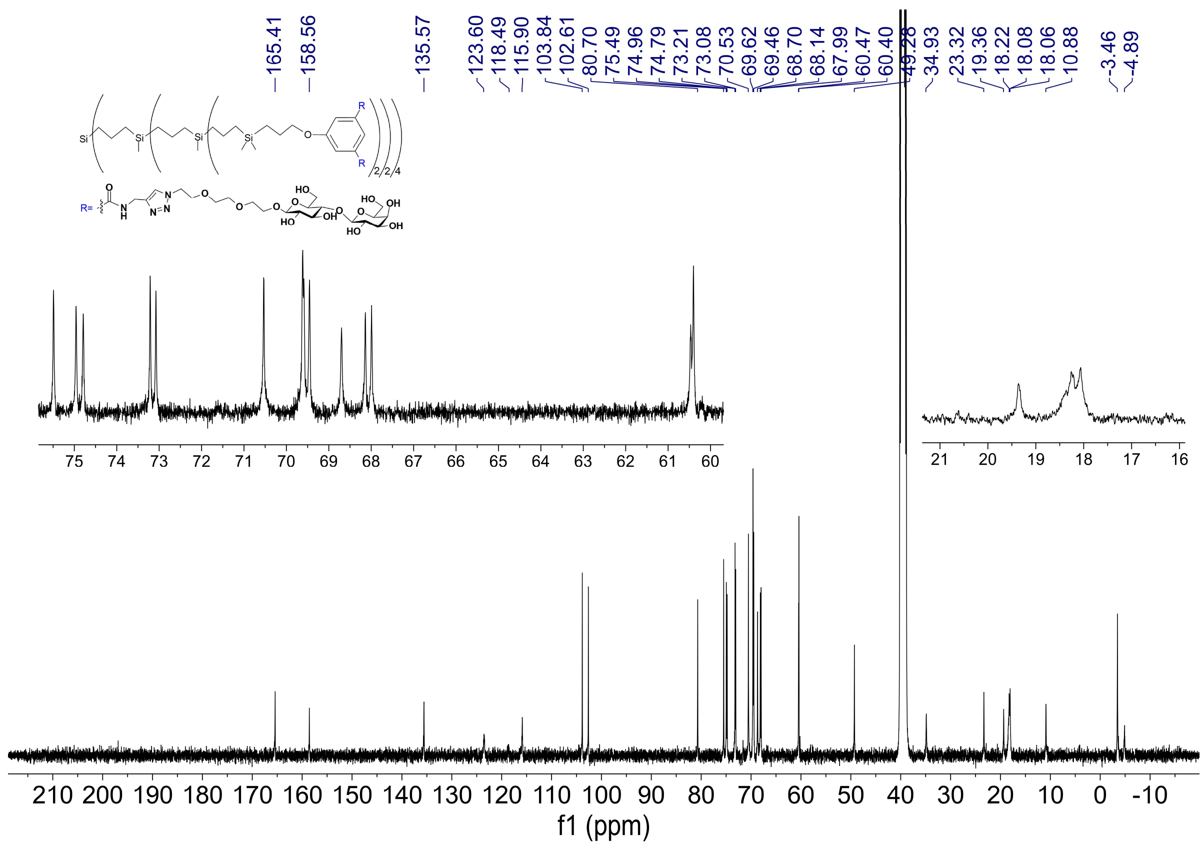

Figure S17: ^{13}C{^{1}H} NMR (400 MHz, dmso-d_6) of **G$_3$-A-Lac$_{32}$ (14b)**.

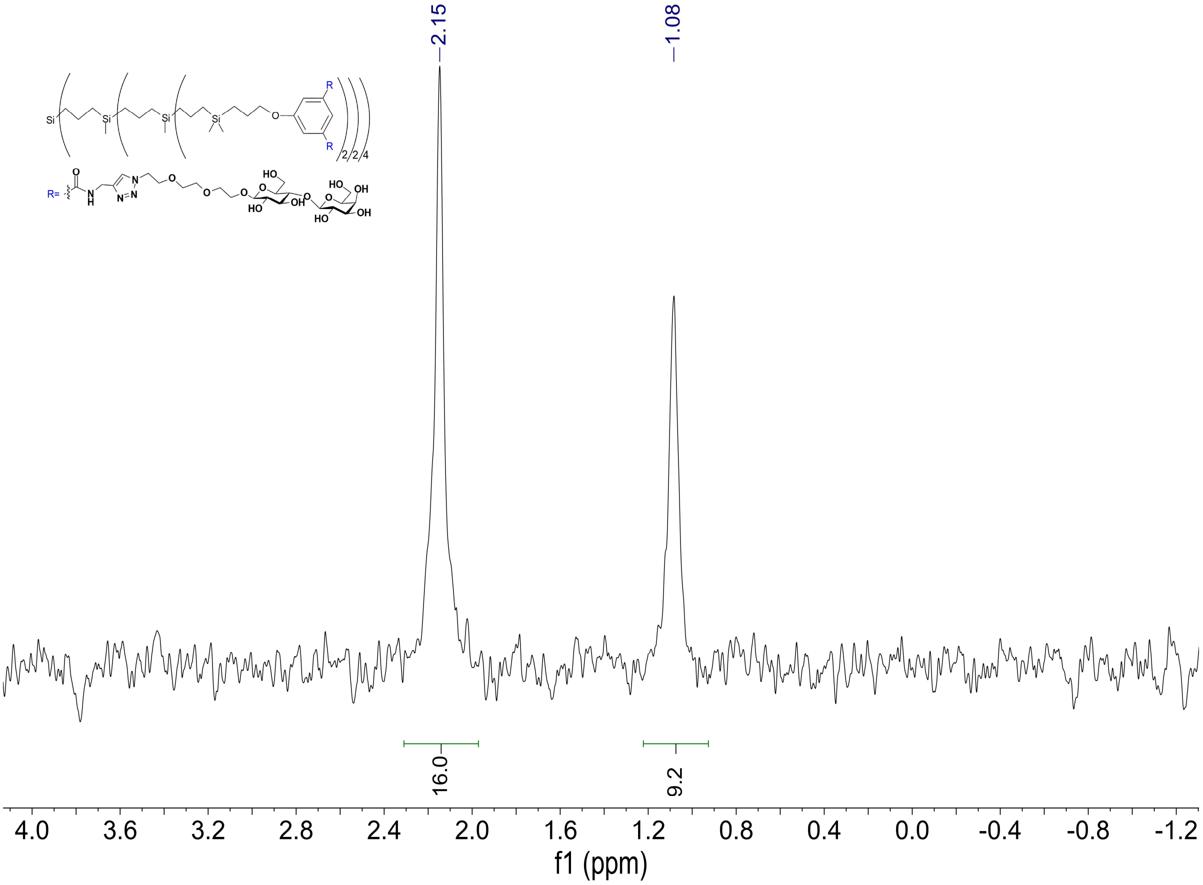

Figure S18: ^{29}Si INEPT NMR (400 MHz, dmso-d_6) of **G$_3$-A-Lac$_{32}$ (14b)**.

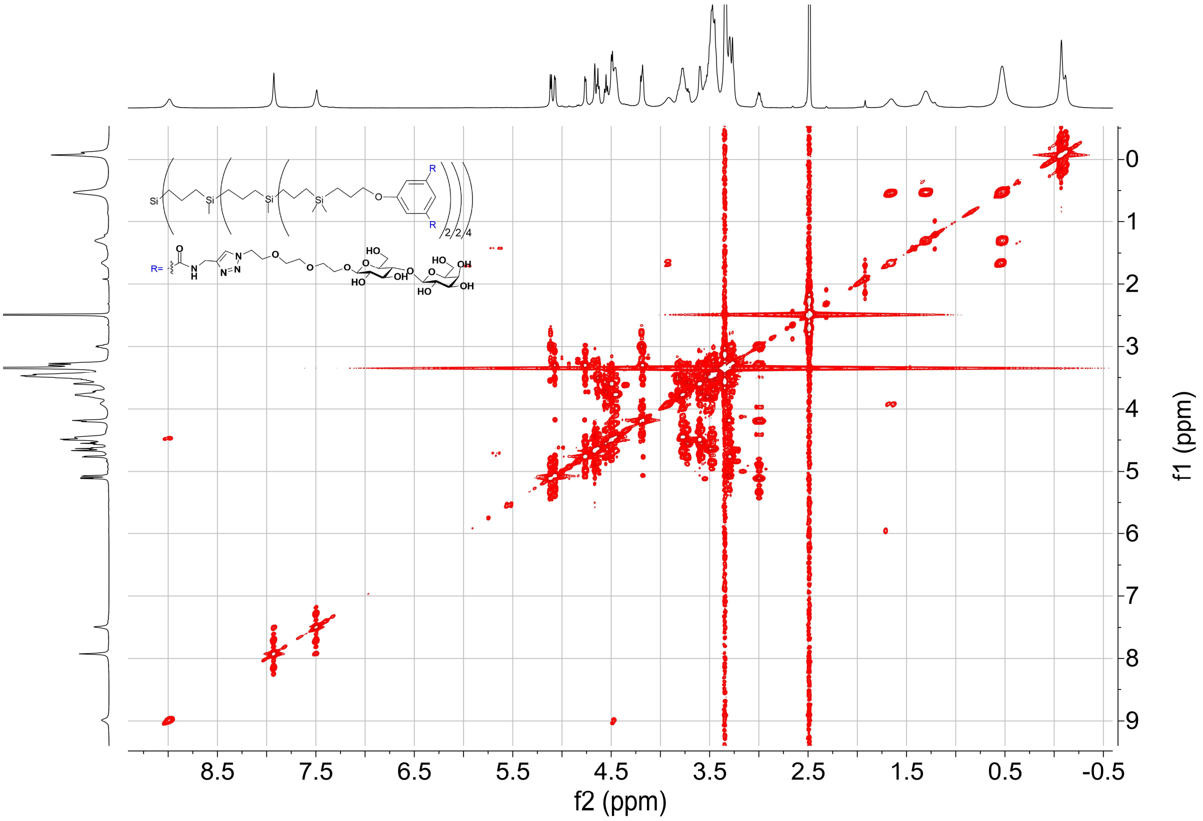

Figure S19: ^{1}H-^{1}H COSY NMR (400 MHz, dmso-d_6) of **G$_3$-A-Lac$_{32}$ (14b)**.

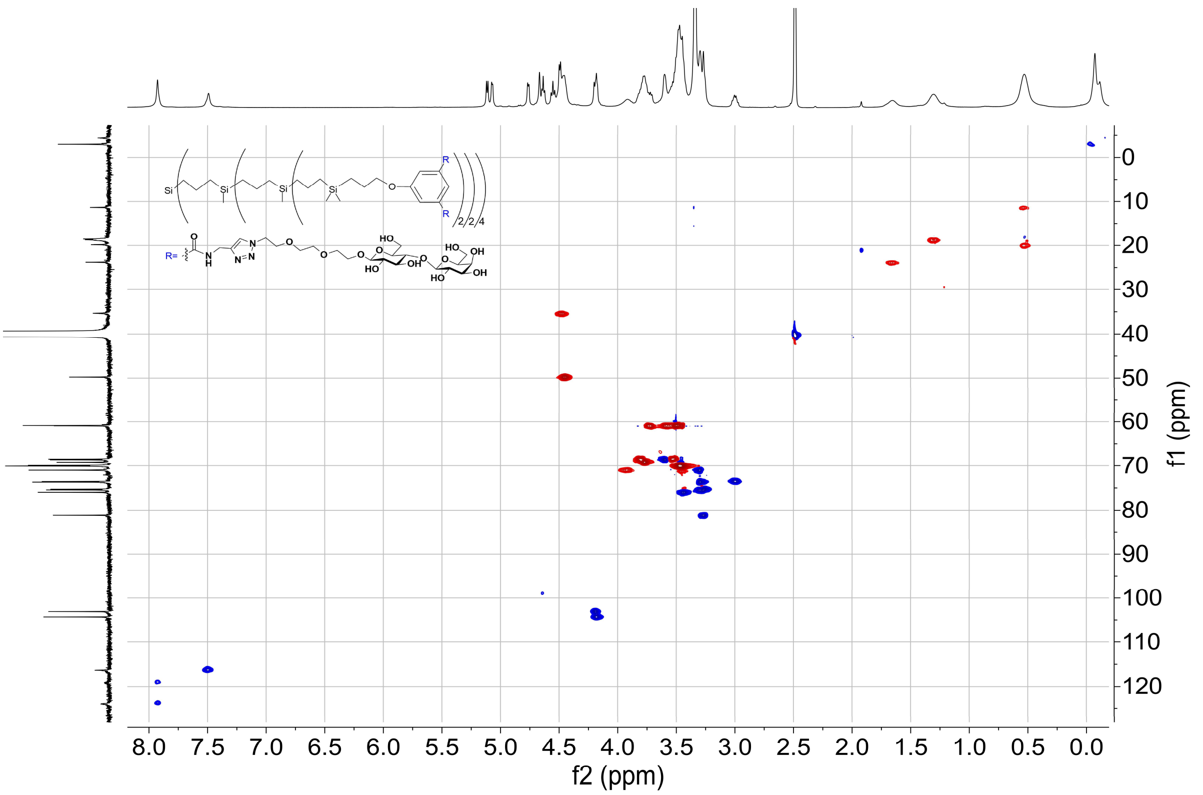

Figure S20: ^{1}H-^{13}C HSQC NMR (400 MHz, dmso-d_6) of **G$_3$-A-Lac$_{32}$ (14b)**.

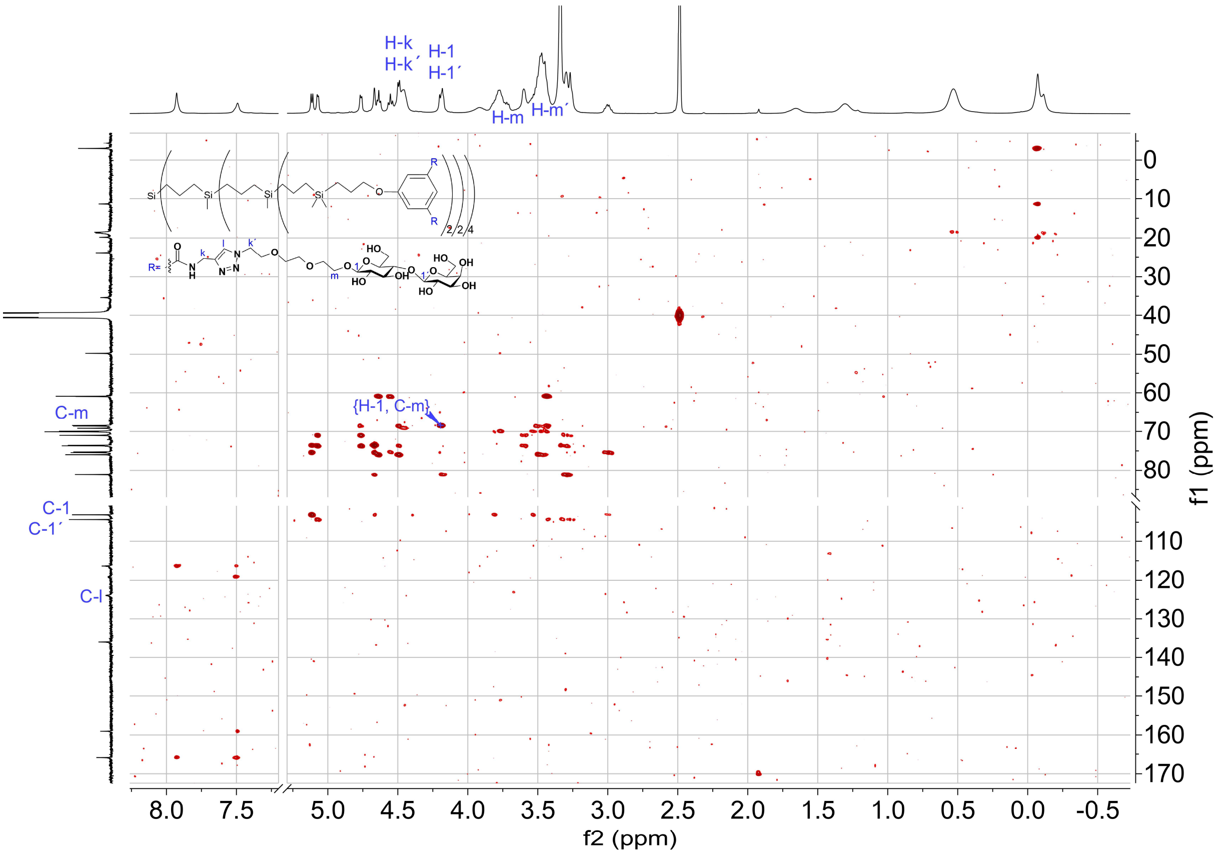

Figure S21: ^{1}H-^{13}C HMBC NMR (400 MHz, dmso-d_6) of **G$_3$-A-Lac$_{32}$ (14b)**.

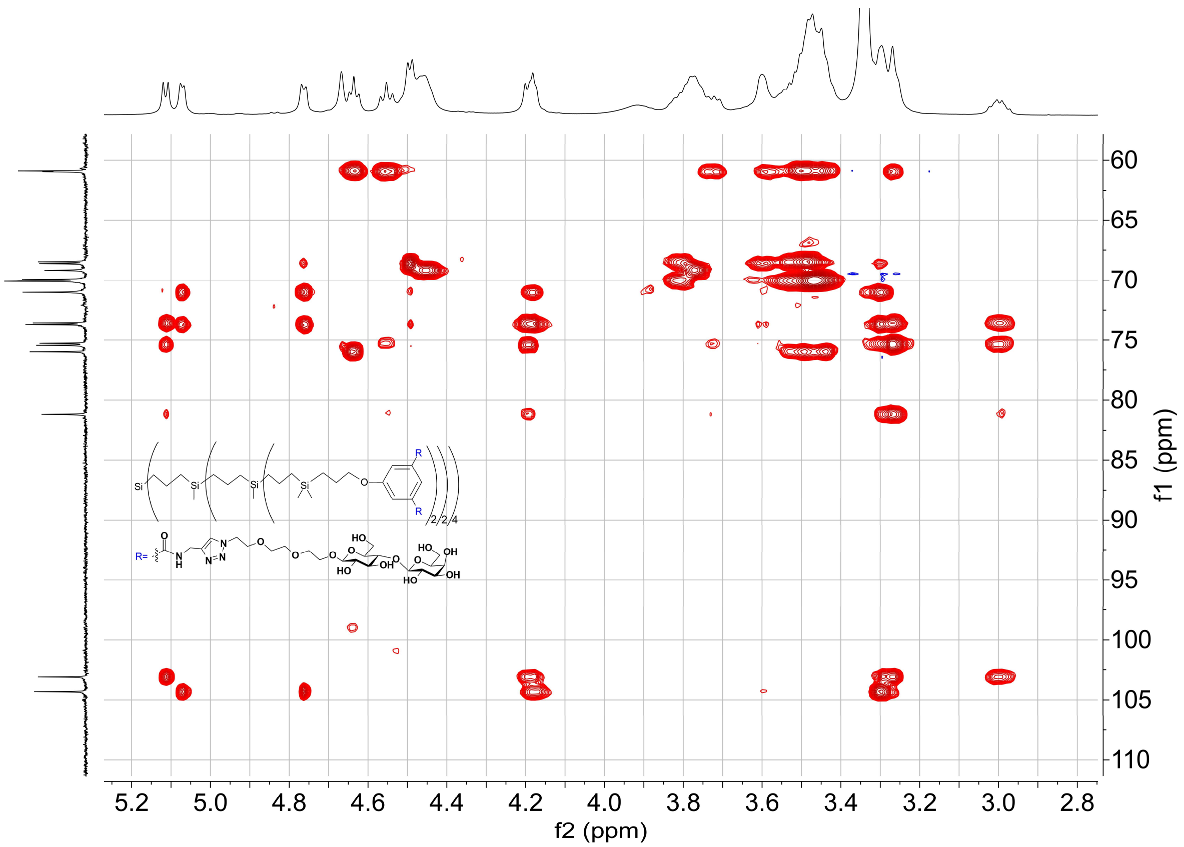

Figure S22: ^{1}H-^{13}C HSQC TOCSY NMR (400 MHz, dmso-d_6) of **G$_3$-A-Lac$_{32}$ (14b)**.

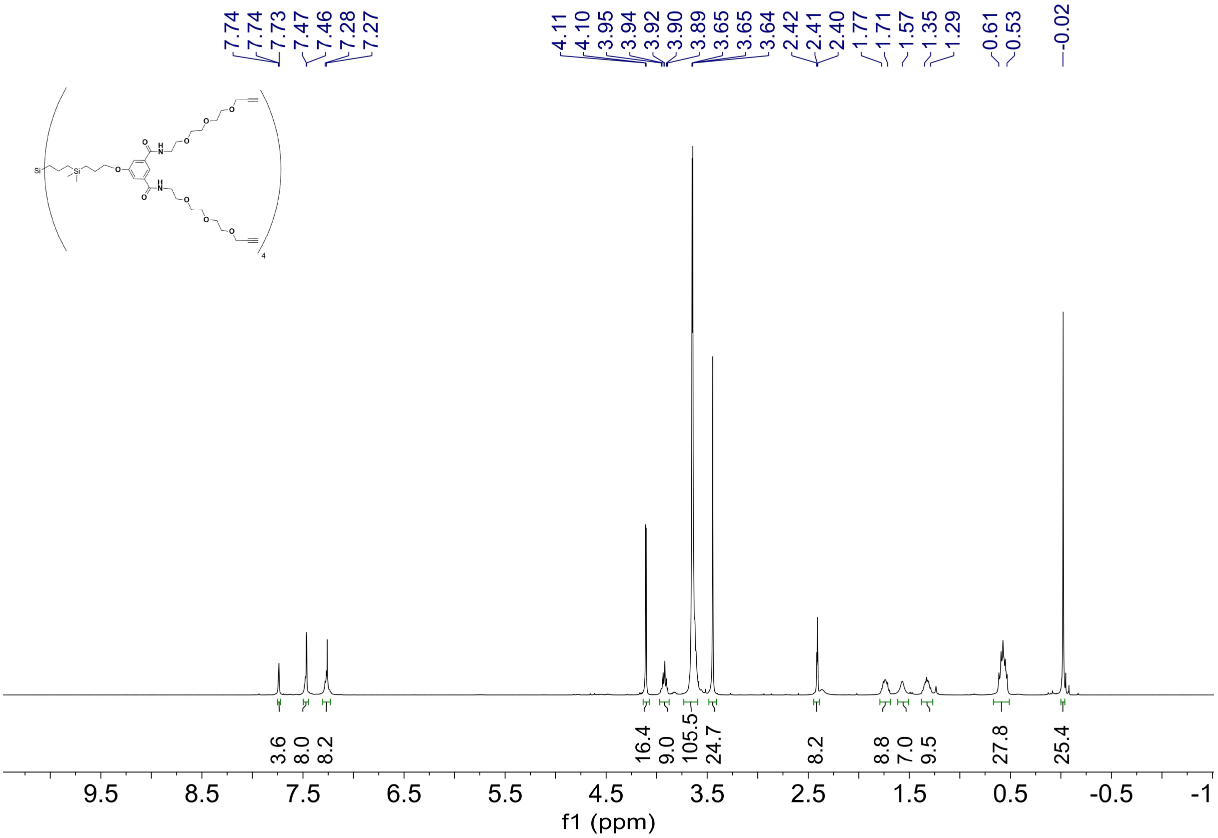

Figure S23: ^{1}H NMR (400 MHz, $CDCl_3$) of **G$_1$-B (9)**.

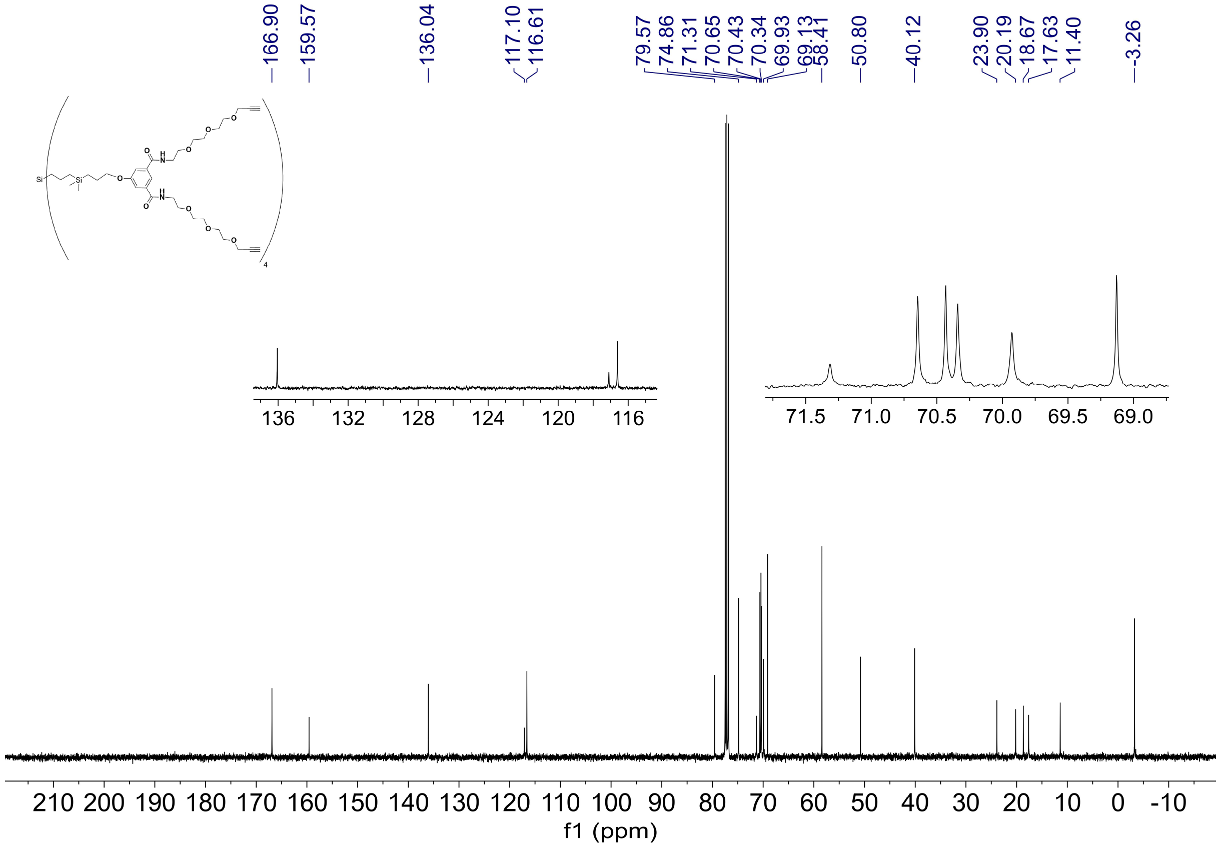

Figure S24: $^{13}C\{^{1}H\}$ NMR (400 MHz, $CDCl_3$) of **G_1-B (9)**.

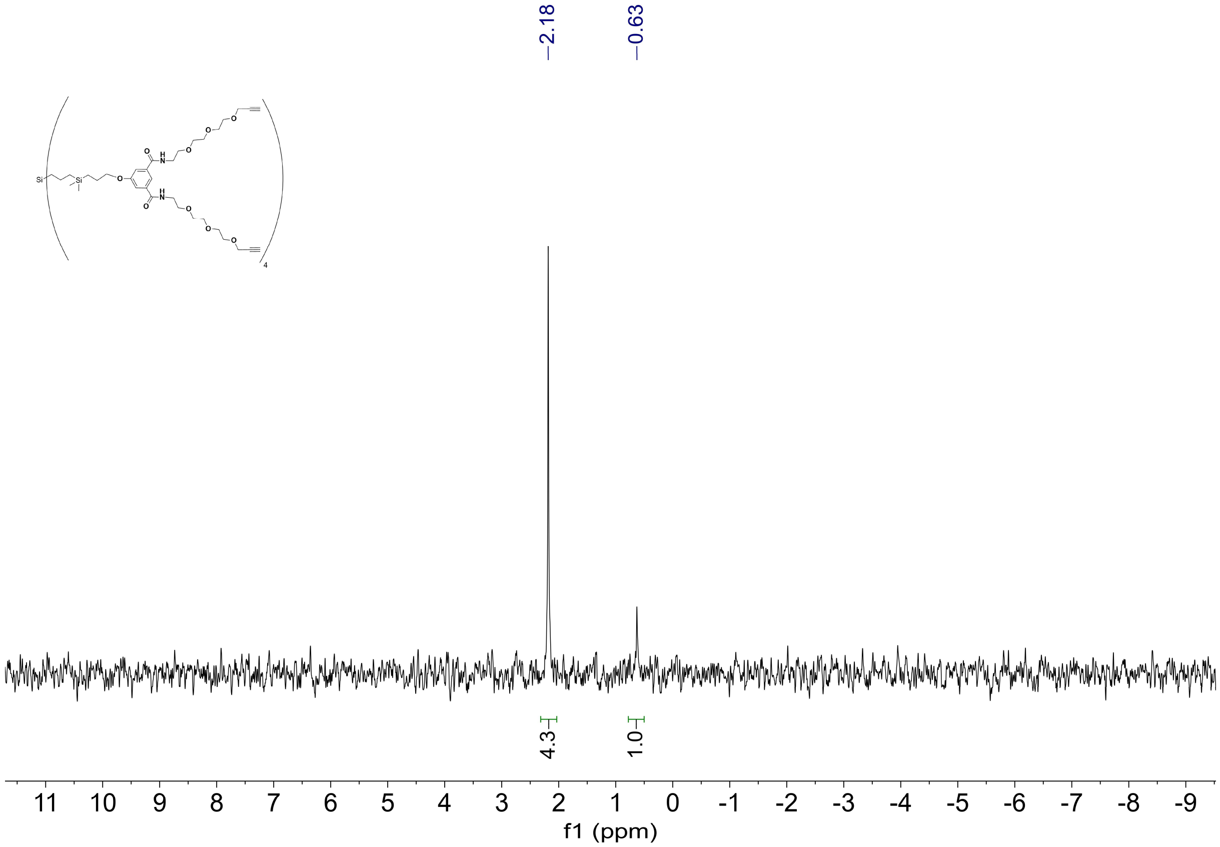

Figure S25: ^{29}Si INEPT NMR (400 MHz, $CDCl_3$) of **G_1-B (9)**.

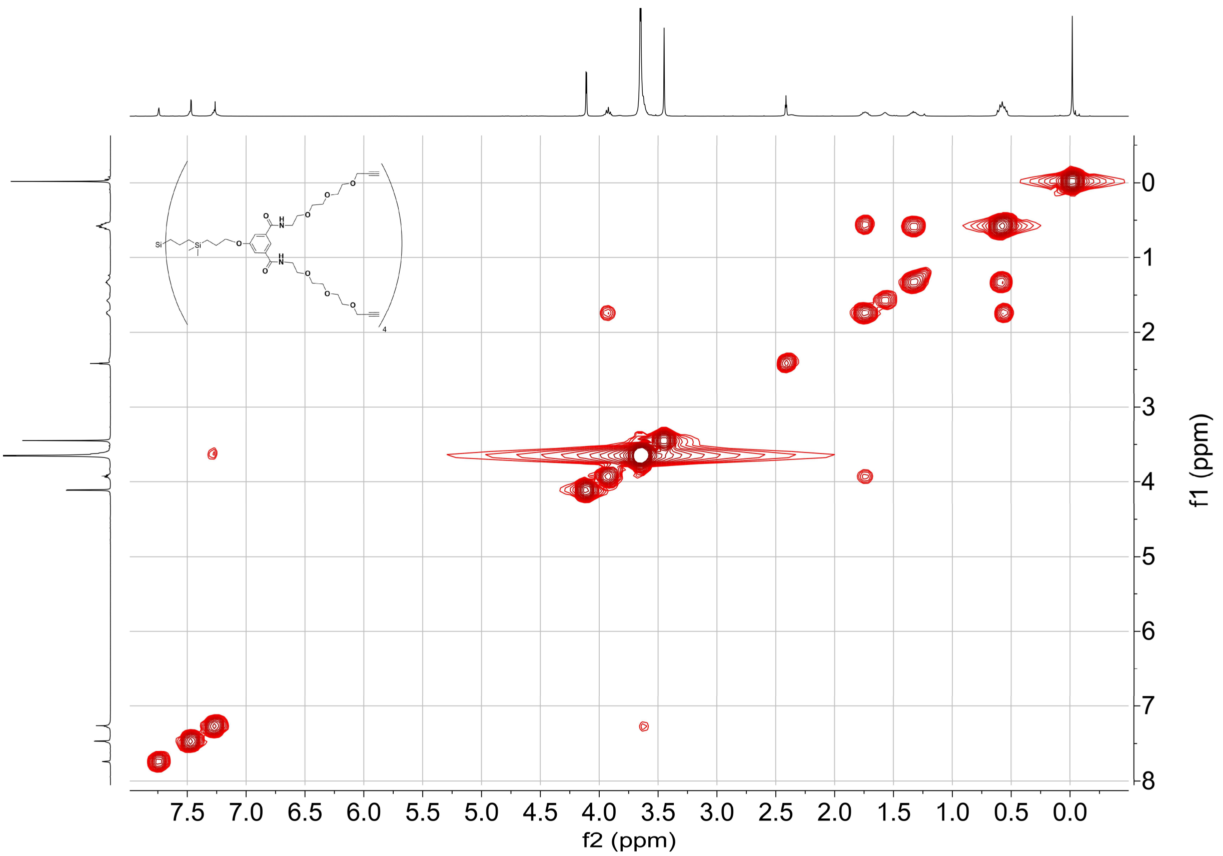

Figure S26: ^{1}H-^{1}H COSY NMR (400 MHz, $CDCl_3$) of **G_1-B (9)**.

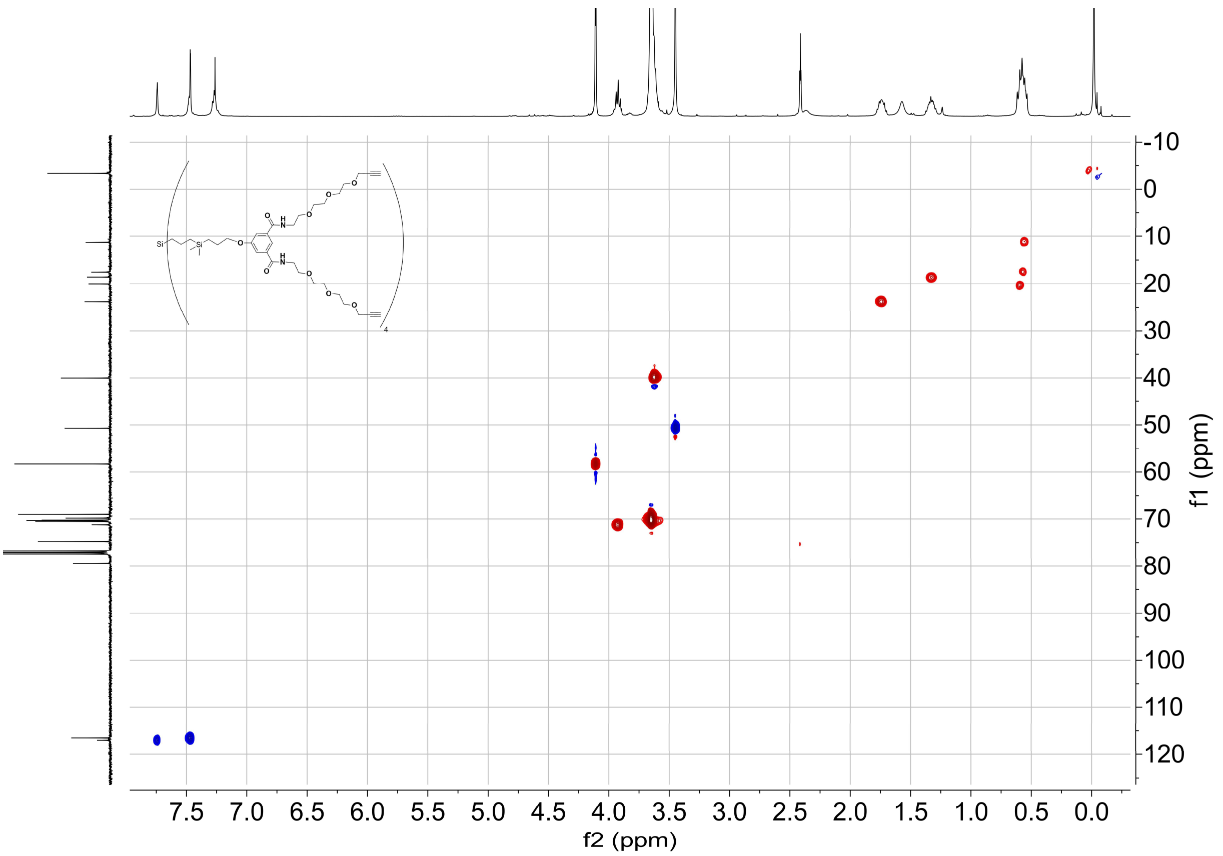

Figure S27: ^{1}H-^{13}C HSQC NMR (400 MHz, $CDCl_3$) of **G_1-B (9)**.

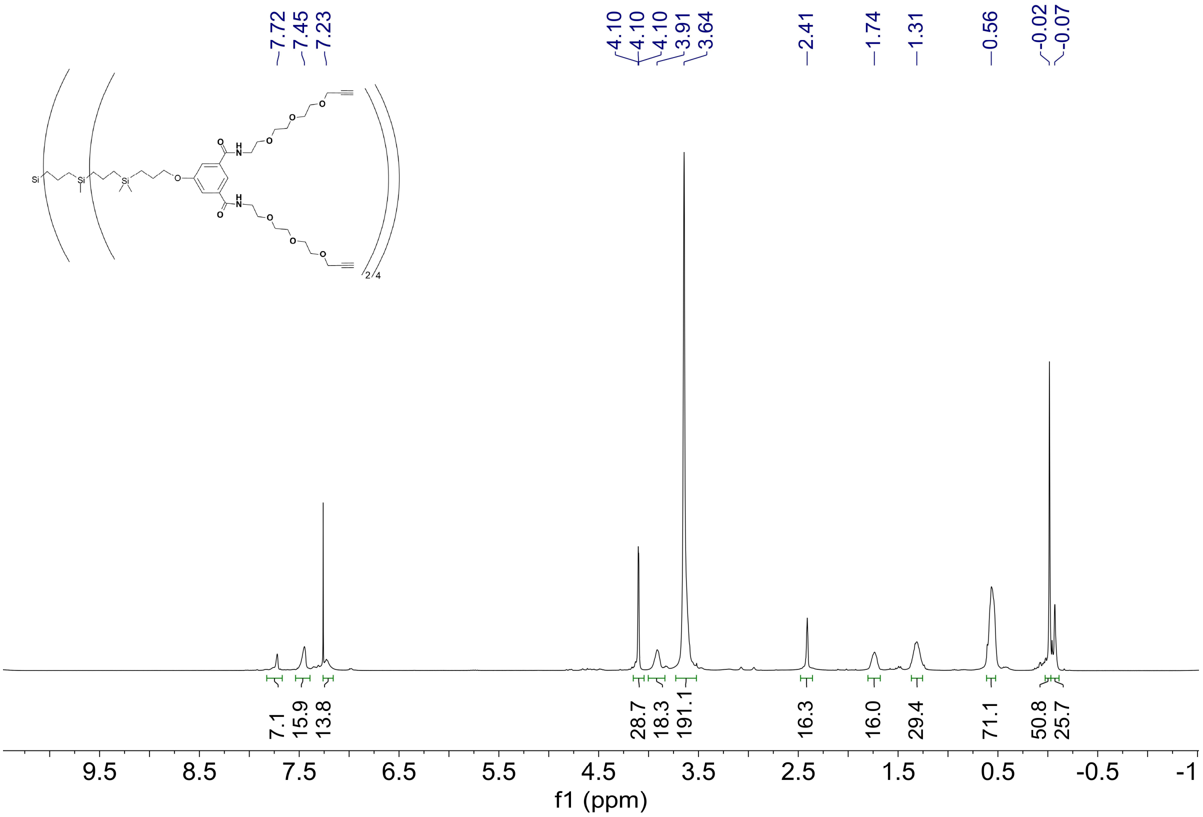

Figure S28: ^{1}H NMR (400 MHz, $CDCl_3$) of **G_2-B (10)**.

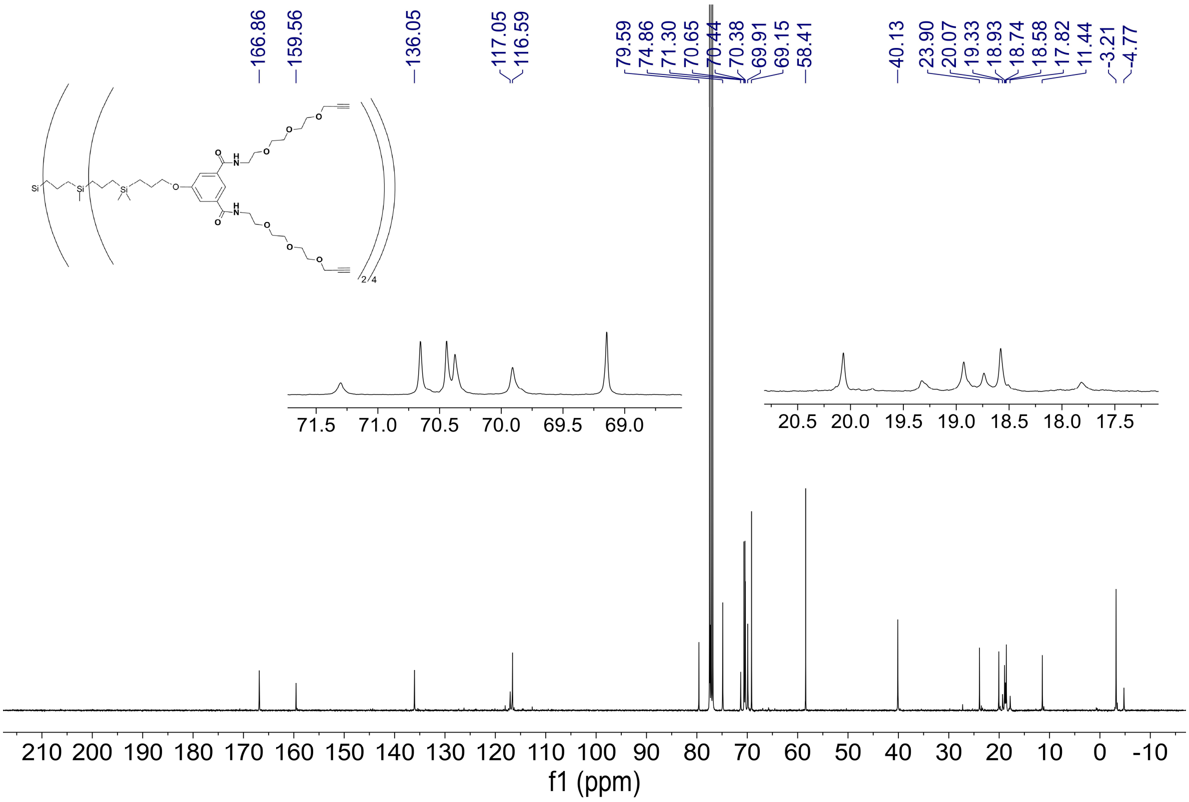

Figure S29: $^{13}C\{^{1}H\}$ NMR (400 MHz, $CDCl_3$) of **G_2-B (10)**.

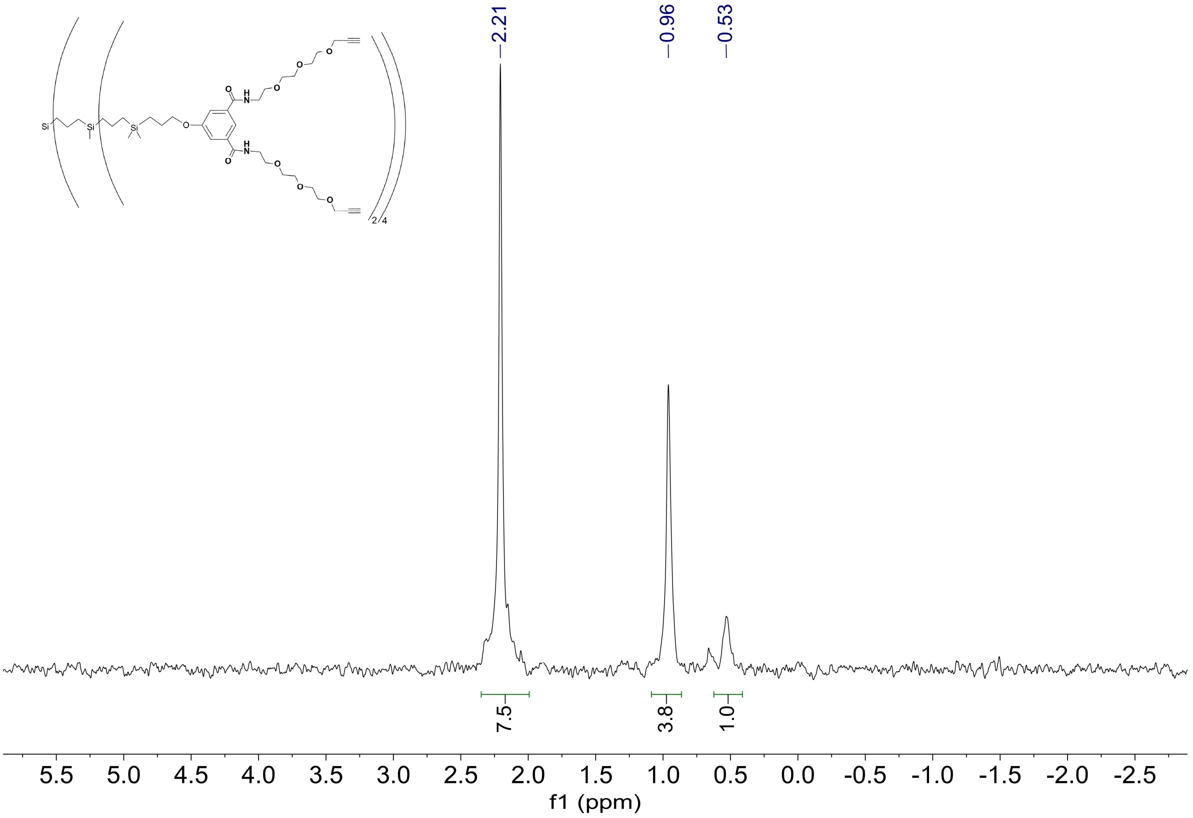

Figure S30: ^{29}Si INEPT NMR (400 MHz, $CDCl_3$) of **G_2-B (10)**.

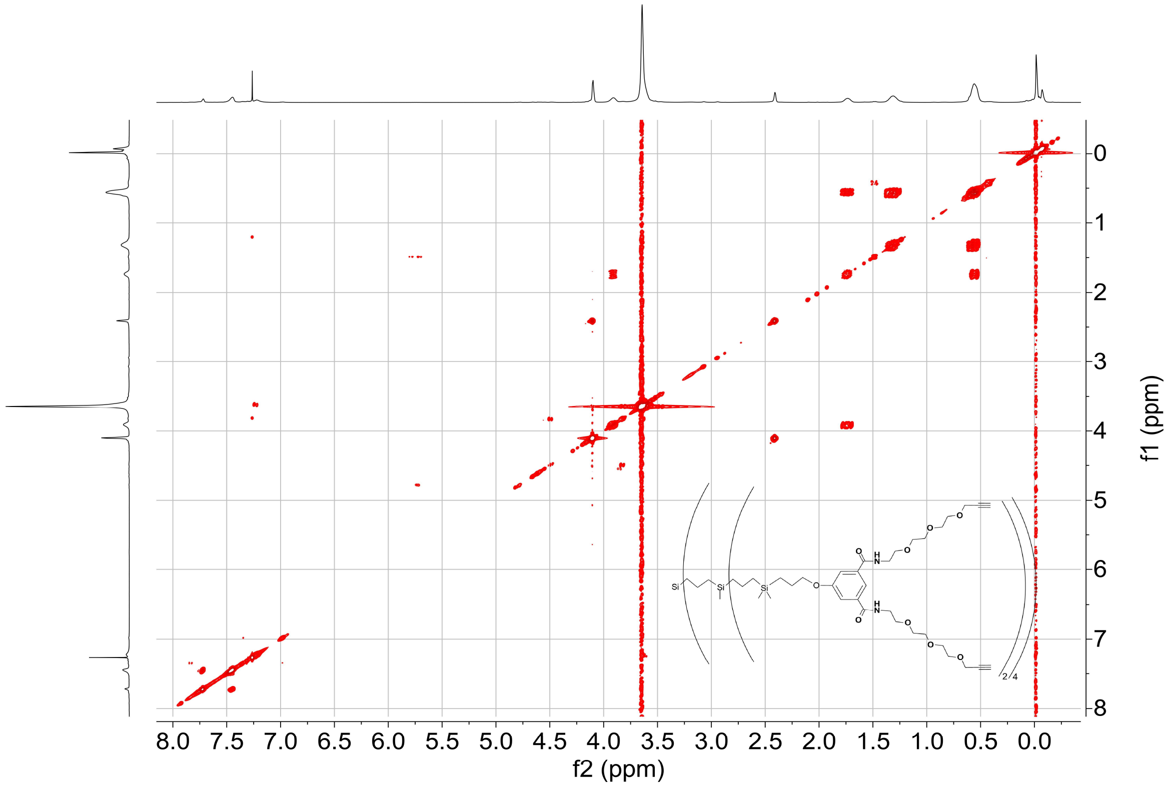

Figure S31: ^{1}H-^{1}H COSY NMR (400 MHz, $CDCl_3$) of **G_2-B (10)**.

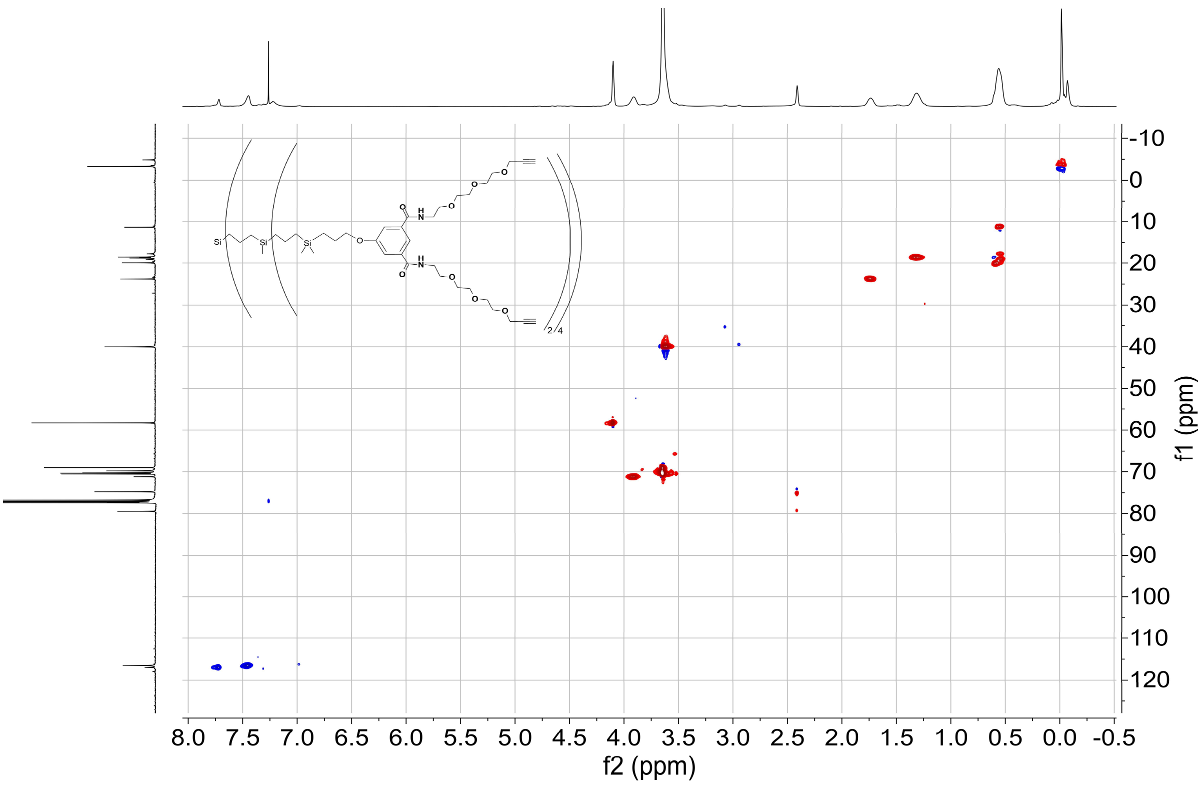

Figure S32: ^{1}H-^{13}C HSQC NMR (400 MHz, $CDCl_3$) of **G_2-B (10)**.

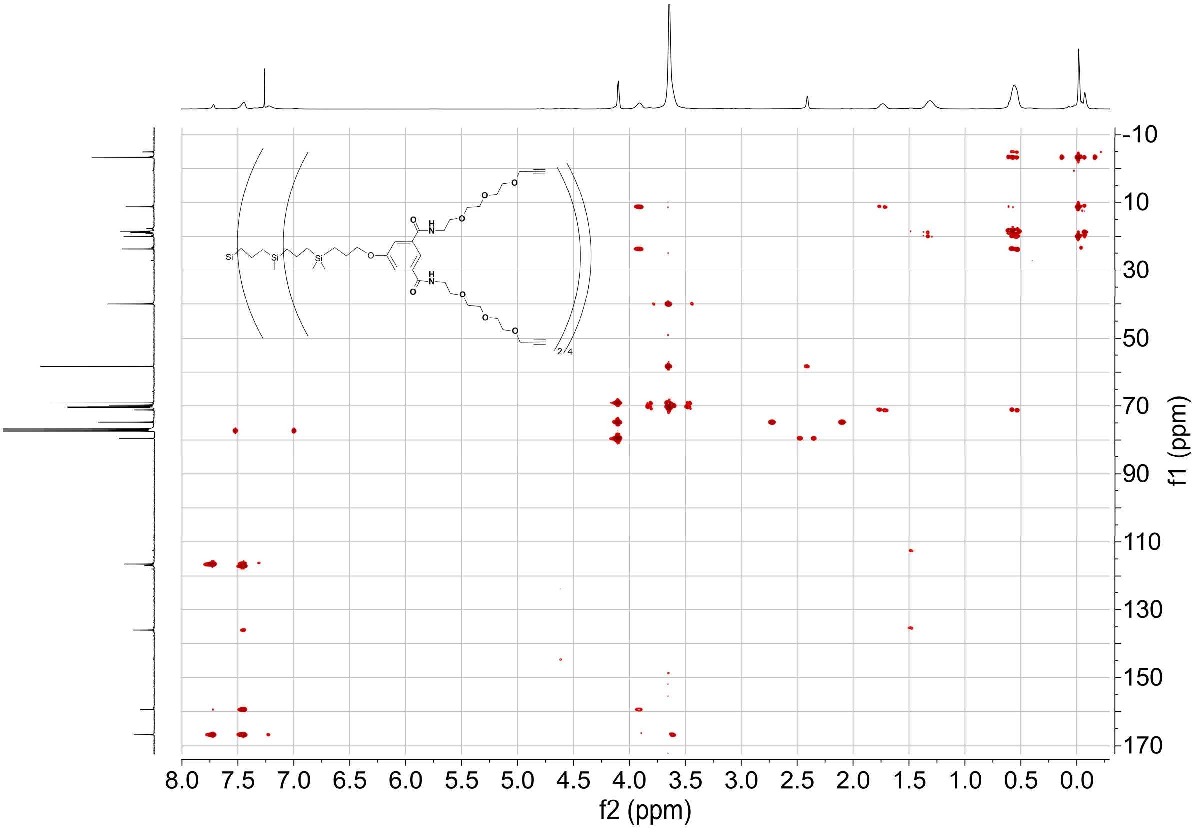

Figure S33: ^{1}H-^{13}C HMBC NMR (400 MHz, $CDCl_3$) of **G_2-B (10)**.

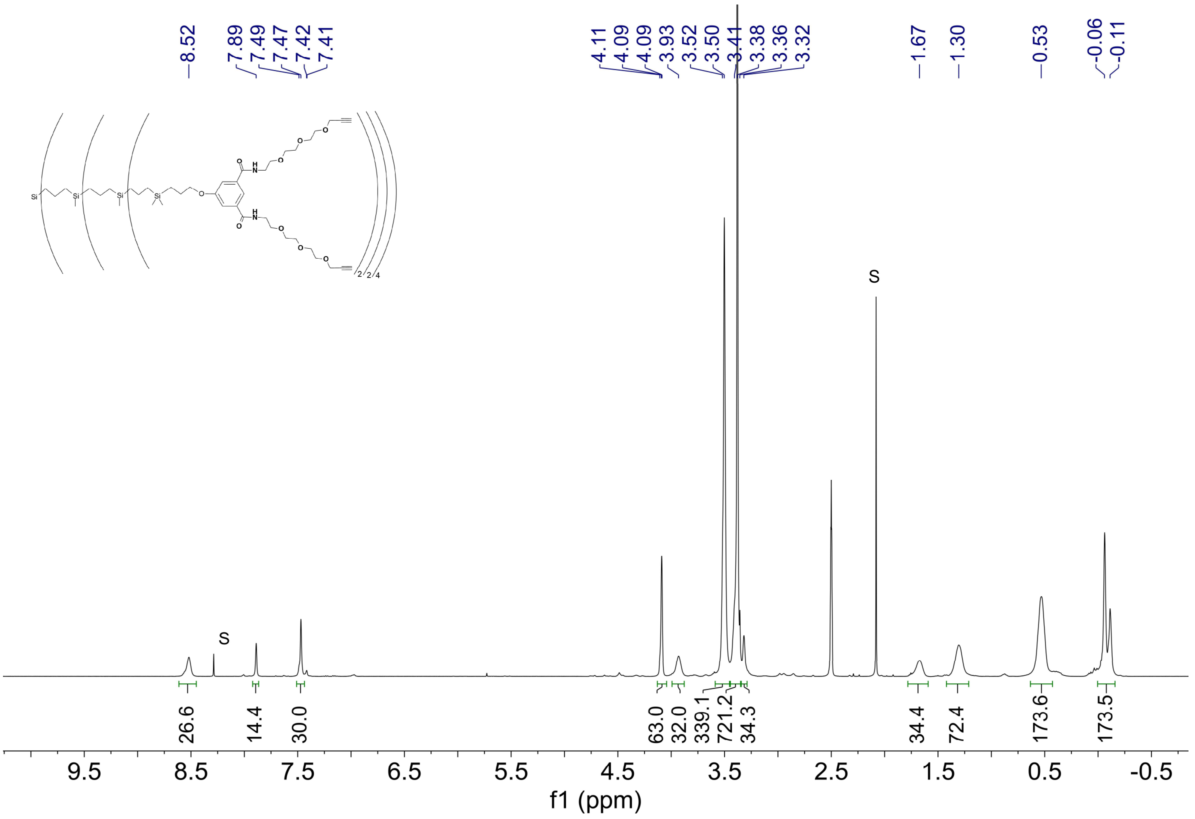

Figure S34: ^{1}H NMR (400 MHz, dmso-d_6) of **G$_3$-B (11)**.

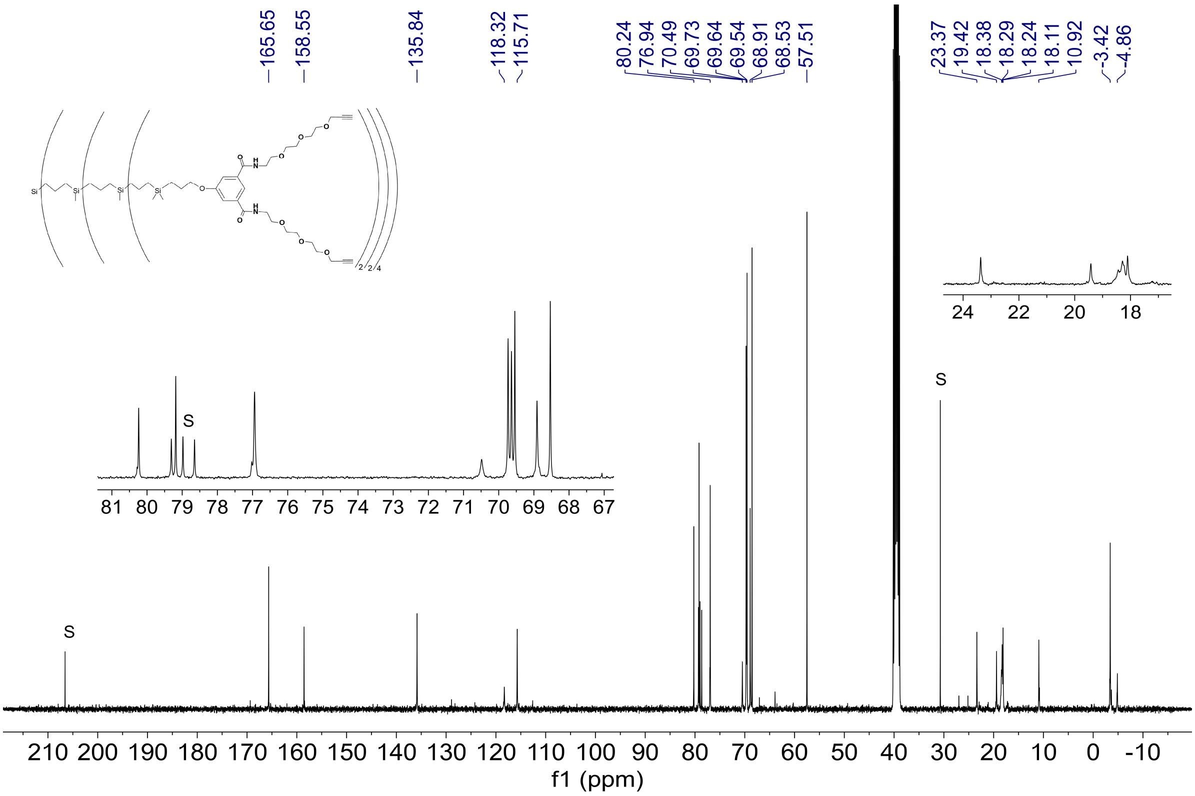

Figure S35: ^{13}C{^{1}H} NMR (400 MHz, dmso-d_6) of **G$_3$-B (11)**.

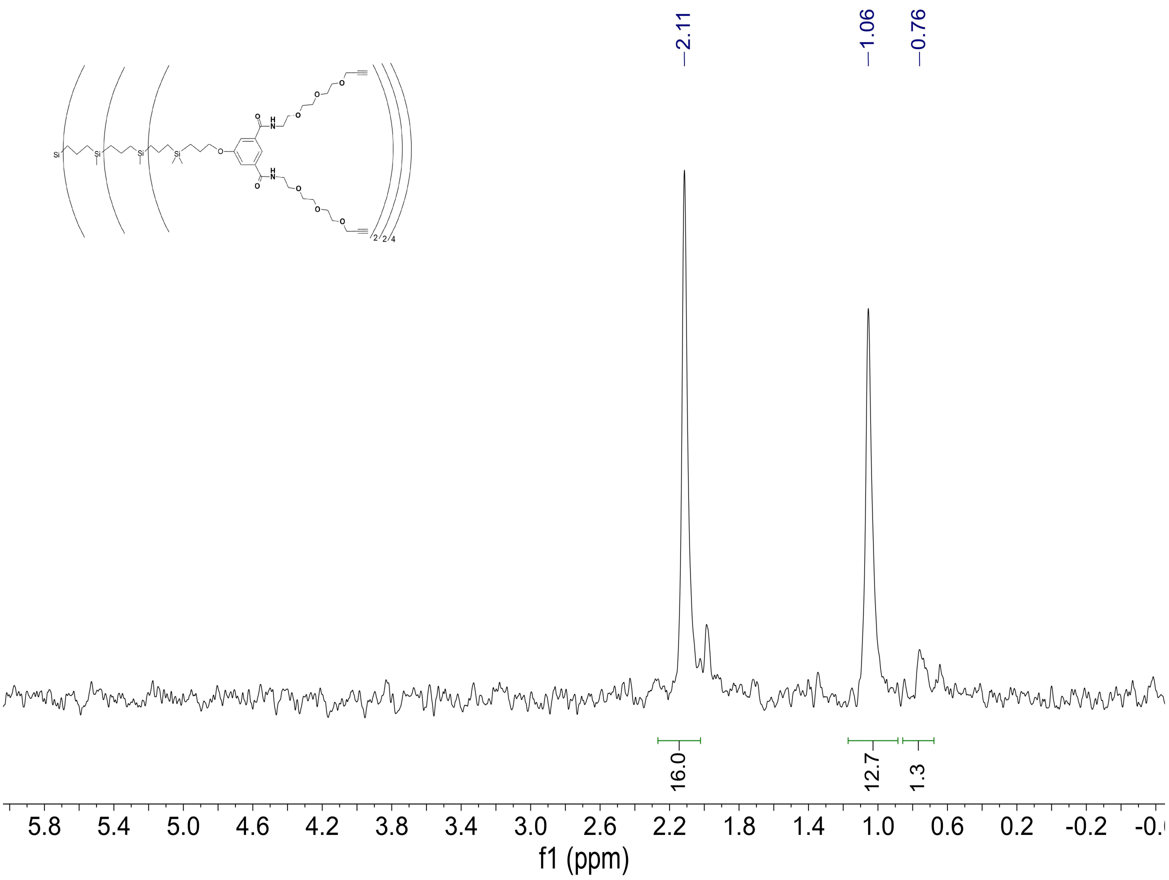

Figure S36: ^{29}Si INEPT NMR (400 MHz, dmso-d_6) of **G$_3$-B (11)**.

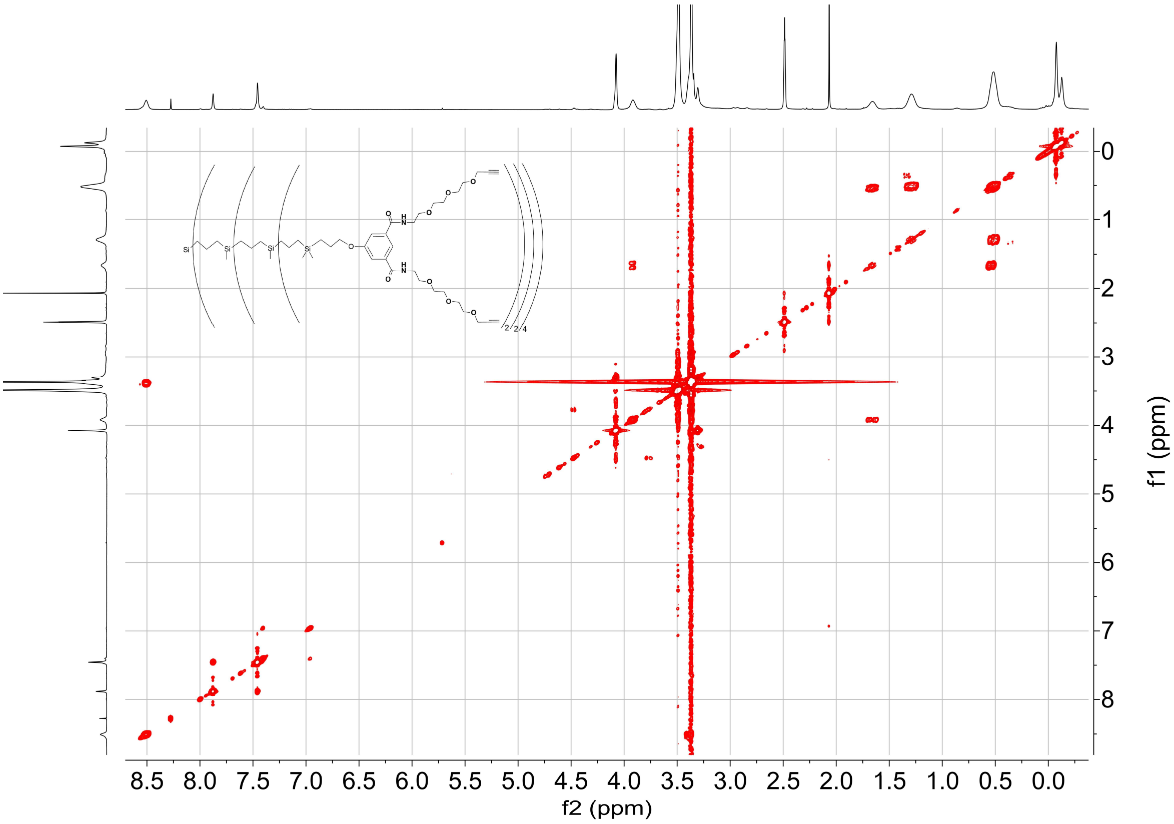

Figure S37: ^{1}H-^{1}H COSY NMR (400 MHz, dmso-d_6) of **G$_3$-B (11)**.

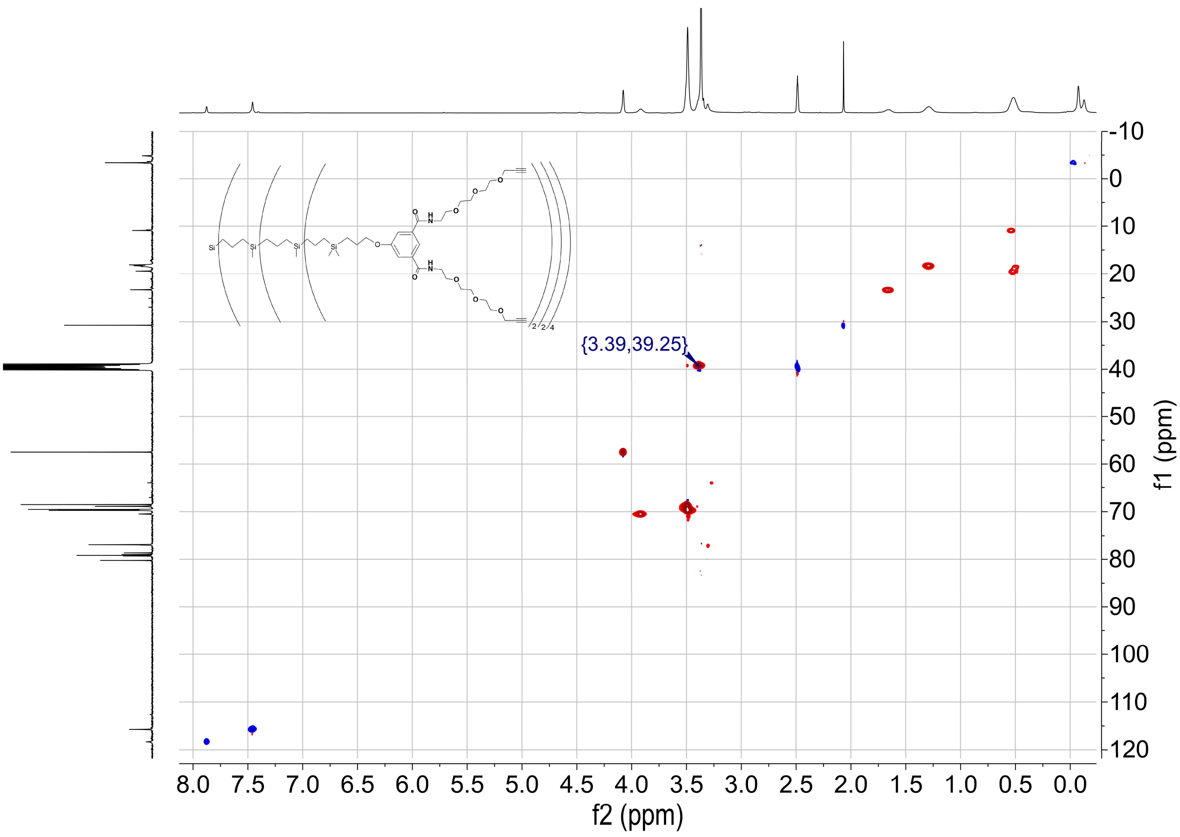

Figure S38: ^{1}H-^{13}C HSQC NMR (400 MHz, dmso-d_6) of **G$_3$-B (11)**.

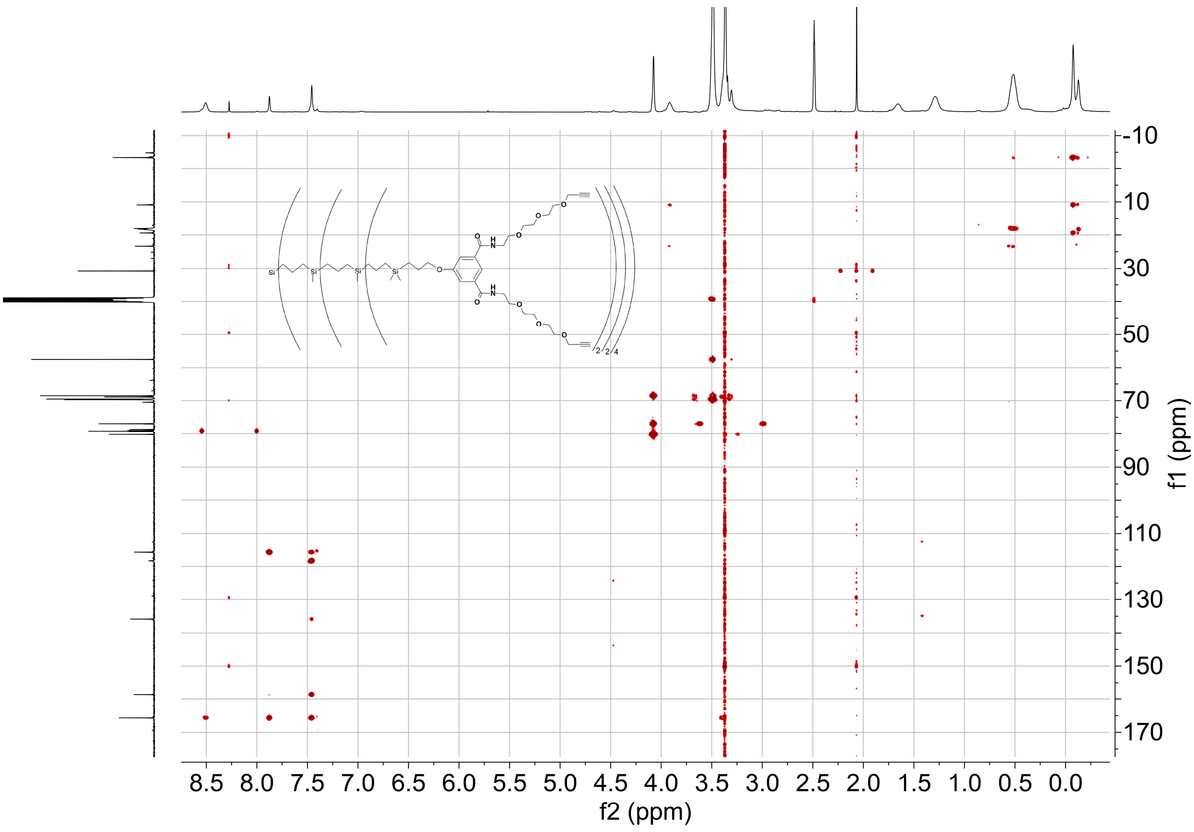

Figure S39: ^{1}H-^{13}C HMBC NMR (400 MHz, dmso-d_6) of **G$_3$-B (11)**.

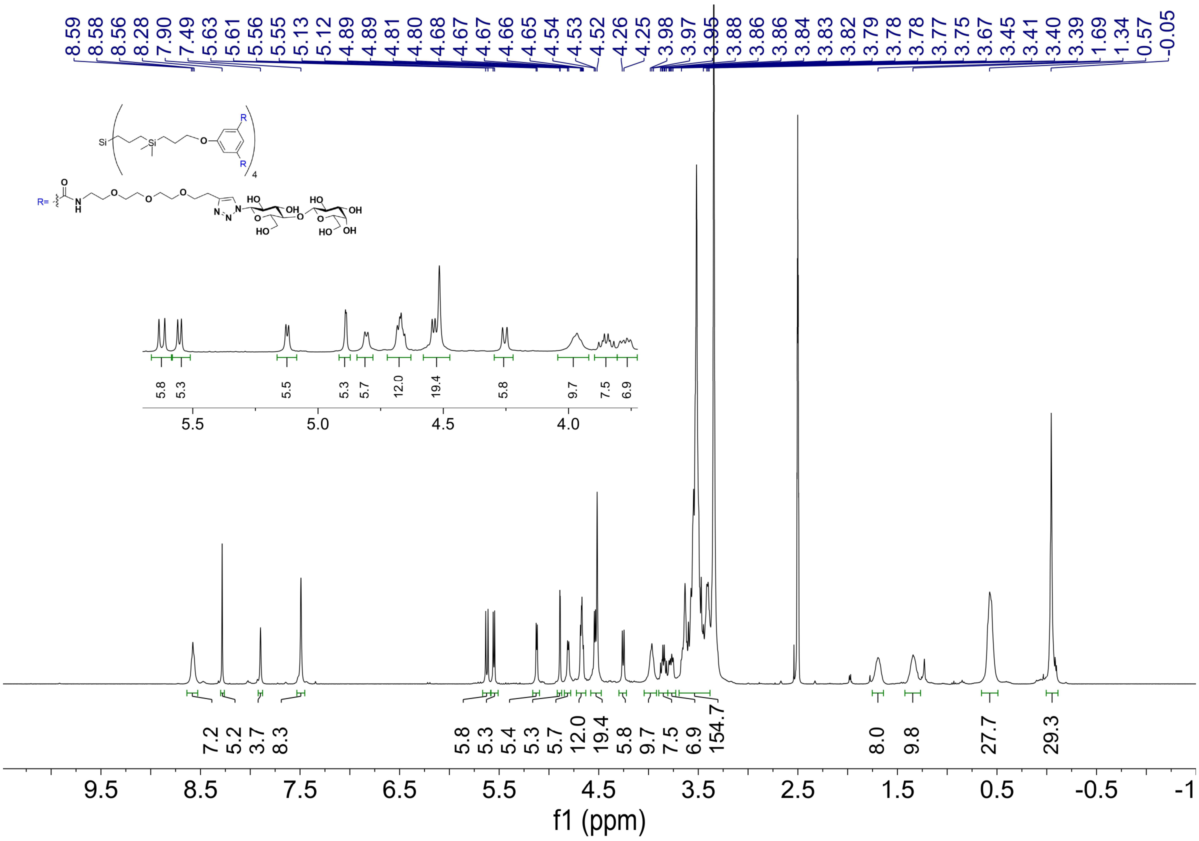

Figure S40: ^{1}H NMR (400 MHz, dmso-d_6) of **G$_1$-B-Lac$_8$ (15b)**.

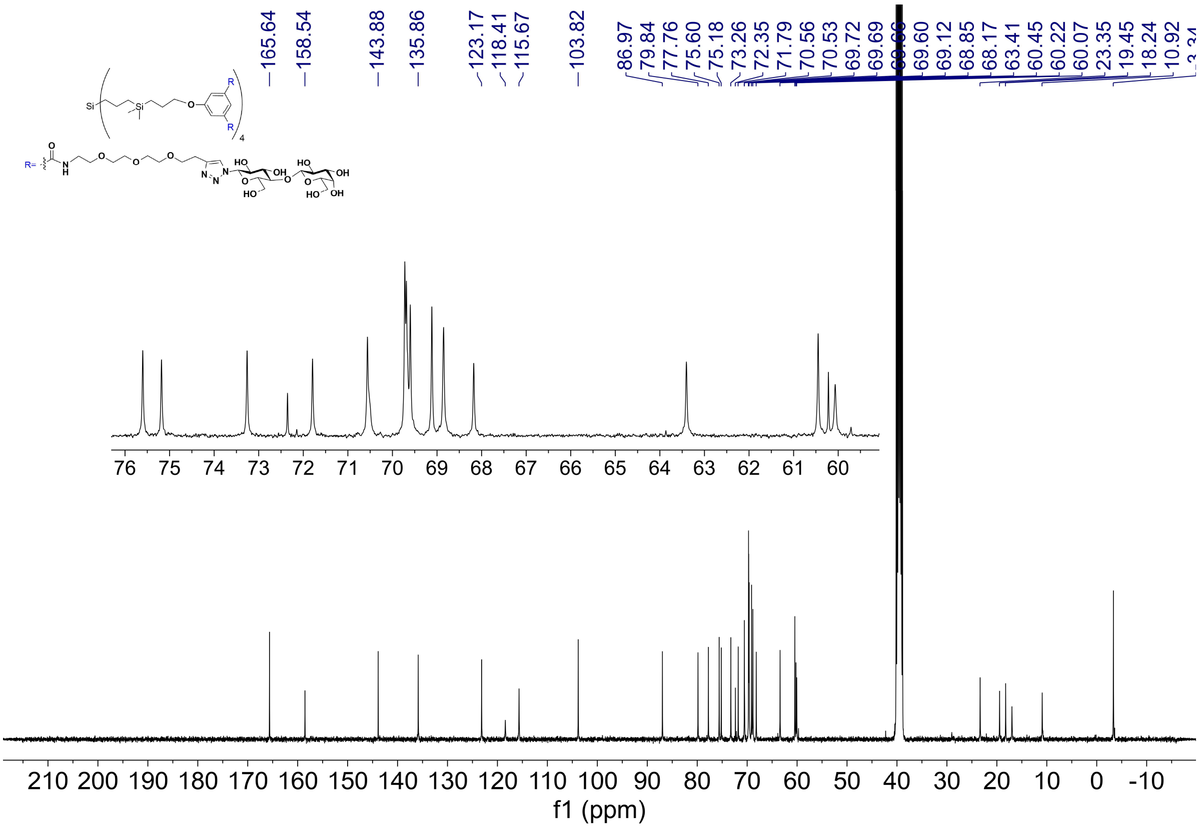

Figure S41: ^{13}C{^{1}H} NMR (400 MHz, dmso-d_6) of **G$_1$-B-Lac$_8$ (15b)**.

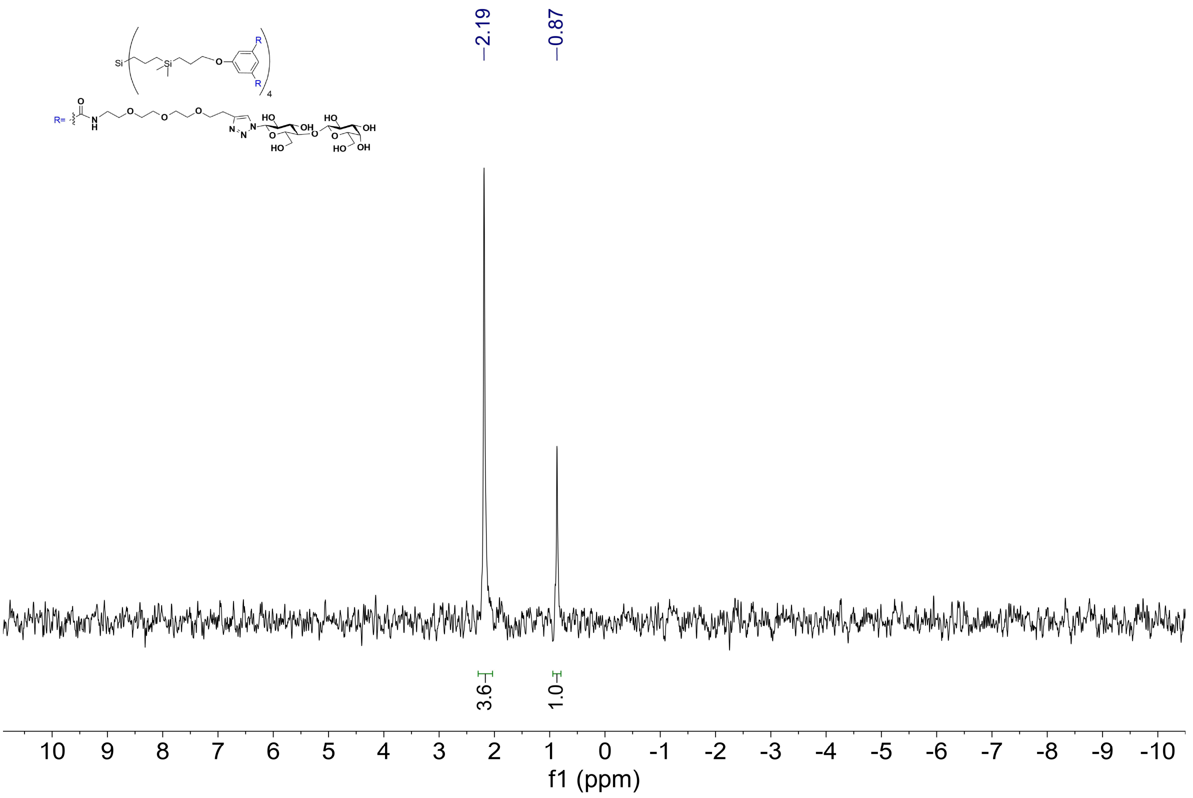

Figure S42: ^{29}Si INEPT NMR (400 MHz, dmso-d_6) of **G$_1$-B-Lac$_8$ (15b)**.

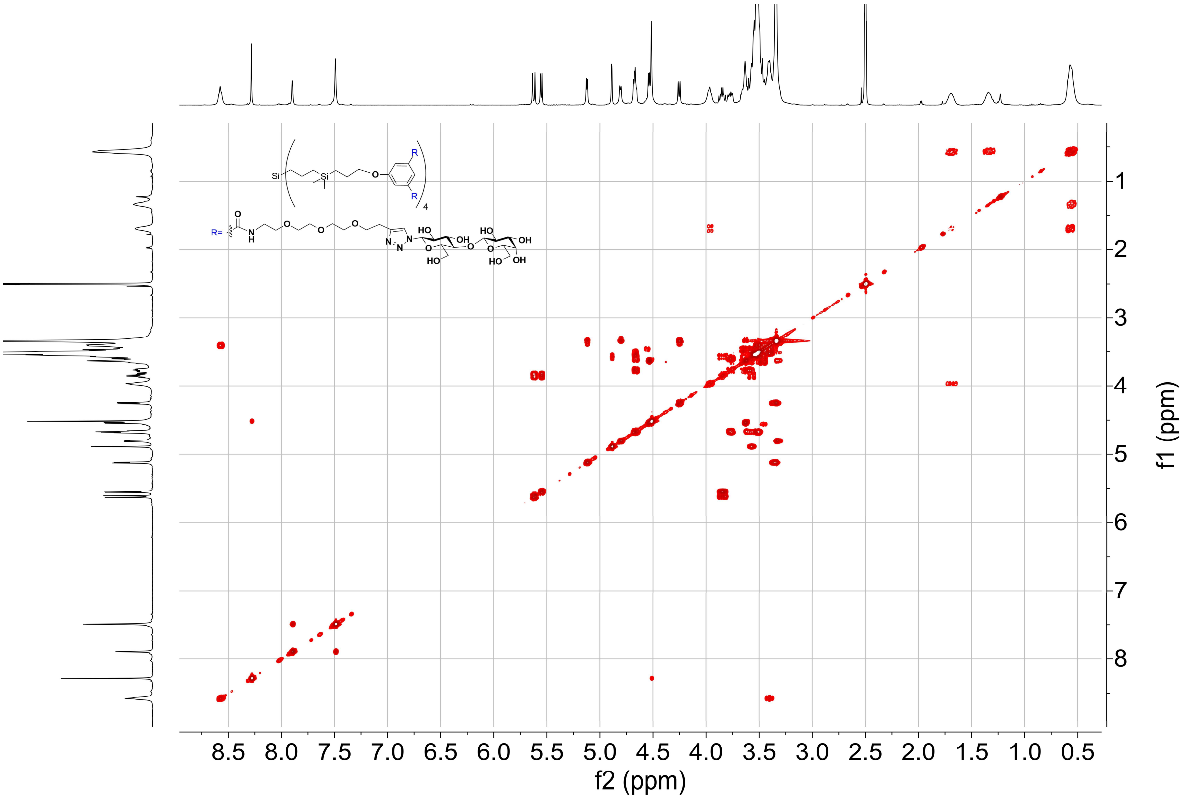

Figure S43: ^{1}H-^{1}H COSY NMR (400 MHz, dmso-d_6) of **G$_1$-B-Lac$_8$ (15b)**.

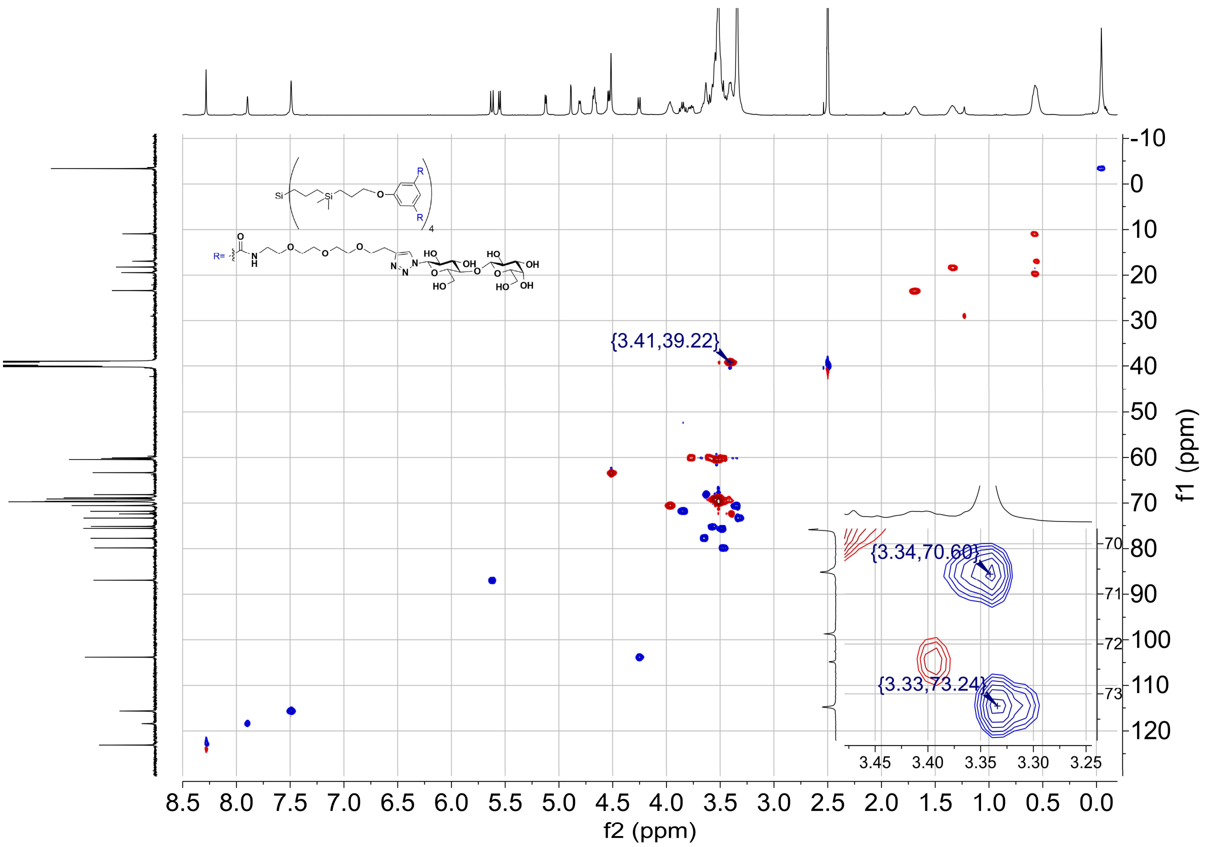

Figure S44: ^{1}H-^{13}C HSQC NMR (400 MHz, dmso-d_6) of **G$_1$-B-Lac$_8$ (15b)**.

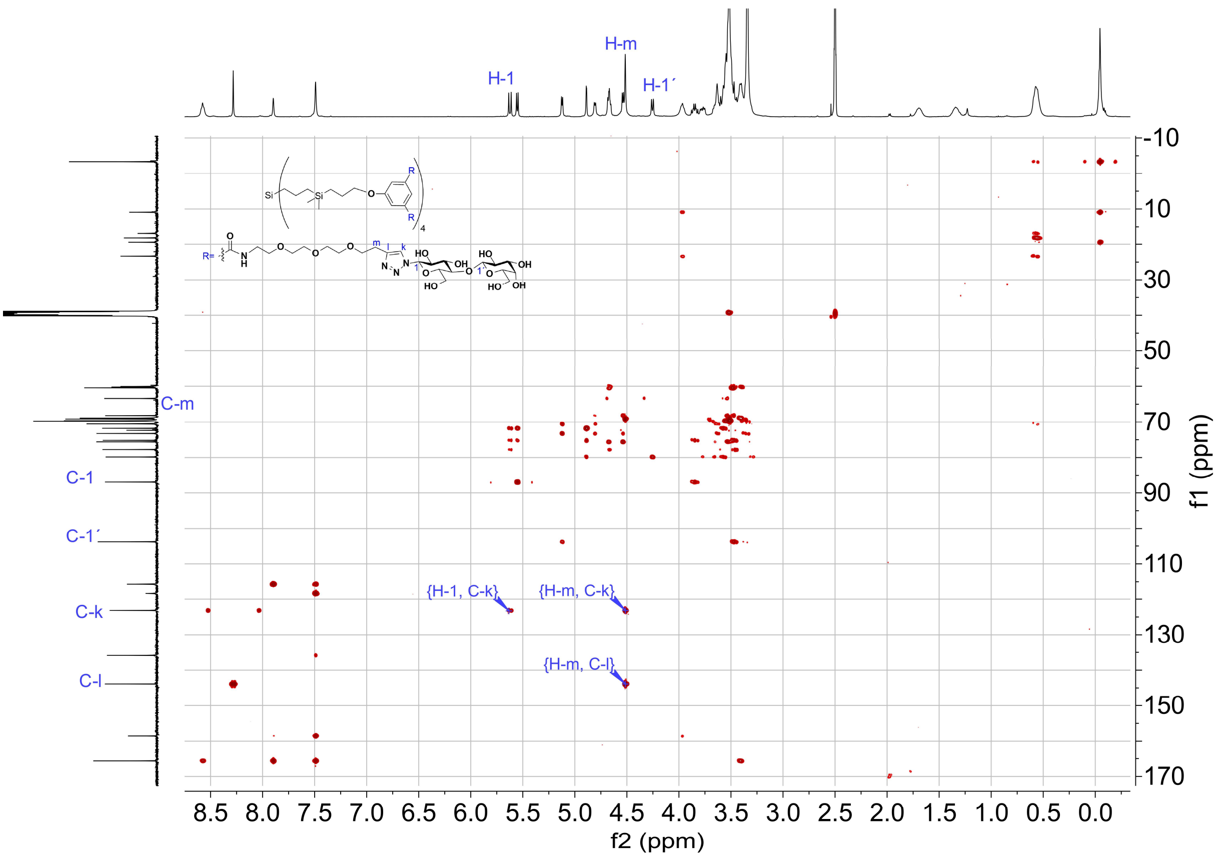

Figure S45: ^{1}H-^{13}C HMBC NMR (400 MHz, dmso-d_6) of **G$_1$-B-Lac$_8$ (15b)**.

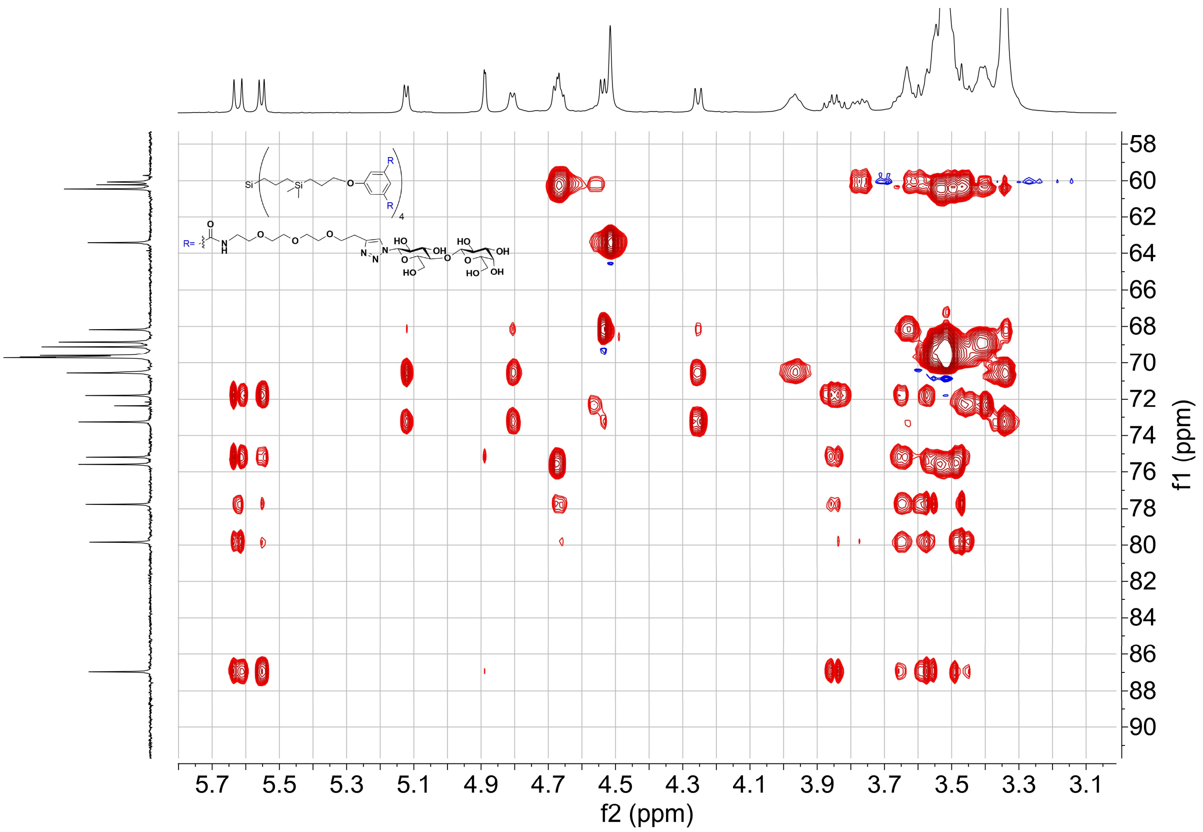

Figure S46: ^{1}H-^{13}C HSQC TOCSY NMR (400 MHz, dmso-d_6) of **G$_1$-B-Lac$_8$ (15b)**.

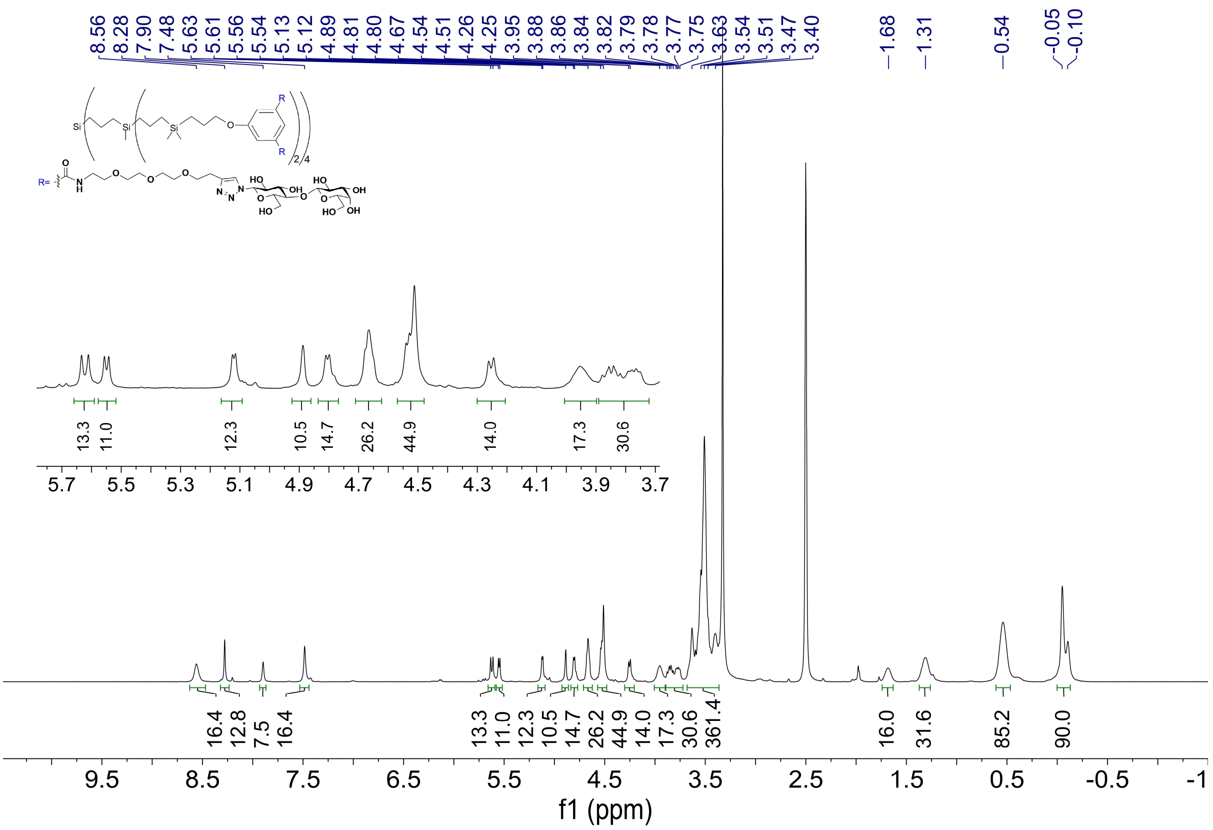

Figure S47: ^{1}H NMR (400 MHz, dmso-d_6) of **G$_2$-B-Lac$_{16}$ (16b)**.

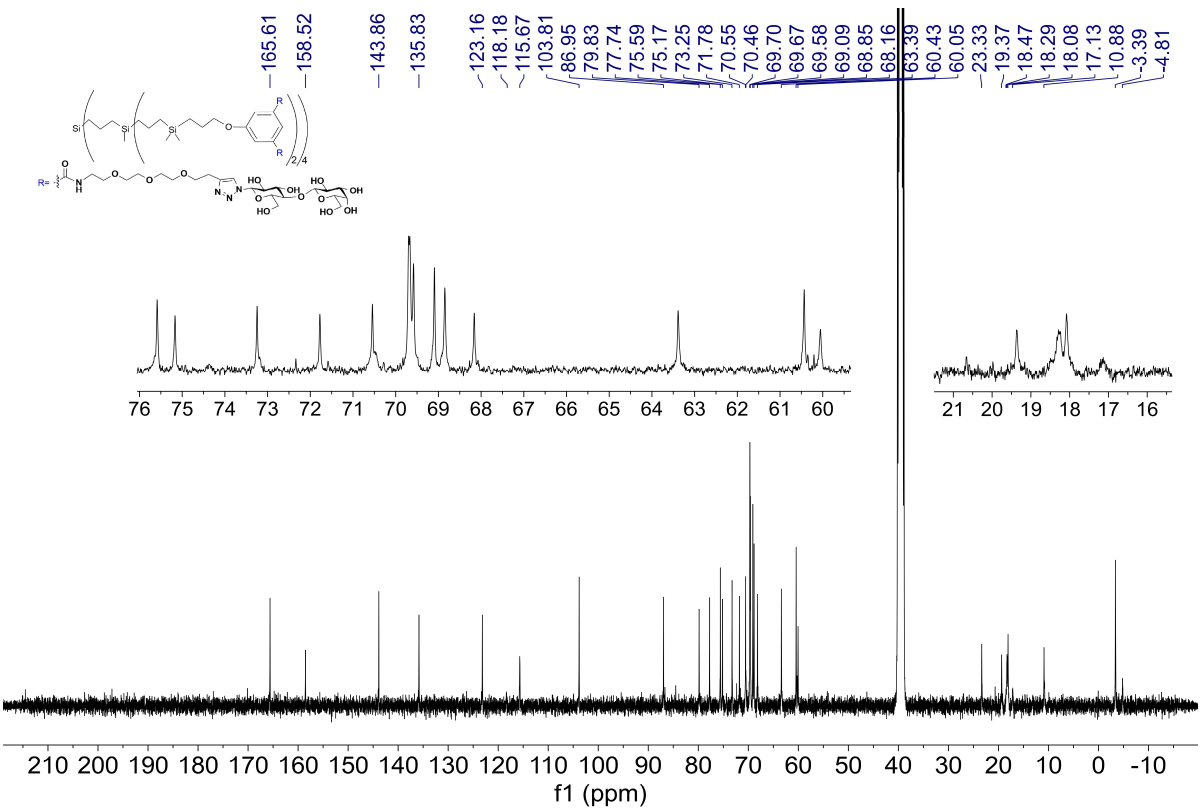

Figure S48: $^{13}C\{^1H\}$ NMR (400 MHz, dmso-d_6) of **G_2-B-Lac$_{16}$ (16b)**.

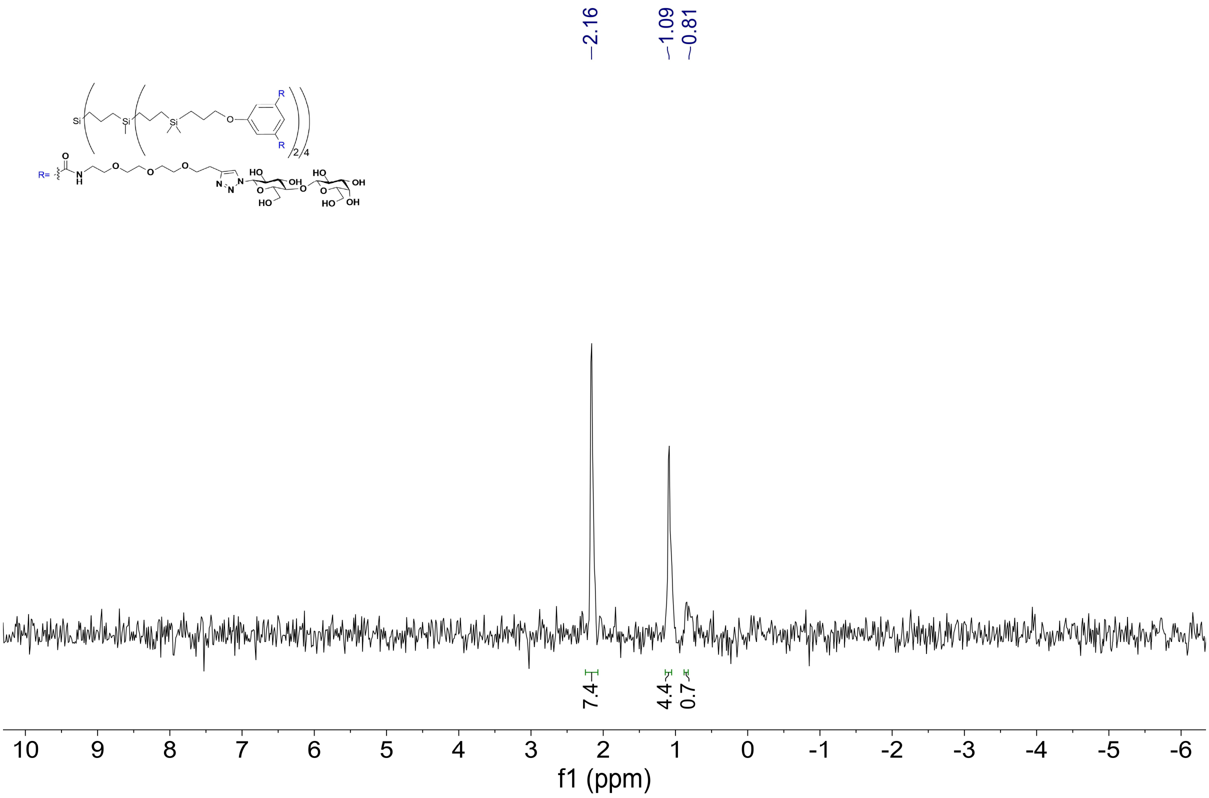

Figure S49: ^{29}Si INEPT NMR (400 MHz, dmso-d_6) of **G_2-B-Lac$_{16}$ (16b)**.

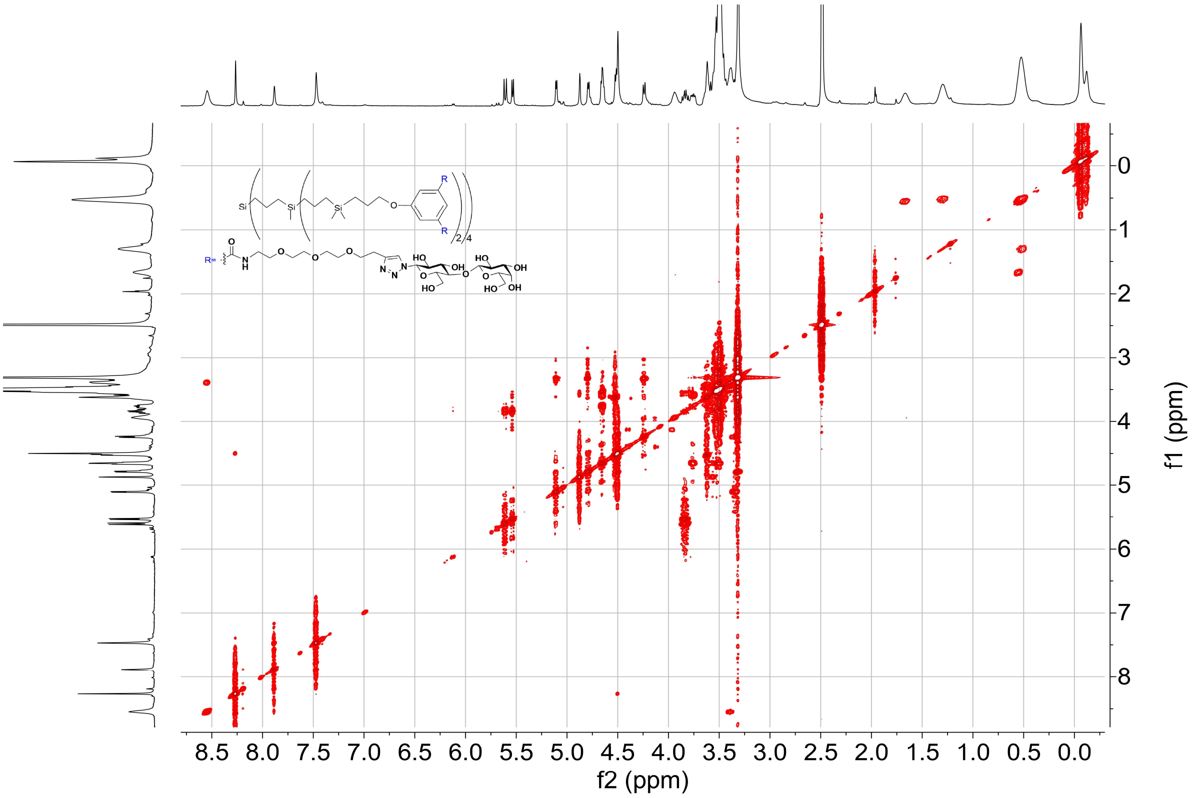

Figure S50: ^{1}H-^{1}H COSY NMR (400 MHz, dmso-d_6) of **G_2-B-Lac$_{16}$ (16b)**.

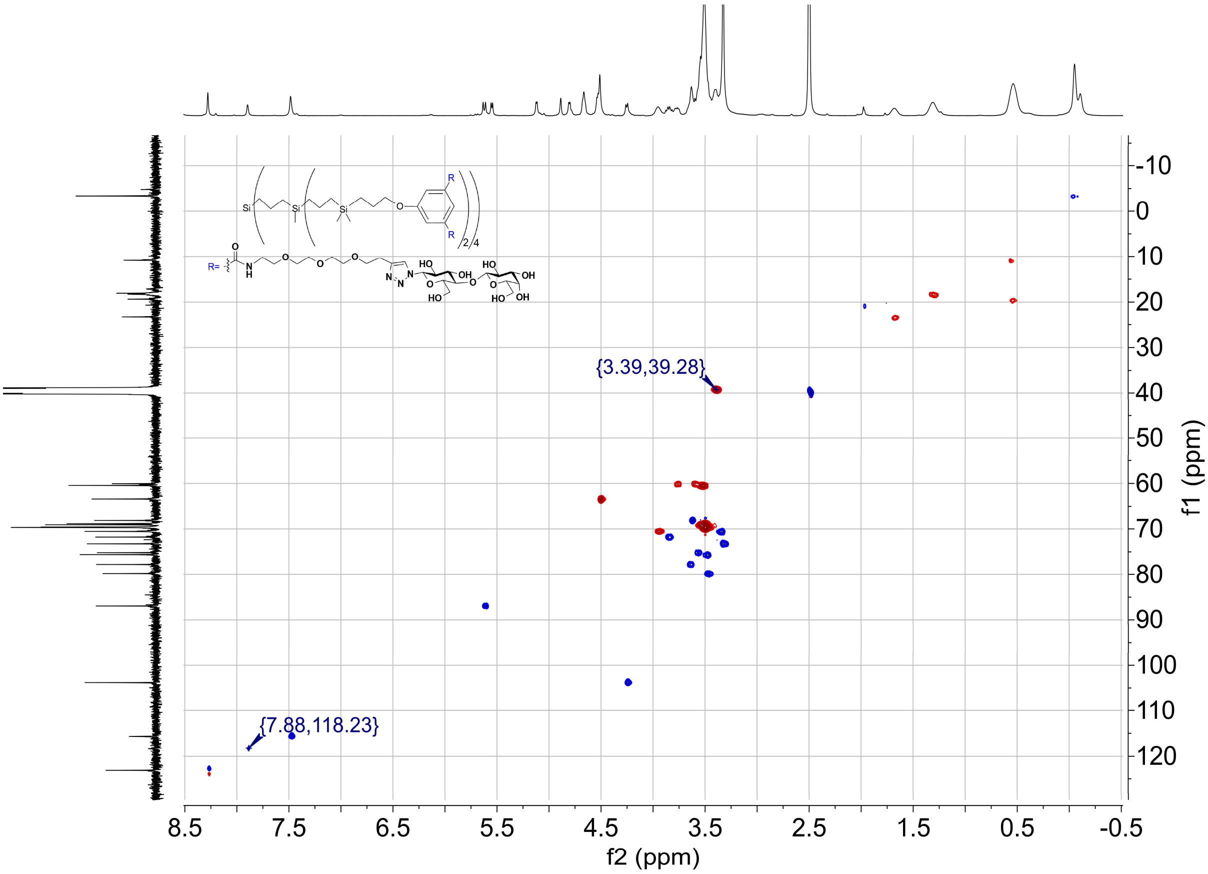

Figure S51: ^{1}H-^{13}C HSQC NMR (400 MHz, dmso-d_6) of **G_2-B-Lac$_{16}$ (16b)**.

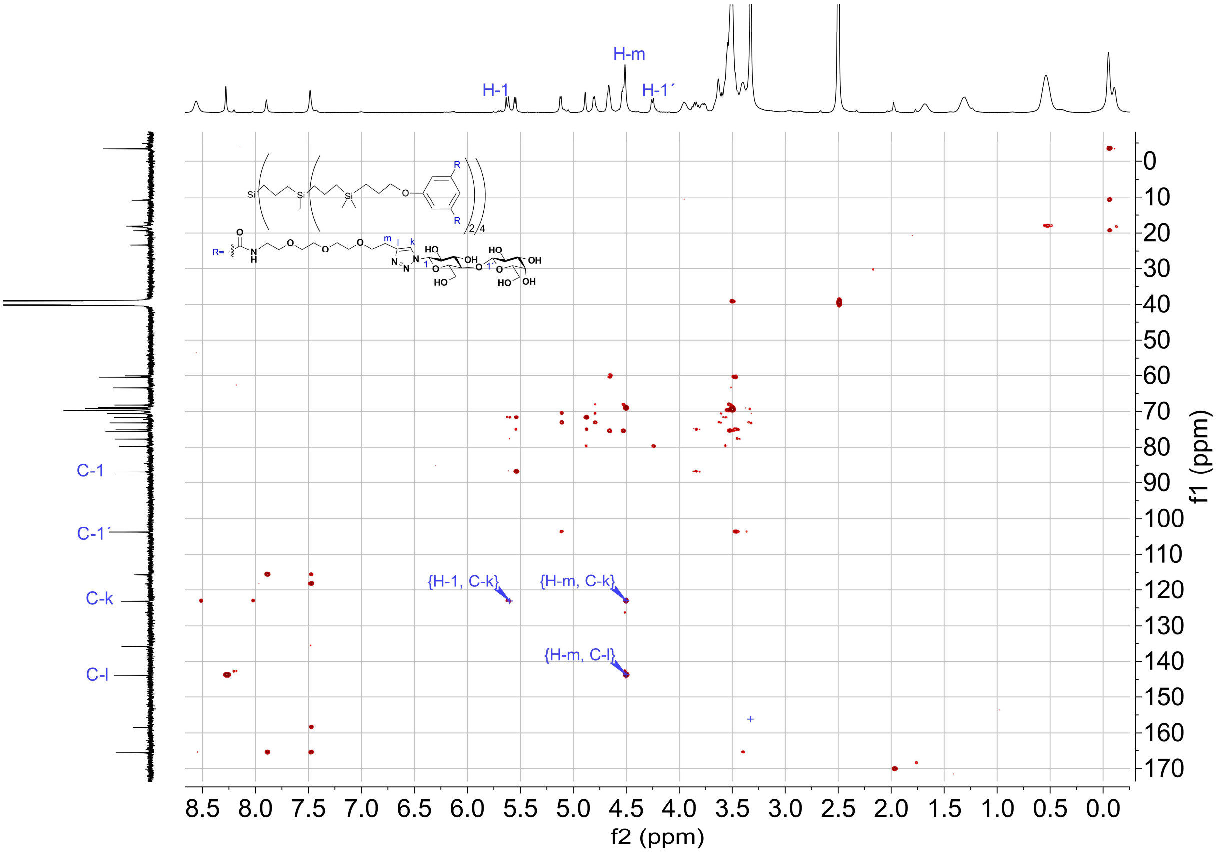

Figure S52: ^{1}H-^{13}C HMBC NMR (400 MHz, dmso-d_6) of **G_2-B-Lac$_{16}$ (16b)**.

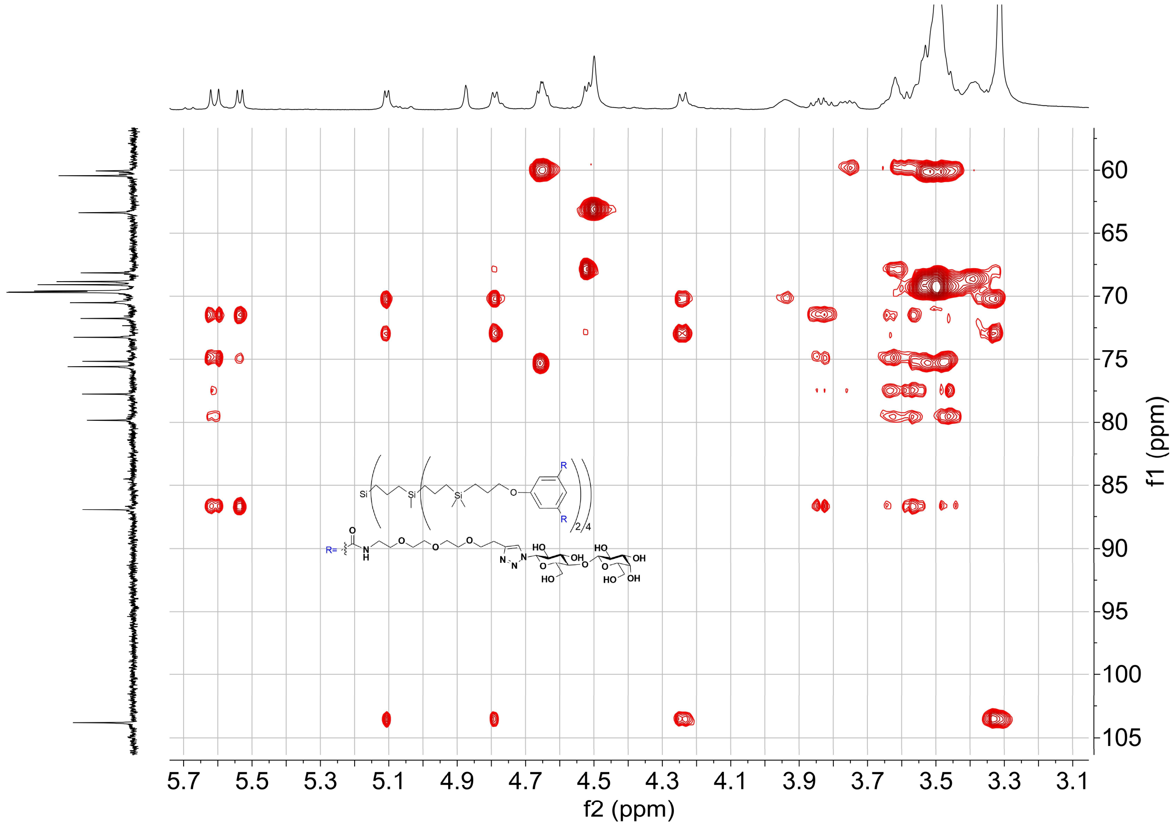

Figure S53: ^{1}H-^{13}C HSQC TOCSY NMR (400 MHz, dmso-d_6) of **G_2-B-Lac$_{16}$ (16b)**.

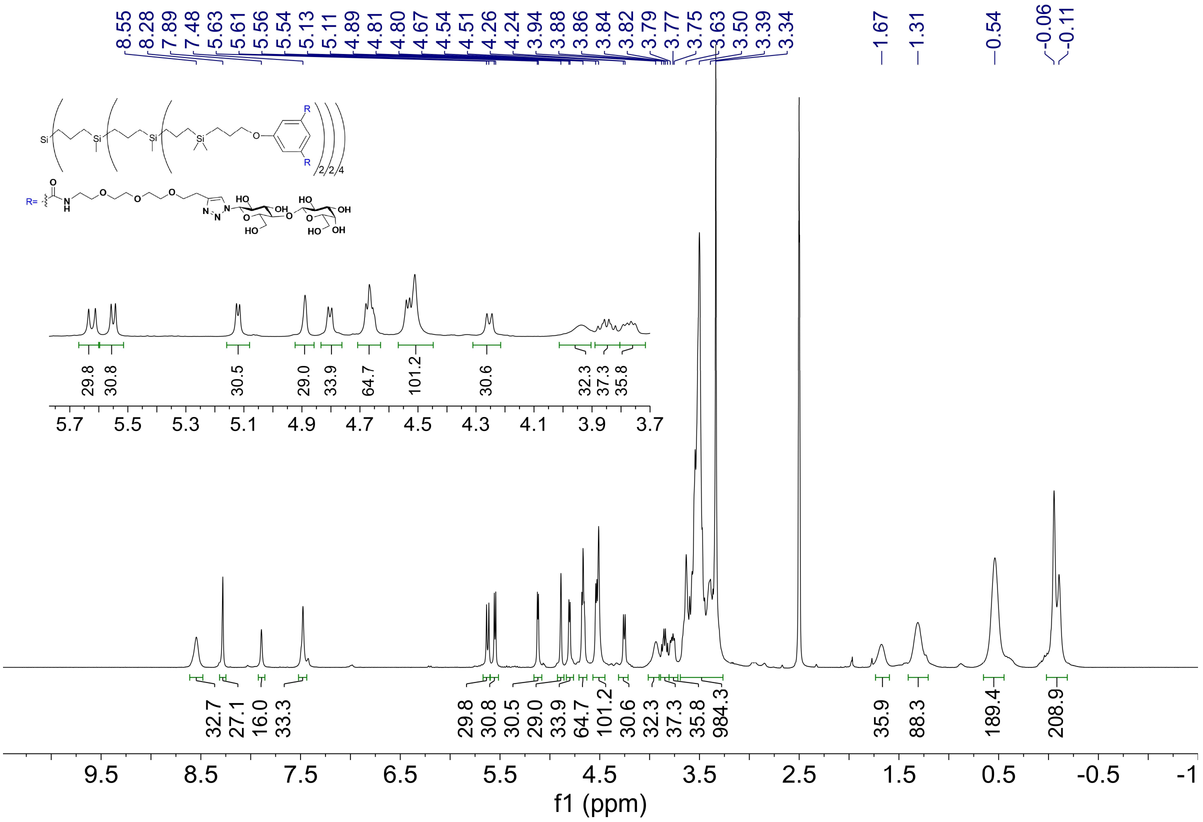

Figure S54: ^{1}H NMR (400 MHz, dmso-d_6) of **G_3-B-Lac$_{32}$ (17b)**.

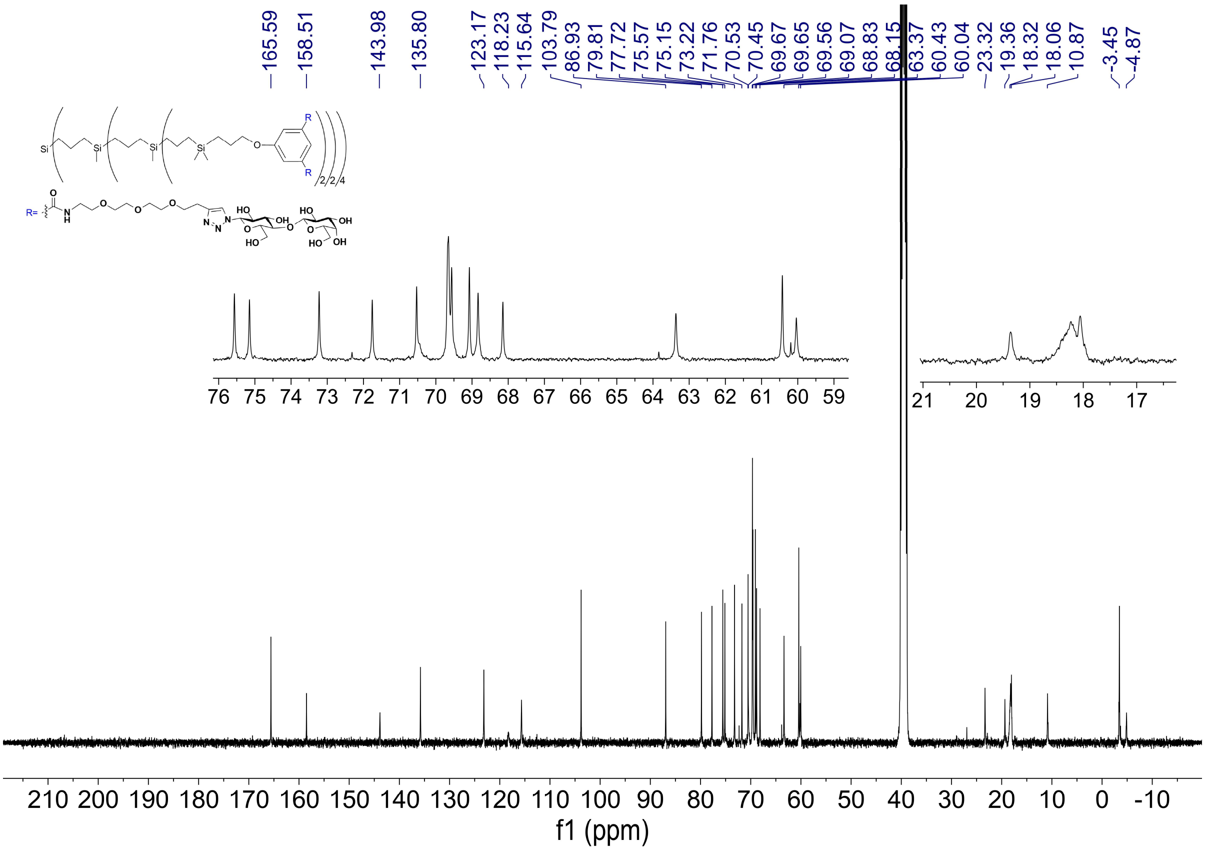

Figure S55: ^{13}C{^{1}H} NMR (400 MHz, dmso-d_6) of **G_3-B-Lac$_{32}$ (17b)**.

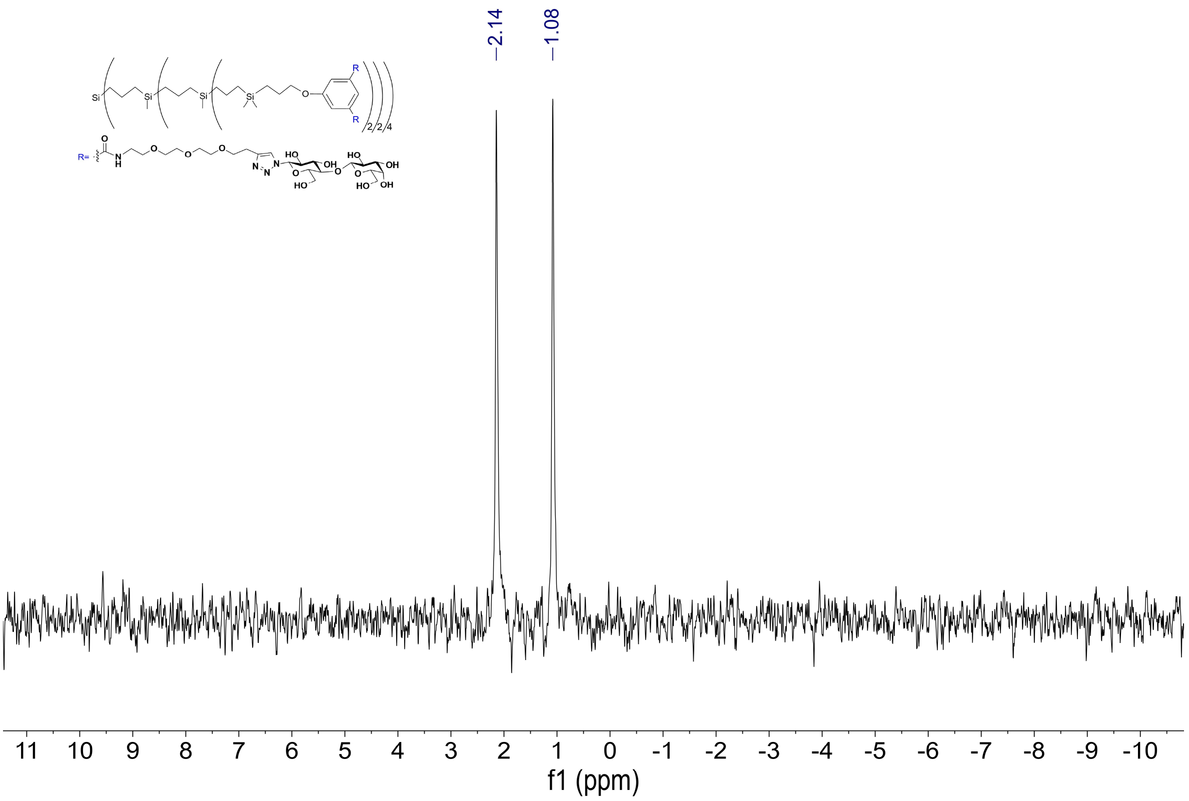

Figure S56: ^{29}Si INEPT NMR (400 MHz, dmso-d_6) of **G_3-B-Lac$_{32}$ (17b)**.

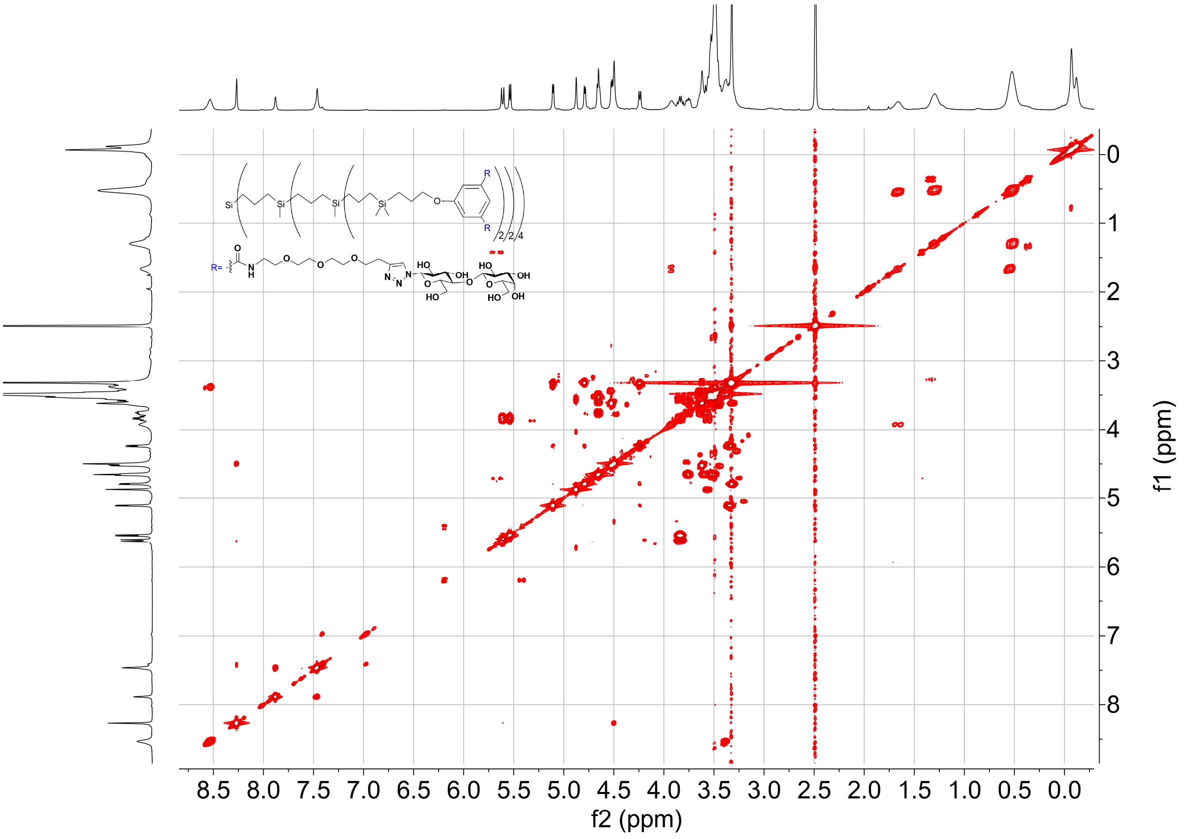

Figure S57: ^{1}H-^{1}H COSY NMR (400 MHz, dmso-d_6) of **G_3-B-Lac$_{32}$ (17b)**.

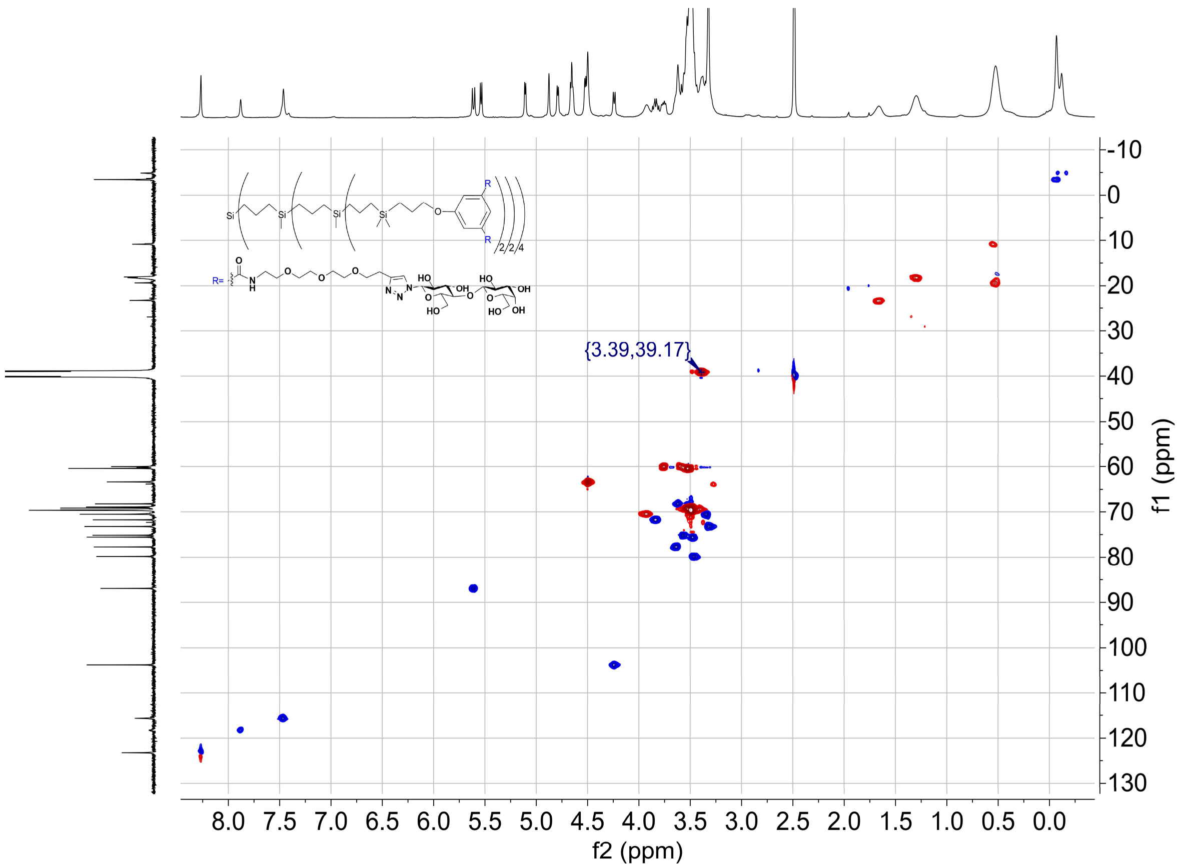

Figure S58: ^{1}H-^{13}C HSQC NMR (400 MHz, dmso-d_6) of **G$_3$-B-Lac$_{32}$ (17b)**.

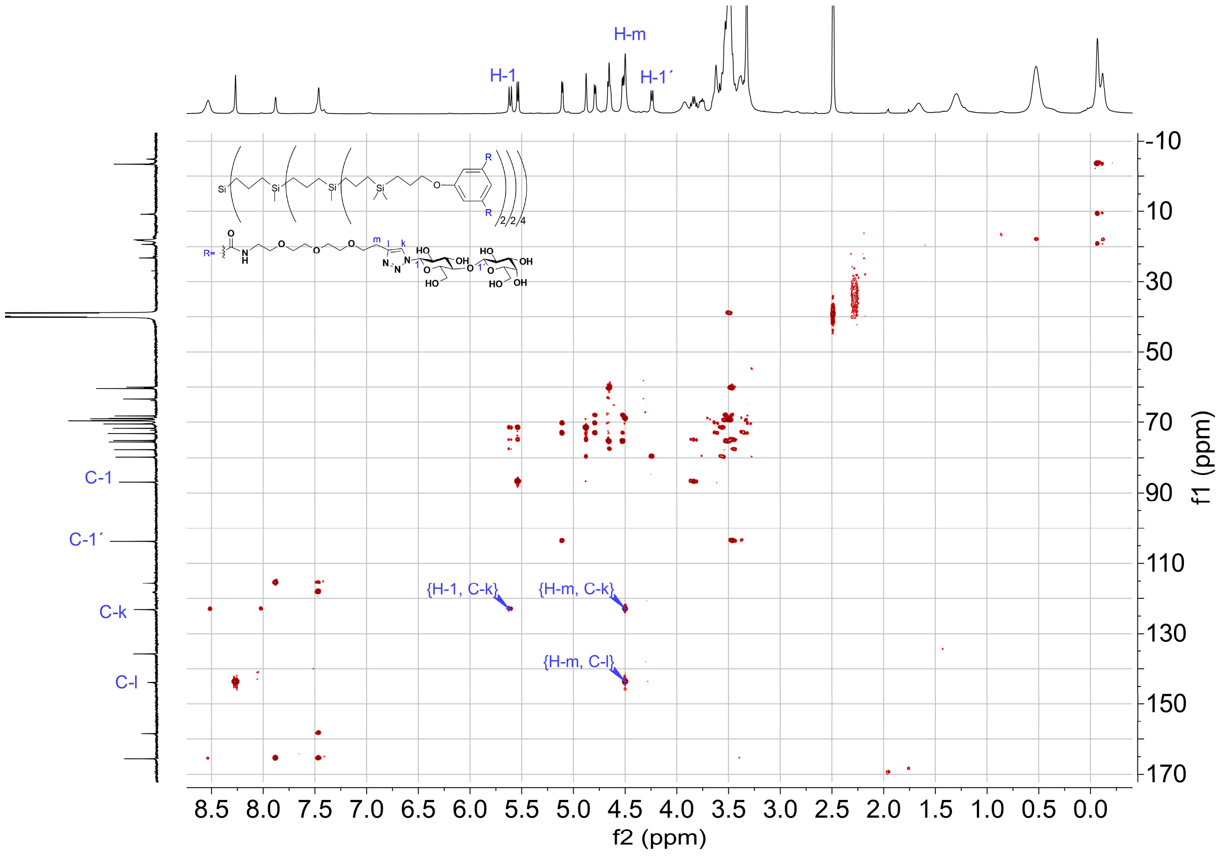

Figure S59: ^{1}H-^{13}C HMBC NMR (400 MHz, dmso-d_6) of **G$_3$-B-Lac$_{32}$ (17b)**.

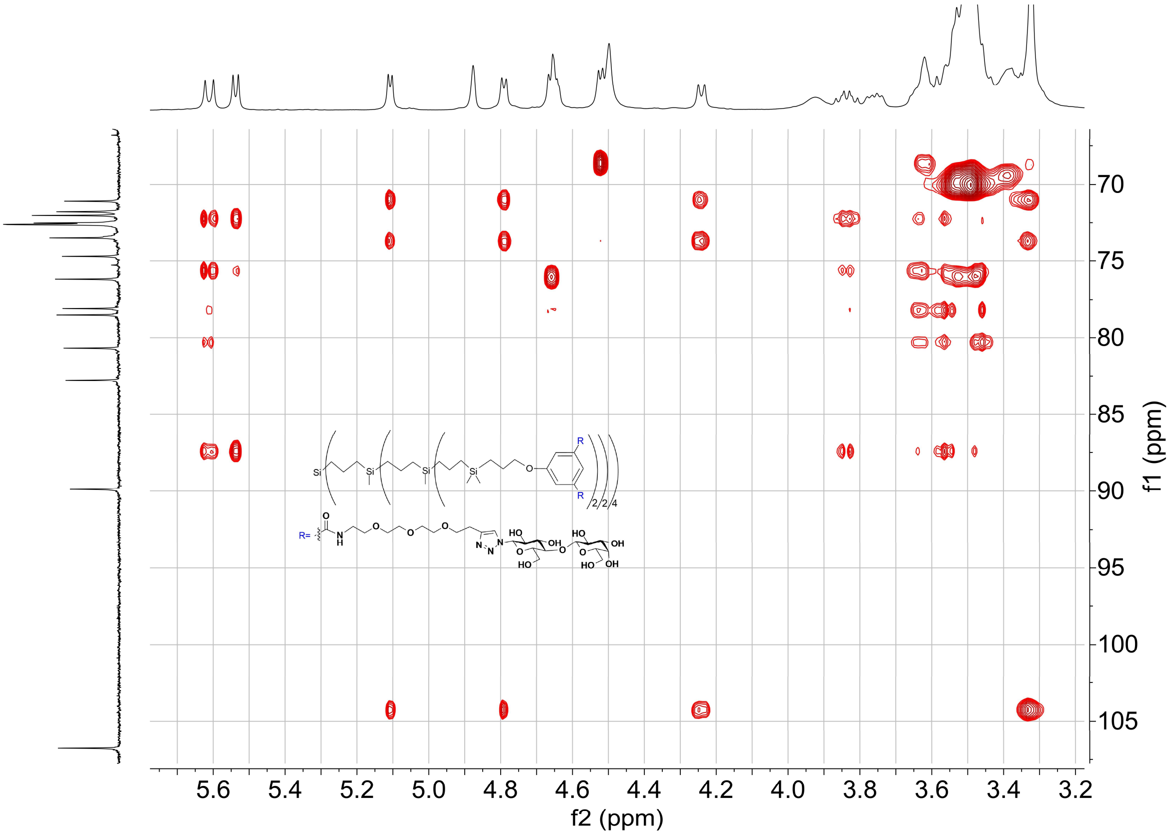

6. MALDI-TOF, IR, GPC

Figure S60: ^{1}H-^{13}C HSQC TOCSY NMR (400 MHz, dmso-d_6) of **G$_3$-B-Lac$_{32}$ (17b)**.

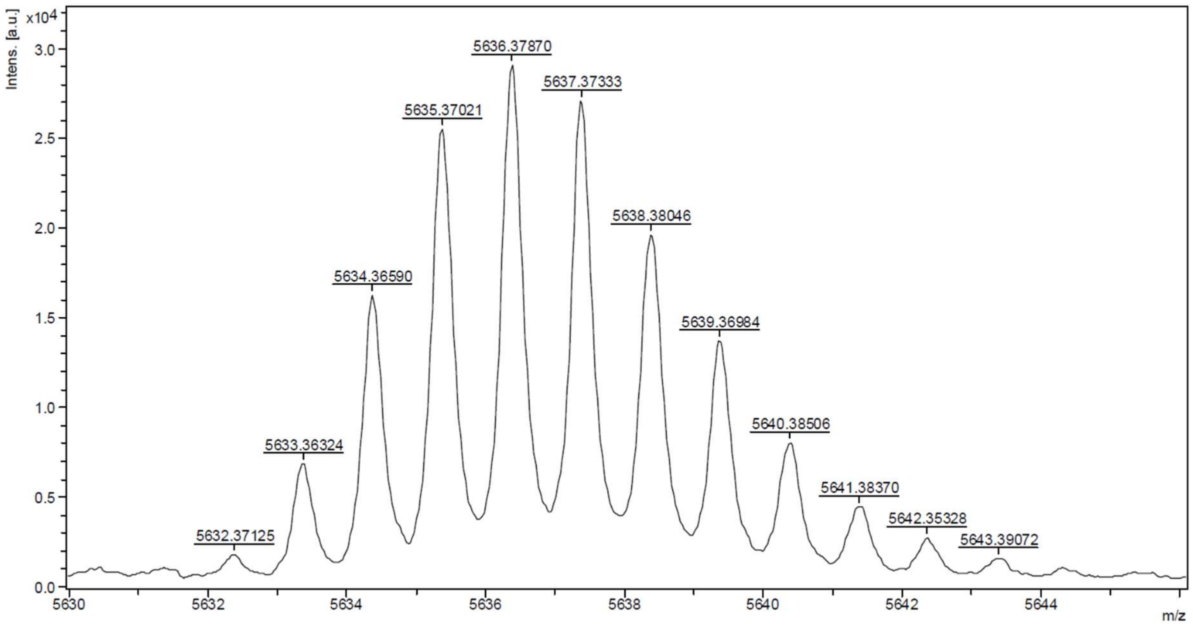

Figure S61: MALDI-TOF spectrum of glycodendrimer **G$_1$-A-Lac$_8$ (12b)**.

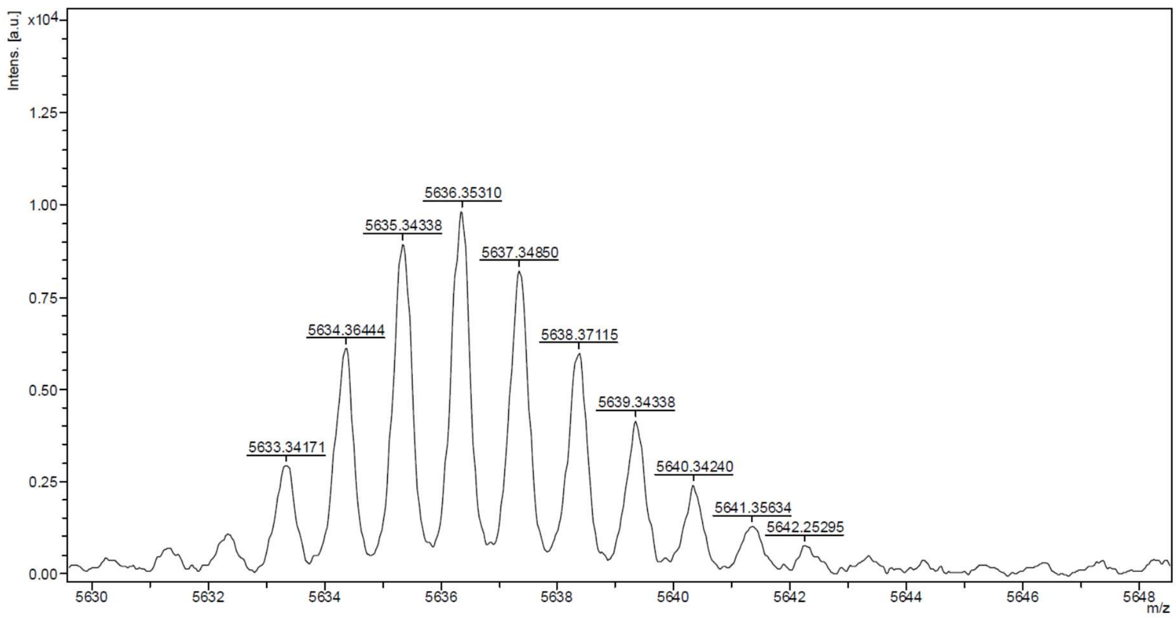

Figure S62: MALDI-TOF spectrum of glycodendrimer **G_1-B-Lac$_8$ (15b)**.

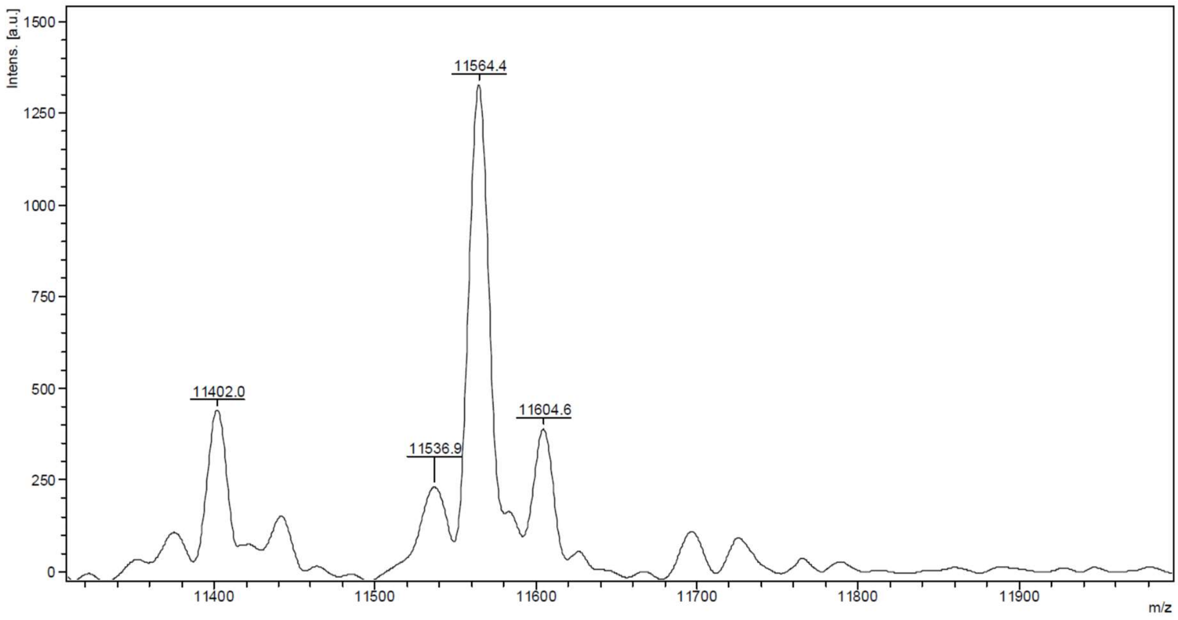

Figure S63: MALDI-TOF spectrum of glycodendrimer **G_2-A-Lac$_{16}$ (13b)**.

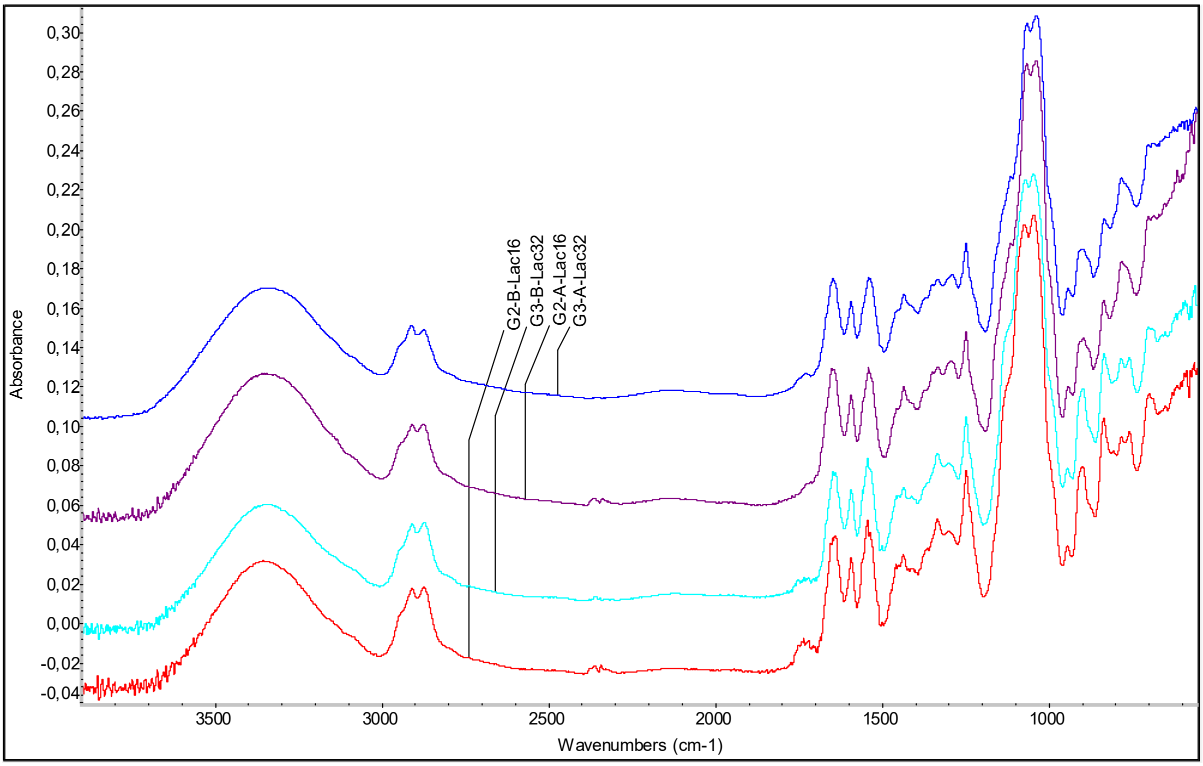

Figure S64: IR spectra of glycodendrimers: **G2-A-Lac16 (13b)** – blue, **G3-A-Lac32 (14b)** – violet, **G2-B-Lac16 (16b)** – red, **G3-B-Lac32 (17b)** – cyan.

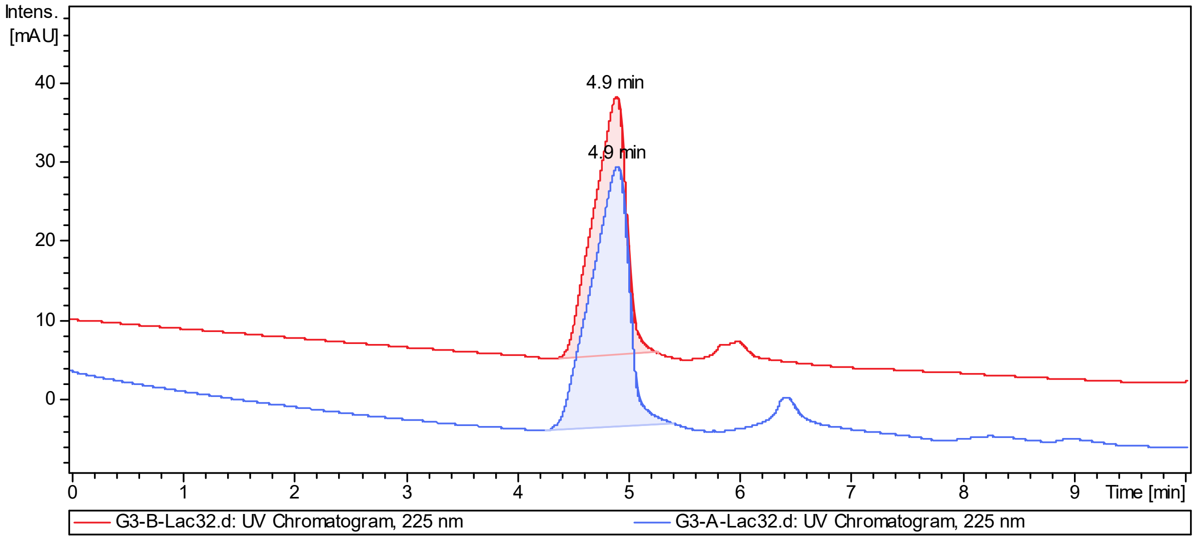

Figure S65: GPC trace of glycodendrimers: **G3-A-Lac32 (14b)** – blue, **G3-B-Lac32 (17b)** – red.

7. Galectin Characterization

The Bradford assay,[1] which utilizes Protein Assay Dye Reagent Concentrate (Bio-Rad Laboratories, Hercules, USA), was used to determine the galectin concentration. Bovine serum albumin (Sigma-Aldrich, Darmstadt, Germany) was used for the calibration of the assay solution. SDS-PAGE in a 12% gel was performed to analyze the purity and molecular weights of galectins. Approximately 2 µg of protein per lane was loaded onto the gel. Electrophoresis was carried out using a constant voltage of 130 V.

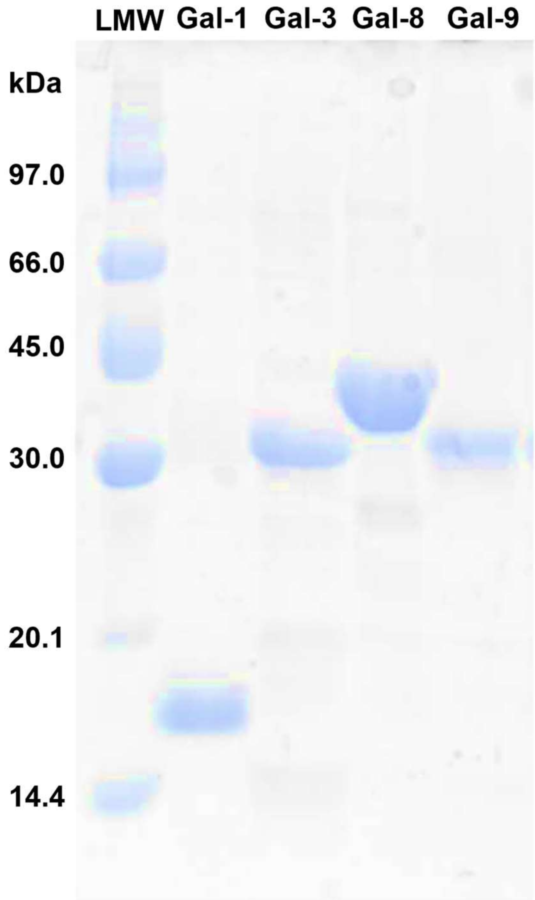

Figure S66. SDS-PAGE analysis of the prepared galectins. LMW – Amersham Low Molecular Weight Calibration Kit for SDS Electrophoresis (GE Healthcare, Chicago, USA): 97 kDa – phosphorylase b from rabbit muscle; 66 kDa – bovine serum albumin; 45 kDa – chicken egg-white ovalbumin; 30 kDa – carbonic anhydrase from bovine erythrocyte; 20.1 kDa – trypsin inhibitor from soybean; 14.4 kDa – α-lactalbumin from bovine milk. Molecular weights of galectins: Gal-1, 16.5 kD; Gal-3, 28.0 kDa; Gal-8, 38.0 kDa; Gal-9, 34.2 kDa. The band of Gal-3 migrates somewhat higher on the gel than expected, which, we presume, may be caused be the non-globular structure of the protein featuring a long N-terminal tail.

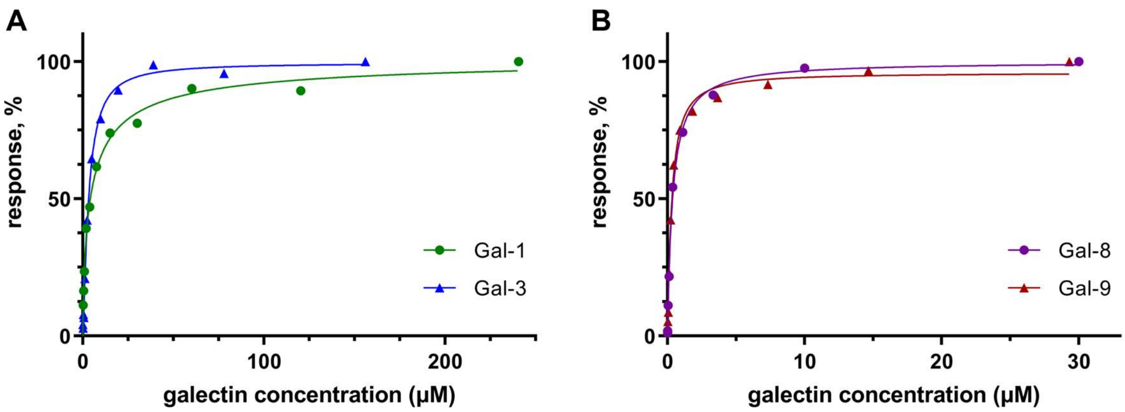

Figure S67. Direct binding of galectins to immobilized asialofetuin (ASF), A. Curves for Gal-1 and Gal-3, B. Curves for Gal-8, Gal-9. Respective K_D values: K_D = 4.4 μM for Gal-1, K_D = 3.2 μM for Gal-3, K_D = 0.37 μM for Gal-8, K_D = 0.30 μM for Gal-9. The experimental procedure of direct ELISA assay is analogous to the competitive ELISA assay detailed in the main text. In the direct ELISA assay, the incubation step comprised serial dilution of the respective galectin in EPBS (50 μL/well). A background value (as a mean of the negative control wells containing buffer instead of galectin/ligand, with absorbance of ca. 0.05-0.075) was subtracted from the measured absorbances. For Gal-1, higher concentration (240 μM) was used to have the curve fully saturated.

8. ELISA

Table S1. Inhibitory potency of glycodendrimers towards Gal-1, Gal-3, Gal-8, and Gal-9 recalculated per one lactosyl.

Sample	Valency	IC_{50}/lac [µM] [a]							
		Gal-1	*rp/lac* [b]	Gal-3	*rp/lac* [b]	Gal-8	*rp/lac* [b]	Gal-9	*rp/lac* [b]
lactose	1	310 ± 38	*1.0*	116 ± 26	*1.0*	1680 ± 420	*1.0*	1350 ± 440	*1.0*
G_1-A-Lac_8	8	1190 ± 190	*0.3*	750 ± 190	*0.2*	7370 ± 2310	*0.2*	260 ± 130	*5.1*
G_2-A-Lac_{16}	16	1110 ± 140	*0.3*	58 ± 15	*2.0*	1600 ± 280	*1.0*	75 ± 8.3	*18*
G_3-A-Lac_{32}	32	1430 ± 53	*0.2*	158 ± 69	*0.7*	2200 ± 830	*0.8*	77 ± 47	*17*
G_1-B-Lac_8	8	410 ± 150	*0.8*	97 ± 57	*1.2*	400 ± 250	*4.2*	135 ± 42	*10*
G_2-B-Lac_{16}	16	790 ± 230	*0.4*	88 ± 14	*1.3*	710 ± 330	*2.4*	39 ± 10	*34*
G_3-B-Lac_{32}	32	650 ± 270	*0.5*	94 ± 22	*1.2*	700 ± 67	*2.4*	31 ± 18	*44*

[a] IC_{50}/lac is the half maximal inhibitory potency of one lactosyl presented on the respective glycodendrimer. It is calculated as IC_{50} of the respective glycodendrimer (shown in Table 1) multiplied by the number of lactose units (equal to valency in a fully substituted conjugate), i.e., IC_{50}/lac = IC_{50} (glycodendrimer) × valency. [b] Relative inhibitory potency per lactosyl (*rp/lac*) is a ratio of the inhibitory potency of free lactose and the inhibitory potency of one lactosyl presented on the respective glycodendrimer; i.e., *rp/lac*= IC_{50} (lactose)/ (IC_{50}/lac).

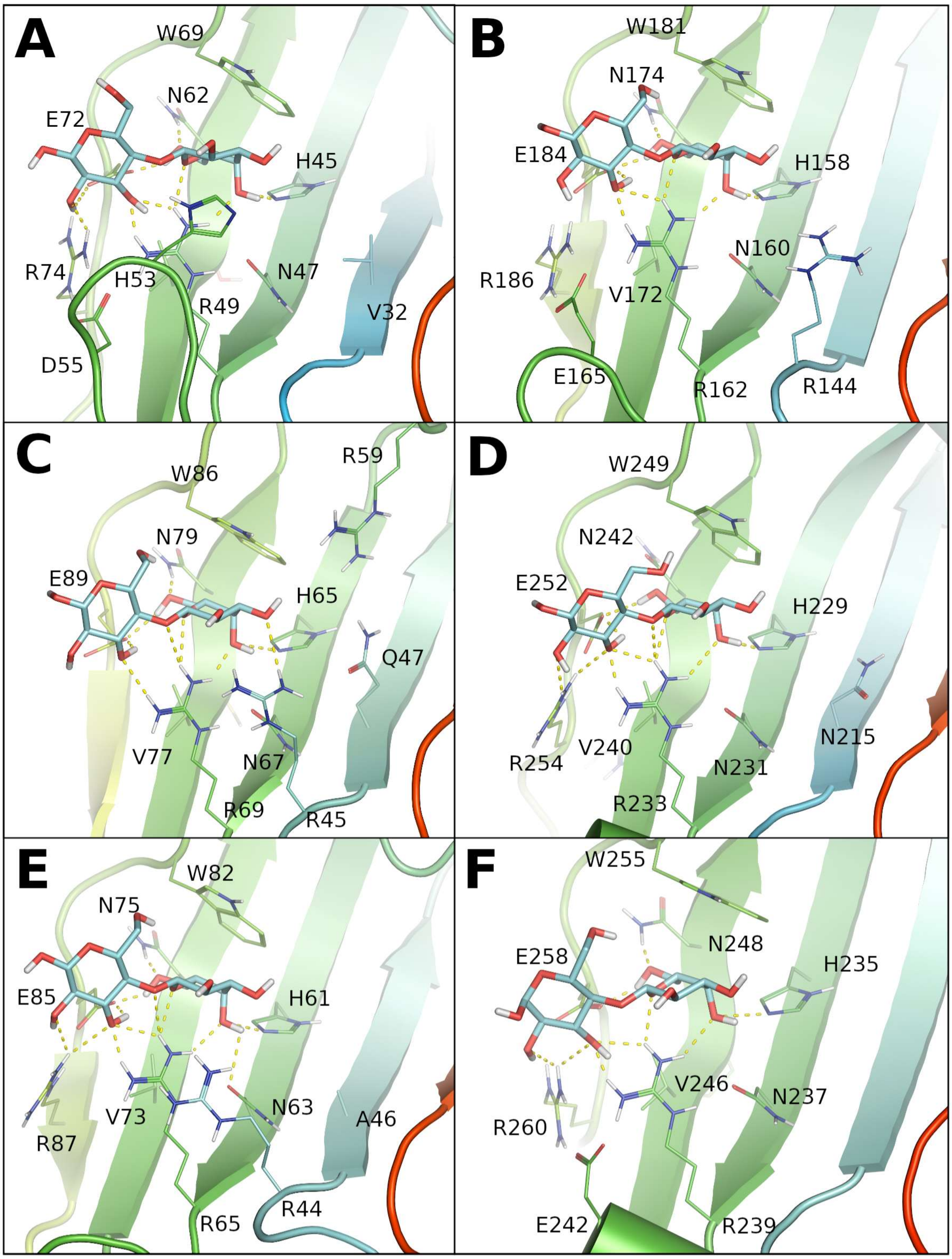

Figure S68. **A**. Gal-1 (PDB ID: 1W6O)[2]; **B**. Gal 3 (PDB ID: 4R9A)[3];**C**. Gal-8N (PDB ID: 5T7S); **D**. Gal-8C (PDB ID: 3VKL)[4]. **E**. Gal-9N (PDB ID: 3LSE)[5]; F. Gal-9C (PDB ID: 3WV6)[6]. Figures are made in PYMOL using the default mode. Residues within 3 Å from lactose ligand are shown. Hydrogen bonds are shown as dashes yellow lines. Residue numeration may sometimes differ in different publications.

9. Dynamic light scattering

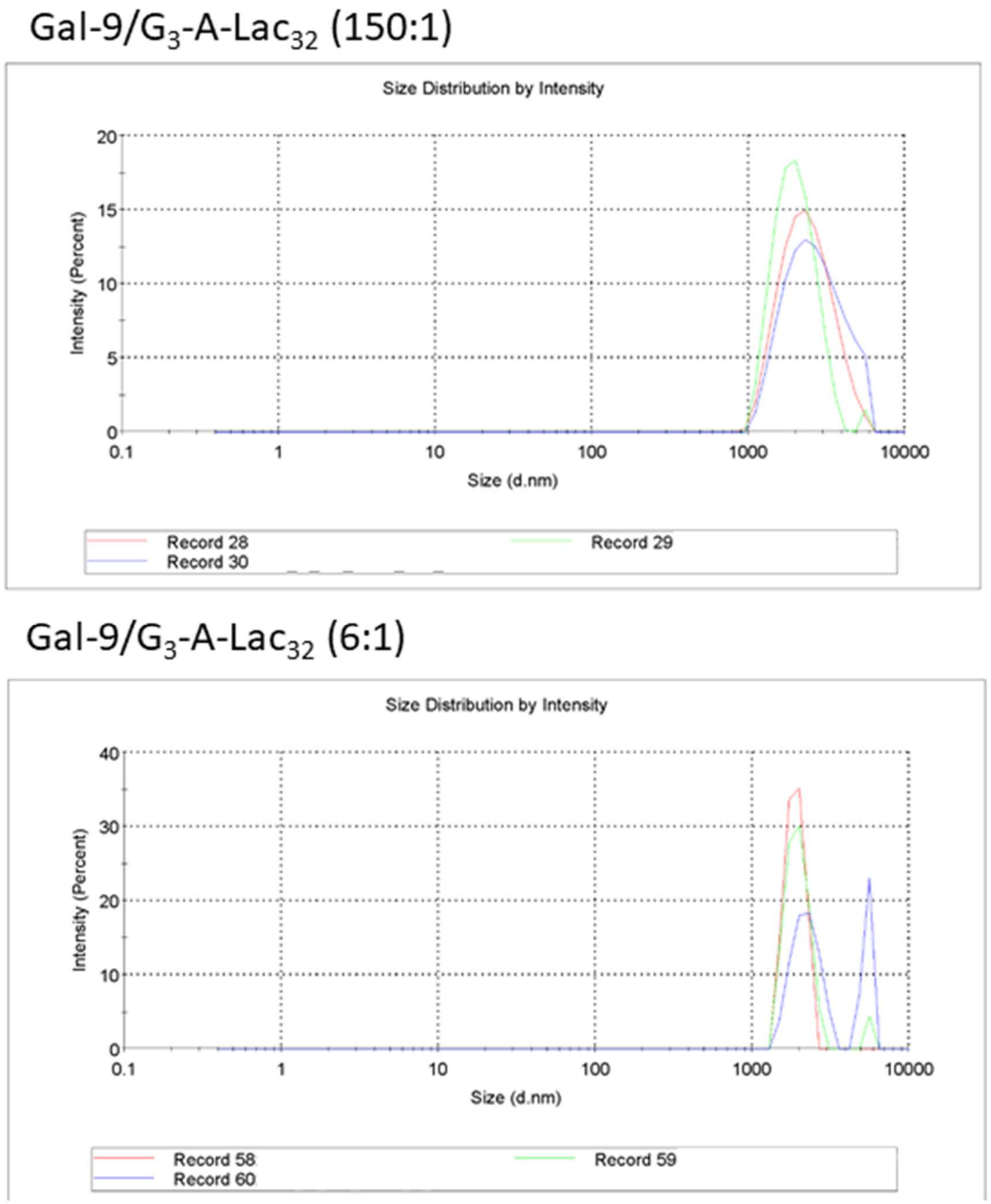

Figure S69. DLS of Gal-9/G_3-A-Lac_{32} (150:1) – top, Gal-9/G_3-A-Lac_{32} (6:1) – bottom.

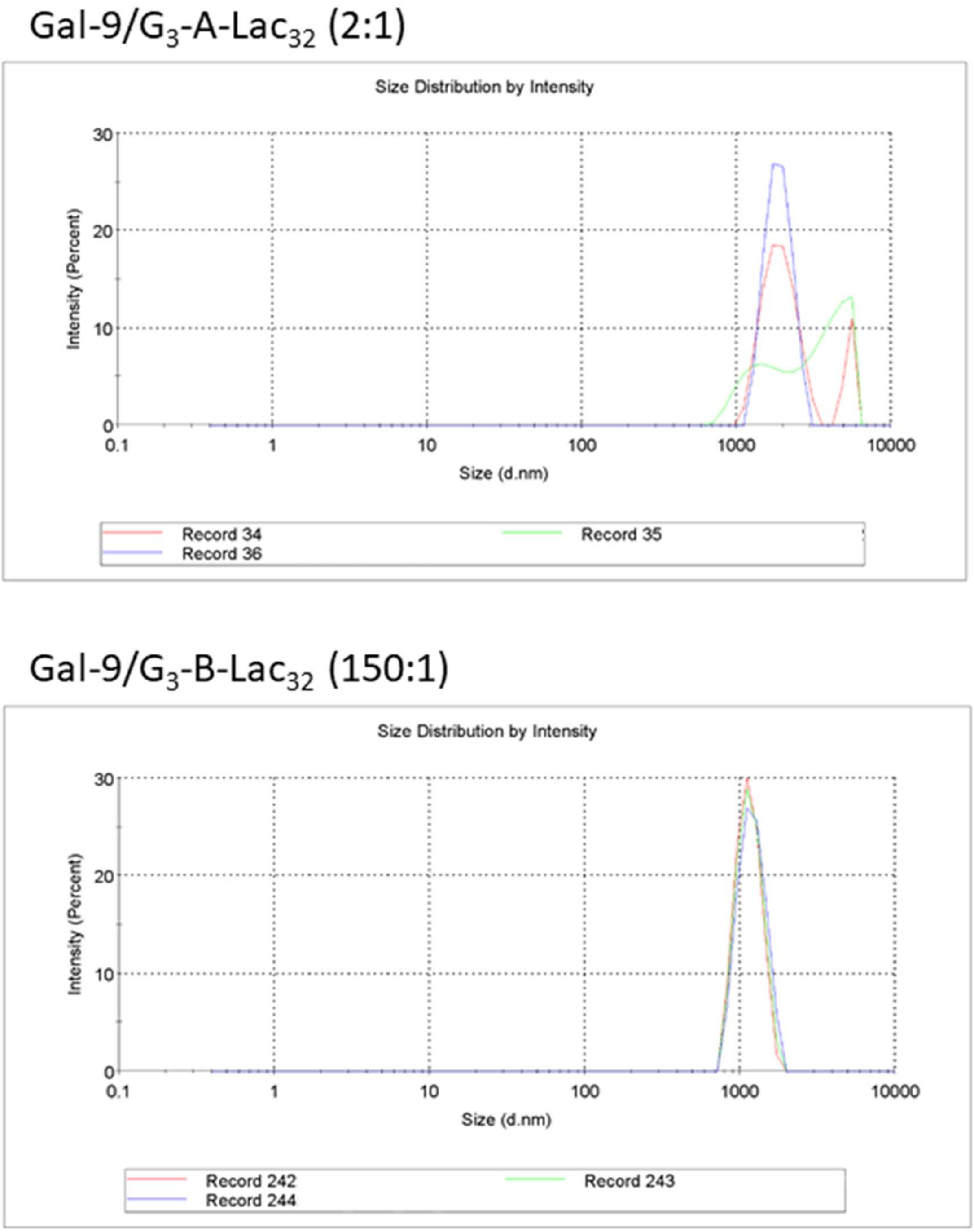

Figure S70. DLS of Gal-9/G_3-A-Lac_{32} (2:1) – top, Gal-9/G_3-B-Lac_{32} (150:1) – bottom.

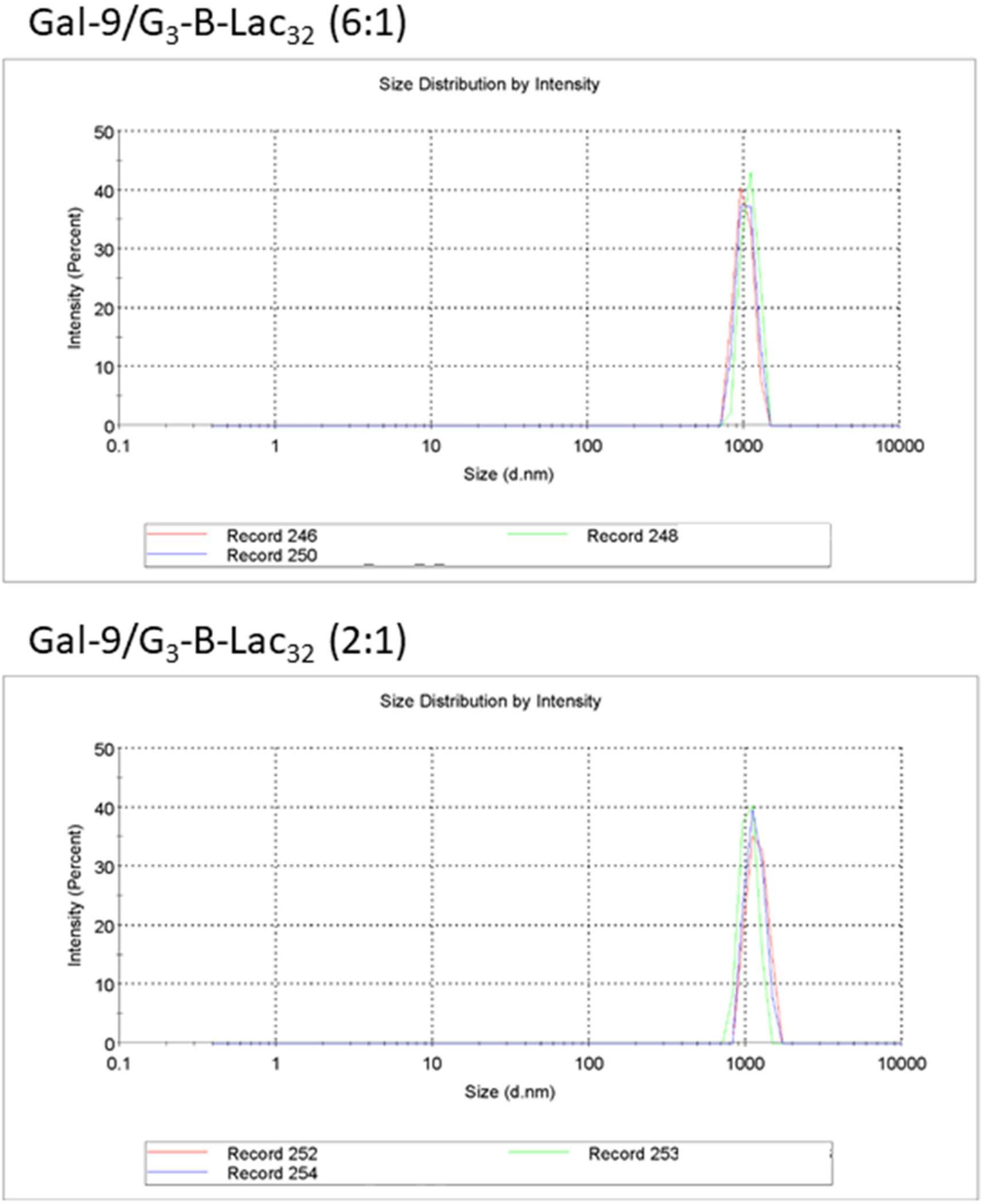

Figure S71. DLS of Gal-9/G_3-B-Lac_{32} (6:1) – top, Gal-9/G_3-B-Lac_{32} (2:1) – bottom.

References:

(1) Bradford, M. M. A Rapid and Sensitive Method for the Quantitation of Microgram Quantities of Protein Utilizing the Principle of Protein-Dye Binding. *Anal. Biochem.* **1976**, *72* (1–2), 248–254. https://doi.org/10.1016/0003-2697(76)90527-3.

(2) López-Lucendo, M. F.; Solís, D.; André, S.; Hirabayashi, J.; Kasai, K. I.; Kaltner, H.; Gabius, H. J.; Romero, A. Growth-Regulatory Human Galectin-1: Crystallographic Characterisation of the Structural Changes Induced by Single-Site Mutations and Their Impact on the Thermodynamics of Ligand Binding. *J. Mol. Biol.* **2004**, *343* (4), 957–970. https://doi.org/10.1016/j.jmb.2004.08.078.

(3) Su, J.; Zhang, T.; Wang, P.; Liu, F.; Tai, G.; Zhou, Y. The Water Network in Galectin-3 Ligand Binding Site Guides Inhibitor Design. *Acta Biochim. Biophys. Sin. (Shanghai).* **2015**, *47* (3), 192–198. https://doi.org/10.1093/abbs/gmu132.

(4) Yoshida, H.; Yamashita, S.; Teraoka, M.; Itoh, A.; Nakakita, S. I.; Nishi, N.; Kamitori, S. X-Ray Structure of a Protease-Resistant Mutant Form of Human Galectin-8 with Two Carbohydrate Recognition Domains. *FEBS J.* **2012**, *279* (20), 3937–3951. https://doi.org/10.1111/j.1742-4658.2012.08753.x.

(5) Solís, D.; Maté, M. J.; Lohr, M.; Ribeiro, J. P.; López-Merino, L.; André, S.; Buzamet, E.; Javier Cañada, F.; Kaltner, H.; Lensch, M.; Ruiz, F. M.; Haroske, G.; Wollina, U.; Kloor, M.; Kopitz, J.; Sáiz, J. L.; Menéndez, M.; Jiménez-Barbero, J.; Romero, A.; Gabius, H. J. N-Domain of Human Adhesion/Growth-Regulatory Galectin-9: Preference for Distinct Conformers and Non-Sialylated N-Glycans and Detection of Ligand-Induced Structural Changes in Crystal and Solution. *Int. J. Biochem. Cell Biol.* **2010**, *42* (6), 1019–1029. https://doi.org/10.1016/j.biocel.2010.03.007.

(6) Yoshida, H.; Nishi, N.; Wada, K.; Nakamura, T.; Hirashima, M.; Kuwabara, N.; Kato, R.; Kamitori, S. X-Ray Structure of a Protease-Resistant Mutant Form of Human Galectin-9 Having Two Carbohydrate Recognition Domains with a Metal-Binding Site. *Biochem. Biophys. Res. Commun.* **2017**, *490* (4), 1287–1293. https://doi.org/10.1016/j.bbrc.2017.07.009.

(7) DeLano WL (2002) The PyMOL MolecularGraphics System. DeLano Scientific, San Carlos, CA.http://www.pymol.org.
